# Modeled deposition of nitrogen and sulfur in Europe estimated by 14 air quality model systems: evaluation, effects of changes in emissions and implications for habitat protection

**DOI:** 10.5194/acp-18-10199-2018

**Published:** 2018-07-18

**Authors:** Marta G. Vivanco, Mark R. Theobald, Héctor García-Gómez, Juan Luis Garrido, Marje Prank, Wenche Aas, Mario Adani, Ummugulsum Alyuz, Camilla Andersson, Roberto Bellasio, Bertrand Bessagnet, Roberto Bianconi, Johannes Bieser, Jørgen Brandt, Gino Briganti, Andrea Cappelletti, Gabriele Curci, Jesper H. Christensen, Augustin Colette, Florian Couvidat, Cornelis Cuvelier, Massimo D’Isidoro, Johannes Flemming, Andrea Fraser, Camilla Geels, Kaj M. Hansen, Christian Hogrefe, Ulas Im, Oriol Jorba, Nutthida Kitwiroon, Astrid Manders, Mihaela Mircea, Noelia Otero, Maria-Teresa Pay, Luca Pozzoli, Efisio Solazzo, Svetlana Tsyro, Alper Unal, Peter Wind, Stefano Galmarini

**Affiliations:** 1 Environmental Department, CIEMAT, Madrid, 28040, Spain; 2 Finnish Meteorological Institute, Helsinki, FI00560, Finland; 3 Cornell University, Ithaca, NY, 14850, USA; 4 NILU-Norwegian Institute for Air Research, Kjeller, 2007, Norway; 5 ENEA, Italian National Agency for New Technologies, Energy and Sustainable Economic Development (ENEA), Via Martiri di Monte Sole 4, 40129 Bologna, Italy; 6 Bahcesehir University Engineering and Natural Sciences Faculty. 34353 Besiktas Istanbul, Turkey; 7 SMHI, Swedish Meteorological and Hydrological Institute Norrköping, Norrköping, Sweden; 8 Enviroware srl, Concorezzo, MB, Italy; 9 INERIS, Institut National de l’Environnement Industriel et des Risques, Parc Alata, 60550 Verneuil-en-Halatte, France; 10 Institute of Coastal Research, Chemistry Transport Modelling Group, Helmholtz-Zentrum Geesthacht, Germany; 11 Department of Environmental Science, Aarhus University, Roskilde, 4000, Denmark; 12 Department of Physical and Chemical Sciences, University of L’Aquila, L’Aquila, Italy; 13 Ex European Commission, Joint Research Centre (JRC), 21020 Ispra (Va), Italy; 14 European Centre for Medium-Range Weather Forecasts, Reading, UK; 15 Ricardo Energy & Environment, Gemini Building, Fermi Avenue, Harwell, Oxon, OX11 0QR, UK; 16 Computational Exposure Division, National Exposure Research Laboratory, Office of Research and Development, United States Environmental Protection Agency, Research Triangle Park, NC, USA; 17 BSC, Barcelona Supercomputing Center, Centro National de Supercomputacidn, Nexus II Building, Jordi Girona, 29, 08034 Barcelona, Spain; 18 Environmental Research Group, Kings’ College London, London, UK; 19 Netherlands Organization for Applied Scientific Research (TNO), Utrecht, the Netherlands; 20 IASS, Institute for Advanced Sustainability Studies, Potsdam, Germany; 21 European Commission, Joint Research Centre (JRC), Ispra (VA), Italy; 22 Climate Modelling and Air Pollution Division, Research and Development Department, Norwegian Meteorological Institute (MET Norway), P.O. Box 43, Blindern, 0313 Oslo, Norway; 23 Eurasia Institute of Earth Sciences, Istanbul Technical University, Turkey; 24 Faculty of Science and Technology, University of Tromsø, Tromsø, Norway

## Abstract

The evaluation and intercomparison of air quality models is key to reducing model errors and uncertainty. The projects AQMEII3 and EURODELTA-Trends, in the framework of the Task Force on Hemispheric Transport of Air Pollutants and the Task Force on Measurements and Modelling, respectively (both task forces under the UNECE Convention on the Long Range Transport of Air Pollution, LTRAP), have brought together various regional air quality models to analyze their performance in terms of air concentrations and wet deposition, as well as to address other specific objectives.

This paper jointly examines the results from both project communities by intercomparing and evaluating the deposition estimates of reduced and oxidized nitrogen (N) and sulfur (S) in Europe simulated by 14 air quality model systems for the year 2010. An accurate estimate of deposition is key to an accurate simulation of atmospheric concentrations. In addition, deposition fluxes are increasingly being used to estimate ecological impacts. It is therefore important to know by how much model results differ and how well they agree with observed values, at least when comparison with observations is possible, such as in the case of wet deposition.

This study reveals a large variability between the wet deposition estimates of the models, with some performing acceptably (according to previously defined criteria) and others underestimating wet deposition rates. For dry deposition, there are also considerable differences between the model estimates. An ensemble of the models with the best performance for N wet deposition was made and used to explore the implications of N deposition in the conservation of protected European habitats. Exceedances of empirical critical loads were calculated for the most common habitats at a resolution of 100 × 100 m^2^ within the Natura 2000 network, and the habitats with the largest areas showing exceedances are determined.

Moreover, simulations with reduced emissions in selected source areas indicated a fairly linear relationship between reductions in emissions and changes in the deposition rates of N and S. An approximate 20 % reduction in N and S deposition in Europe is found when emissions at a global scale are reduced by the same amount. European emissions are by far the main contributor to deposition in Europe, whereas the reduction in deposition due to a decrease in emissions in North America is very small and confined to the western part of the domain. Reductions in European emissions led to substantial decreases in the protected habitat areas with critical load exceedances (halving the exceeded area for certain habitats), whereas no change was found, on average, when reducing North American emissions in terms of average values per habitat.

## 1 Introduction

Improvements have been made in reducing ecosystem exposure to excess levels of acidification in past decades, largely as a result of declining SO_2_ emissions. However, in addition to acidification, emissions of NH_3_ and NO_*x*_ have altered the global nitrogen cycle, resulting in excess inputs of nutrient nitrogen into terrestrial and aquatic ecosystems (Maas and Grennfelt, 2016). This oversupply of nutrients can lead to eutrophication and subsequent loss of biodiversity. With the aim of ensuring the long-term survival of Europe’s most valuable and threatened species and habitats, the Natura 2000 network of protected areas (EEA, 2017) was established in Europe under the 1992 Habitats Directive (EU, 1992). While it is estimated that only 7 % of the total EU-28 ecosystem area and 5 % of the Natura 2000 area was at risk of acidification in 2010 (EEA, 2015), it is estimated that the fraction exposed to air pollution levels exceeding eutrophication limits was 63 and 73 %, respectively, in 2010 (EEA, 2015).

The Task Force on Hemispheric Transport of Air Pollution (HTAP) under the UNECE Convention on Long Range Transport of Air Pollution (CLRTAP) has organized several modeling exercises to understand the role of hemispheric transport when estimating the impacts of remote sources on background concentrations and deposition in different parts of the world (Galmarini et al., 2017). A description of the HTAP program can be found at http://www.htap.org/ (last access: 27 June 2018). While early exercises used global models, the most recent research activity, HTAP2, foresees a combination of global and regional models in order to evaluate air pollution impacts at a higher spatial resolution. In this context, the project AQMEII (Air Quality Model Evaluation International Initiative; Rao et al., 2011) in its third phase activity (AQMEII 3) has brought together various air quality modeling teams from North America and Europe to conduct a set of the simulations under the HTAP framework (Solazzo et al., 2017). At the same time, the EURODELTA-Trends (EDT) project has also brought together several European modeling teams to provide information for the Task Force on Measurements and Modelling (also under the CLRTAP), including the evaluation of models for specific campaigns (Bessagnet et al., 2016; Vivanco et al., 2017), and more recently for 20-year trends of air quality and deposition (Colette et al., 2017). Since both projects have a model evaluation component and there is a common simulation year (2010), it is possible to evaluate the datasets jointly, enabling the comparison of a larger number of models (eight for AQMEII3 plus seven for EDT).

The availability of 14-model simulations provides the possibility of obtaining a more robust ensemble model estimate of deposition than that from a single model, as well as an estimate of deposition uncertainty. This more robust estimate is particularly useful for assessing ecological impacts such as critical load exceedance. Critical loads (CLs) are limits for the deposition of atmospheric pollutants set by the working group on the effects of the CLRTAP for the protection of ecosystems (de Wit et al., 2015). Exceedances of CL have been utilized during the last decades to assess the impacts of atmospheric pollution on natural and seminatural European ecosystems. Moreover, applying empirical CL for nutrient N is recommended to assess “whether N deposition should be listed as a threat to future prospects” in the framework of the Habitats Directive 92/43/EEC (Henry and Aherne, 2014; Whitfield et al., 2011).

In addition to a model evaluation, we include an estimation of the exceedances of CL for the habitats in the European Natura 2000 network most threatened by N deposition. Moreover, in addressing one of the objectives of HTAP (Galmarini et al., 2017), we estimated the changes in wet deposition in Europe due to (1) a reduction of global emissions by 20 % or to a regional 20 % emission reduction solely in (2) North America or (3) Europe.

The paper is divided into seven main sections. Sections 2 and 3 focus on wet deposition, first describing the methodology used to evaluate model performance (Sect. 2) and then discussing the results (Sect. 3). Section 4 presents the intercomparison of dry deposition and in Sect. 5 we show the estimates from an ensemble of models for N and S. Next, in Sect. 6, we include an assessment of the influence of a 20 % reduction in emissions in Europe, North America and at a global scale on deposition in Europe. Finally, Sect. 7 provides an overview of the exceedances of the CL for the most threatened habitats in the Natura 2000 network using the ensemble estimates of deposition and shows the effect that the emission reductions presented in Sect. 6 has on them.

## 2 Methodology for the evaluation of wet deposition

This section describes the model simulations (2.1), the observations used for model evaluation (2.2) and the procedure to evaluate model performance (2.3).

Table 1 shows the description and abbreviations of the variables used in the assessment.

### 2.1 Model simulations

The simulations for the year 2010 used in this study were carried out using 14 air quality models (Table 2), 7 of them as part of AQMEII3 and the other 7 models participating in EDT. CHIMERE was involved in both projects, although the model version used in the EDT project is an improved (not yet official) version (Chimere2017b v1.0, Couvidat et al., 2018), and therefore a direct comparison of model results between the two simulations (AQMEII3 and EDT) is not possible. More modeling teams than those in Table 2 were involved in the AQMEII3 project, but we kept only those that provided all the variables required for the model performance evaluation in terms of wet deposition, i.e., air concentrations and deposition of related chemical species (except AQ_TR1_MACC, which only provided deposition data). The domain and grid resolution was common for all the models in EDT (except for ED_CMAQ, which used a different domain and projection), with a resolution of 0.25° (lat.) × 0.4° (lon.). AQMEII3 permitted a more flexible model setup, although outputs had to be produced for a fixed domain with a spatial resolution of 0.25° × 0.25°. Meteorological inputs for the AQMEII3 models were chosen by each participant (Table 2). In EDT, meteorological inputs from the Weather Research and Forecast model (WRF 3.3.1) were provided centrally, although not all models used this common dataset (WRF-Common). A more detailed description of the parameterizations of the meteorological models can be found in Solazzo et al. (2017) and Colette et al. (2017) for the AQMEII3 and ED exercises, respectively. In both exercises, boundary conditions were provided to the participants; in AQMEII3 they come from a global model, C-IFS(CB05) (Flemming et al., 2015), simulating the same scenarios at a spatial resolution 0.125° × 0.125° and providing results with a temporal resolution of 3 h. In EDT boundary conditions come primarily from observations combined with optimal interpolation and long-term trends, following the procedure used in the EMEP model (Simpson et al., 2006), with slight adjustments in the context of trend modeling (Colette et al., 2017). They were provided with a monthly time step at a spatial resolution of 1.5° × 1.5°.

Emissions were also prescribed in both projects: in AQMEII3 two options were available, Copernicus emissions (Pouliot et al., 2014) on a 0.125° × 0.0625° longitude-latitude grid and estimated for 2009 and HTAP_v2.2 emissions (Janssens-Maenhout, 2015) on a 0.1° × 0.1° grid, which for the European region are the same as the Copernicus inventory. In EDT ECLIPSE_V5 emissions estimated by the GAINS (Greenhouse gases and Air pollution INteractions and Synergies) model (Amann et al., 2011) for 2010 were used with a spatial resolution of 0.5° × 0.5° and regridded to 0.25° × 0.25° using the proxies of Colette et al. (2017). More information on the model setups can be found in Galmarini et al. (2017) and Solazzo et al. (2017) for AQMEII3 and Colette et al. (2017) for EDT.

Four simulations were carried out by the AQMEII3 community: a base case (BAS) for 2010; GLO, in which emissions were reduced at a global level by 20 %; EUR, in which emissions were reduced in Europe by 20 %; and NAM, in which emissions were reduced in North America by 20%. Not all the models performed the simulations for all four cases.

### 2.2 Observations

**Figure 1.**
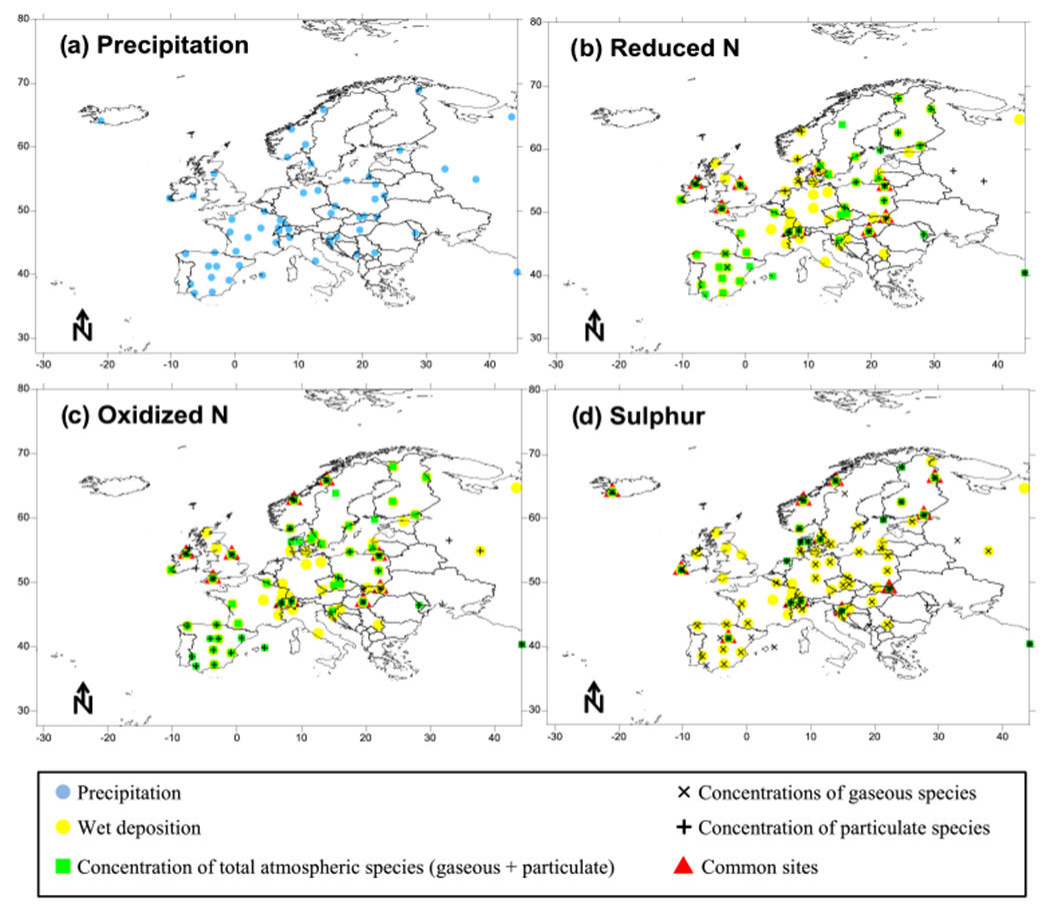
Monitoring sites with measurements of precipitation **(a)**, reduced N species **(b)**, oxidized N species **(c)** and S **(d)** used in the evaluation of annual modeled values.

Measurements (annual and monthly) made at 88 EMEP monitoring sites for 2010 were provided by the Norwegian Institute for Air Research (NILU), which is the chemical coordinating center of EMEP, although not all variables were measured at all sites. A complete description of the monitoring network of the EMEP program, as well as the sampling methodologies used can be found in Tørseth et al. (2012) and the data are openly accessible from http://ebas.nilu.no/ (last access: 29 June 2018). A summary of sites and variables considered is included in Table 3 and a map with their location is given in Fig. 1. Measurements for the gas phase (HNO_3_, NH_3_) are quite scarce, which makes it difficult to evaluate model performance for these species. For example, for annual values, more than two-thirds of the sites had measurements for both N and S deposition and atmospheric SO_2_ concentrations, while only 10 % had data for air concentrations of HNO_3_ and NH_3_. More sites than those for HNO_3_ and NH_3_ are measuring inorganic aerosols, though these are analyzed from PM_10_ samples in addition to the filter pack, which sample both aerosols and gases. One should be aware that the ${\mathrm{NH}}_{4}^{+}$ and ${\mathrm{NO}}_{3}^{-}$ concentrations might be underestimated due to the evaporation of ammonium nitrate from the particle filter to the gas filter, leading to a corresponding overestimate of the gas. This is the case for both PM_10_ and filter pack measurements, in which the separation of the nitrogen gases might be biased. The sum of HNO_3_ and ${\mathrm{NO}}_{3}^{-}$, as well as the sum of NH_3_ and ${\mathrm{NH}}_{4}^{+}$, however, are considered unbiased. The filter pack samplers usually have no size cutoff, but can be considered to be around PM_10_ (EMEP, 2014).

The spatial coverage of the observations used in the evaluation is quite high for most of northern, central and western Europe, including Spain, but is quite low in the eastern and southern regions (Fig. 1).

### 2.3 Evaluation

Model evaluation involved a joint analysis of wet deposition and air concentrations of the corresponding gas and particle species, as well as precipitation. Accumulated values were considered for precipitation and wet deposition, whereas mean values were used for air concentrations. Two different approaches were used when evaluating the model performance: (1) independently for each variable to have the largest number of available sites for each variable and (2) considering a common set of sites for the wet deposition and air concentrations of the respective gas and particle species for each deposition type, which are oxidized nitrogen (ON), reduced nitrogen (RN) and sulfur (S). Both annual and monthly values were evaluated.

For each model simulation and set of sites with observations, the following statistics were calculated (Table 4) for each variable (considering all the values in time and space): normalized mean squared error (NMSE), fractional bias (FB) and the fraction of model estimates within a factor of 2 of the observed values (FAC2). The acceptance criteria proposed by Chang and Hanna (2004, 2005) were used to assess model acceptability: FAC2 higher than or equal to 0.5, values of FB between −0.3 and 0.3, and NMSE values lower than or equal to 1.5. We define a model as performing acceptably for a particular variable when two out of these three criteria are met in recognition of the large uncertainties involved in these types of simulations (Hanna and Chang, 2010). It should be noted that the acceptability criteria adopted in this study had their origin in evaluating Gaussian atmospheric dispersion models rather than photochemical Eulerian grid models. However, due to the absence of established performance criteria for evaluating modeled atmospheric deposition, these criteria were nevertheless adopted in this study, while future work may be directed at developing performance goals more specifically tailored towards atmospheric deposition.

To illustrate the model performance for each variable, the three assessment statistics are shown on the same graph (“smile plots” hereafter) by plotting NMSE against FB and using a different symbol to indicate whether a model meets the acceptance criterion of Chang and Hanna (2004) for FAC2 (FAC2 ≥ 0.5). The statistics were calculated from annual and monthly data as well as by month in order to illustrate seasonal behavior. These smile plots include shaded areas that correspond to areas meeting the acceptance criteria of Chang and Hanna (2004) (blue for NMSE, red for FB). In addition, the theoretical minimum NMSE for a given value of FB is also plotted (parabolic dashed lines; Chang and Hanna, 2004). Additional statistics (mean gross error, MGE; normalized mean bias, NMB; normalized mean gross error, NMGE; root mean squared error, RMSE; correlation coefficient, *r*; coefficient of efficiency, COE; and index of agreement, IOA) were also calculated, as defined in the Supplement (Sect. S3.10).

In order to provide robust estimates of N and S deposition and their uncertainties for the calculation of critical load exceedances (Sect. 7), a multi-model ensemble was constructed using the mean and standard deviation of the total deposition for each grid cell calculated from the estimates of the best-performing models. A given model was included if it met at least two of the three acceptability criteria for wet deposition and gas and particle concentration considering the results for all the available sites and common sites. The main problem with this approach was that gas concentrations of NH_3_ and HNO_3_ were only measured at a few measurement sites. When these gas pollutants were the only ones failing to meet the criteria, we kept the model (ED_EMEP, AQ_FI_MACC and AQ_FI_HTAP) if the criteria for total concentrations was met (note that TNO_3_ and TNH_4_ were measured at some sites where no separate measurements of gas and particle air concentrations were made, and thus the model performance for these variables and TSO_4_ was only evaluated for all available sites).

## 3 Results and discussion for wet deposition

**Figure 2.**
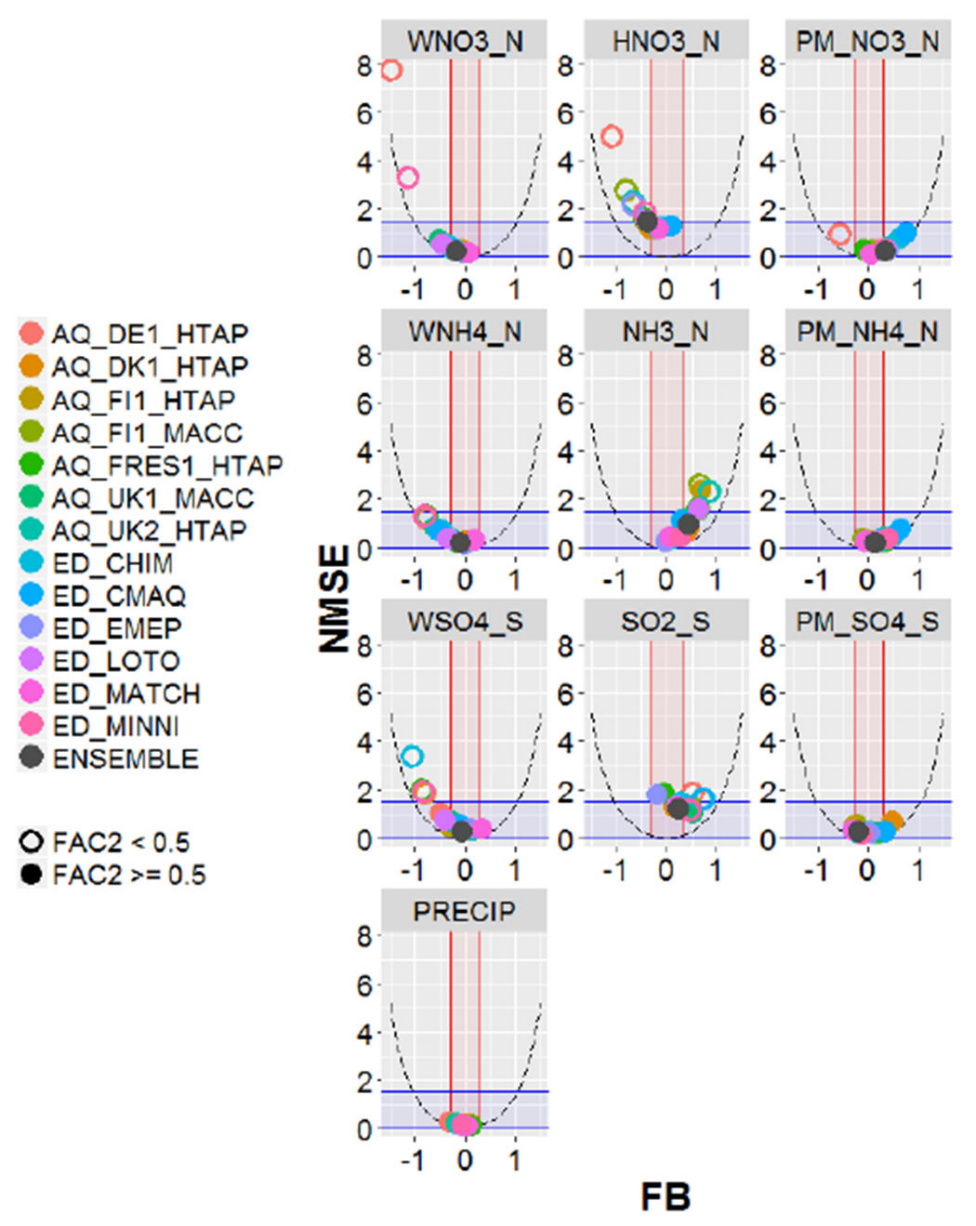
Statistics (FB, NMSE and FAC2) calculated from annual values of wet deposition, concentration and precipitation at all available sites. Shaded areas correspond to areas meeting the acceptance criteria of Chang and Hanna (2004) (blue for NMSE, red for FB). Parabolic dashed lines indicate the theoretical minimum NMSE for a given value of FB. Better model performance is indicated by points that fall within the blue and red shaded areas and with filled circles.

**Figure 3.**
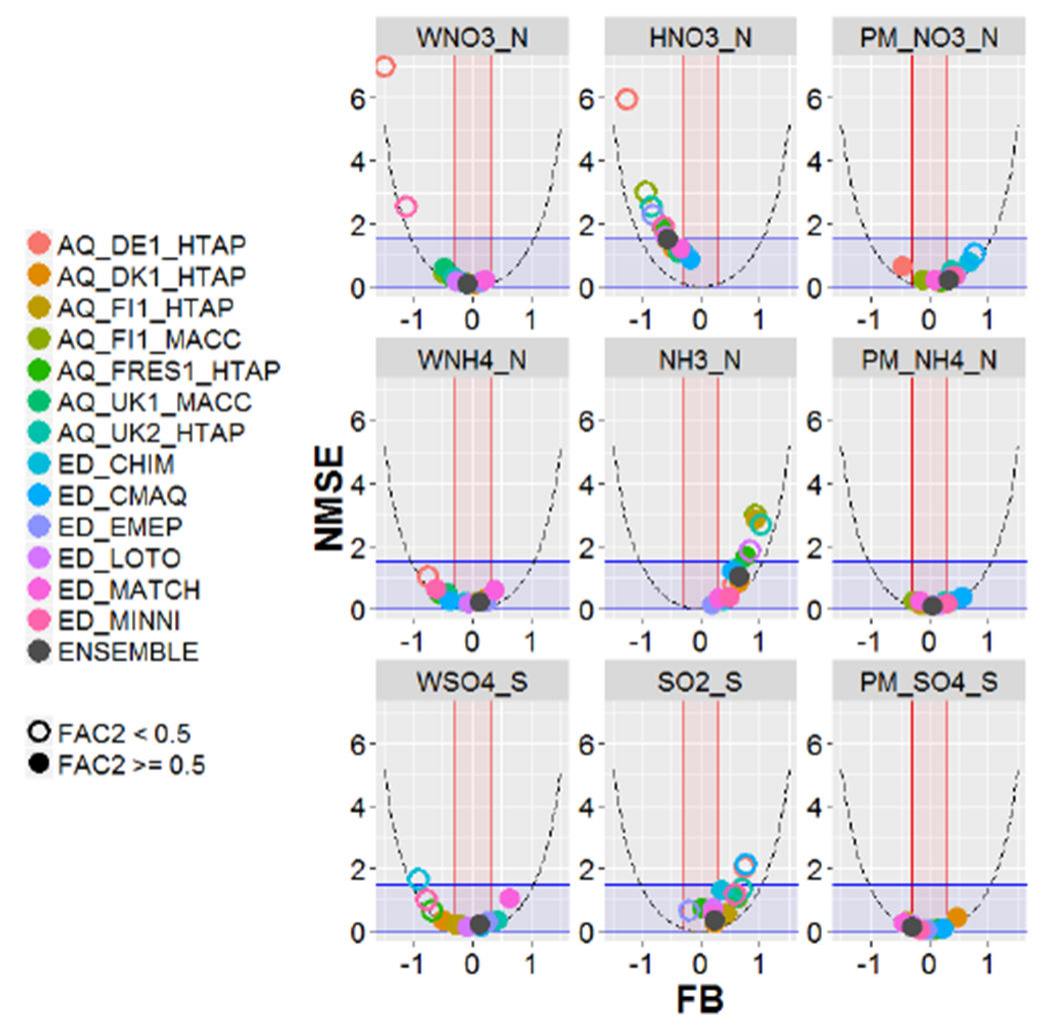
Statistics calculated from annual values (accumulated deposition or average means for air concentration) only at sites with simultaneous measurements of the three related pollutants (e.g., HNO_3_, PM_NO_3_ and WNO_3_) for oxidized N, reduced N and S species. Shaded areas correspond to areas meeting the acceptance criteria of Chang and Hanna (2004) (blue for NMSE, red for FB). Parabolic dashed lines indicate the theoretical minimum NMSE for a given value of FB. Better model performance is indicated by points that fall within the blue and red shaded areas and with filled circles.

The evaluation statistics for the selected models are provided in the Tables in Sect. S3.6. These results are represented visually in the smile plots in Fig. 2 (based on annual values for all sites) and Sect. S3.1 (based on monthly values), which also show the degree to which the acceptability criteria were met for all models. Figure 3 shows the smile plots considering only the common set of sites (sites with measurements of all the variables) to facilitate the analysis with regards to the interdependencies of model performance for different variables.

For precipitation, in general, monthly and annual accumulated precipitation rates estimated by the models agree reasonably well with the observations. The smile plots for precipitation in Fig. 2 and Sect. S3.1 (and the Tables in the Sect. S3.6) show that all the models meet all acceptability criteria, with the exception of AQ_DE1_HTAP, which narrowly misses the FB criterion for this variable. AQ_FRES1_HTAP had the lowest errors (NMSE) and the highest correlation with the observed precipitation values (*r*). Smile plots by month (Sect. S3.5) indicate that some models have a larger fractional bias in summer, especially in August when some models underestimate accumulated precipitation, particularly ED_LOTO, AQ_DE1_HTAP, AQ_UK1_MACC, AQ_UK2_HTAP and the three models using WRF_Common, which are ED_CHIM, ED_EMEP and ED_MINNI.

### 3.1 Oxidized nitrogen

In the case of WNO3_N (abbreviations in Table 1) a large variability was found (Sect. S1.2), with AQ_DE1_HTAP and ED_MINNI estimating the lowest values and AQ_TR1_MACC the highest. The smile plot in Fig. 2 (also included in Sect. S1.2 to facilitate interpretation) and the tables in Sect. S3.6 show that the models tended to underestimate the observed WNO3_N on average, with the exception of ED_EMEP, AQ_DK1_MACC, AQ_TR1_MACC and ED_MATCH with very low bias or even a slight overestimate. The results for ED_MINNI are consistent with the study by Vivanco et al. (2016), who evaluated several models (EMEP, CHIMERE, LOTOS-EUROS, MINNI, CMAQ and CAMX) for four 1-month campaigns during 2006, 2007, 2008 and 2009. Most of the models meet at least two of the three acceptability criteria for both monthly and annual wet deposition values, with the exception of AQ_DE1_HTAP and ED_MINNI, which substantially underestimated deposition. The underestimation of AQ_DE1_HTAP is continuous throughout the year, as shown in Sect. S3.2, whereas for ED_MINNI the underestimation is more pronounced in winter.

As shown in Sect. S3.6 all the models performed acceptably for TNO3_N, except AQ_DE1_HTAP for the monthly data and ED_CMAQ for the annual data. Interestingly, all the models performed worse for the atmospheric concentration of the gaseous form (HNO3_N) than for the particulate form (PM_NO3_N) (also visible in Fig. 3), with no model performing acceptably for the monthly data. The smile plots in Sect. S3.2 show the highest errors and underestimation of HNO3_N during winter. In fact, no model meets two criteria in January, February, March, November and December for this pollutant. Along the same lines, the box plots in Sect. S4 indicate an underestimation of the HNO_3_ : TNO_3_ ratio in winter for most of the models. Most models underestimate both WNO3_N and HNO3_N and overestimate PM_NO3_N for the winter period (October-March), which could suggest a too-efficient gas to particle conversion during these months in some cases, with a possibly low deposition efficiency for the particle phase. In the case of AQ_DE1_HTAP the underestimation of deposition, as well as the gas and particle air concentration, could be related to an underestimation of NO_2_ or HNO_3_ (via a low NO_2_ to HNO_3_ conversion rate). ED_EMEP overestimates WNO3_N and PM_NO3_N, but underestimates HNO3_N (according to annual values for common sites in Sect. S3.8), which could be related to a too-high gas deposition.

### 3.2 Reduced nitrogen

For WNH4_N there were also large differences between the models estimating the lowest values (AQ_DE1_HTAP, AQ_FRES1_HTAP and ED_MINNI) and those estimating the highest (AQ_TR1_MACC). Most of the models meet at least two of the three acceptability criteria for this pollutant, with the exceptions being AQ_DE1_HTAP, AQ_FRES1_HTAP and ED_MINNI. Similar to WNO3_N, Fig. 2 (also included in Sect. S1.1) and the tables in Sect. S3.6 show that the models tended to underestimate WNH4_N, with the exception of AQ_TR1_MACC and ED_MATCH. However, unlike WNO3_N, this underestimation seems to correlate with an overestimation of the gaseous form (NH3_N) on an annual basis (except for ED_EMEP, which has a very low bias for both pollutants, and ED_MATCH, which overestimates WNH4_N slightly). This is likely due to an underestimation of wet removal processes for the gas phase, but it can also be related to other issues, such as a general underestimation of NH_3_ dry deposition, an overestimation of emissions or even to measurement locations far from agricultural sources of ammonia and therefore not representative of the grid square. The overestimation of NH3_N mainly occurs in autumn and winter (January, February, November, December), as can be inferred from the monthly smile plots of NH3_N in Sect. S3.3, which shows a poorer model performance for this period (no model meets all three criteria).

It is interesting to see that this overestimation of NH3_N during November–January takes place when HNO3_N is underestimated, as discussed in the previous section, which could indicate an excessive conversion of HNO_3_ to particle due to an excess of NH_3_ (aerosol nitrate may be formed if enough ammonia is available) and favored with low temperatures. Ammonium is quite well reproduced, with all the models meeting the acceptance criteria both on an annual basis and a monthly basis. All in all, the tables in Sect. S3.6 indicate a general underestimation of wet deposition for reduced nitrogen, with a tendency to overestimate TNH4. There is more variability between the model estimates of the NH_3_ : TNH_4_ ratios for the winter months (Sect. S4) with the EDT models estimating lower ratios. It should be noted that some models do not distinguish between precipitation types and use the same scavenging rates for snow and rain, which could lead to substantial differences between model results.

At this point, we would like to make a comment on the interpretation for the gaseous species. In Sect. 2.2 we highlighted a potential problem of the evaporation of ammonium nitrate in the filter packs leading to a potential overestimation of the gas component in the measurement. If such an artifact occurred, it would tend to lead to an underprediction by the model for the gas component. However, we found that the models overestimate the concentrations of NH3_N, which cannot be attributed to this problem. However, it could be affecting the results of HNO3_N, for which models under-estimate concentrations. Nevertheless the evaporation-from-filters artifact should occur more strongly in summer, and the underestimation of models is observed mainly in winter, which suggests other reasons rather than a potential evaporation from filters. We should point out that, in addition to the problem of few sites measuring the gas component, the atmospheric lifetimes of HNO_3_ and NH_3_ are very short and so site representativeness is also a problem. More measurements of the gas-phase components would help in future evaluations of model performance.

### 3.3 Sulfur

Substantial differences were also found for WSO_4_, from the lowest values for ED_CHIM up to the highest for AQ_TR1_MACC and ED_MATCH. Most of the models meet at least two of the three acceptability criteria for WSO_4_, apart from AQ_DK1_HTAP, AQ_FRES1_HTAP, ED_CHIM and ED_MINNI. Similar to N deposition, the models tended to underestimate the observed values (Fig. 2), with the exception of AQ_TR1_MACC, AQ_UK2_HTAP, ED_EMEP and ED_MATCH. The tendency to underestimate WSO4_S by most models, similarly to the reduced nitrogen, is overall occurring simultaneously with an overestimation of the gaseous pollutant (SO2_S) on an annual and monthly basis. As shown in the monthly smile plots in Sect. S3.4, the underestimation of WSO4_S tends to be smaller (and even positive for some models) during the winter period (November–February). Unlike NH_3_ and HNO_3_, which have the largest model bias in winter, model bias for SO_2_ does not appear to have a seasonal dependence. Model performance is generally better for the particulate concentrations (PM_SO4_S) although some large errors occur in the winter (November–January). All models tended to overestimate TSO_4_, with the exception of ED_CHIM, ED_EMEP and ED_LOTO, and most models also tended to overestimate the SO_2_ : TSO_4_ ratios.

### 3.4 Joint discussion

In summary, wet deposition fluxes are generally underestimated for WSO4_S and WNH4_N and in winter in the case of WNO3_N. There are indications that the aqueous and heterogeneous chemistry (e.g., those involving the conversion of NO_*x*_ to HNO_3_) could be too slow or underrepresented in the models, especially in winter, as evidenced by an overestimation of primary gaseous pollutants, especially NH_3_ and SO_2_, for this period and an underestimation of the secondary pollutant HNO_3_ (formed via heterogeneous chemistry). However, this behavior (simultaneous overestimation of NH3_N and underestimation of HNO3_N in winter) could also be due to an excessive formation of nitrates (favored by low temperatures) due to a potential excess of NH_3_ (aerosol nitrate may be formed only if enough ammonia is available). This excess NH_3_ could be due to an overestimate of NH_3_ emissions during these months. The fact that sulfate concentrations are also low for several models in January and February and SO_2_ concentrations are somewhat high could be due to an underestimate of the conversion to aerosol (sulfate) via aqueous chemistry, which could be another cause of the excess NH_3_.

## 4 Model intercomparison of dry deposition

The figures in Sect. S2 show maps of dry deposition for oxidized nitrogen (ONDD) (Sect. S2.2), reduced nitrogen (RNDD) (Sect. S2.1), total N (Sect. S2.4) and S (Sect. S2.5). Unfortunately, not all the models participating in AQMEII3 provided the complete set of outputs, and therefore it was not possible to analyze the dry deposition estimates for all of them. For example, for reduced nitrogen, only estimates from AQ_FRES1_HTAP, AQ_UK2_HTAP and AQ_FI1* in AQMEII3 were available.

Maps of the dry deposition of total N for all models show the highest values over France, Germany and other central areas of the domain.

Differences between models can be seen in both high and low emission areas. Models have different deposition algorithms and, even when similar, they can have different input, such as land use or leaf index area. It would be interesting in future studies to analyze how different these parameters in the models are due to their importance in dry deposition estimates. The highest values of the dry deposition of total N (Sect. S2.4) are found for ED_CMAQ, with values higher than 1900 mg Nm^−2^ (annual accumulated value) over large areas in the central and western parts of the domain and mainly due to the contribution of oxidized species. AQ_FRES1_HTAP estimated the lowest values, whereas the rest of the model estimates have more similar spatial patterns. Maps in Sects. S2.1 and S2.2 for ONDD and RNDD indicate that ED_CMAQ estimates the highest values for both oxidized and reduced nitrogen dry deposition. The largest differences can be observed for ONDD, for which models in the AQMEII3 community estimate lower values, reflecting the lower emissions of NO_*x*_ used in these simulations (Sects. S7A and S7B). For RNDD differences between models are smaller, directly related to the more similar NH_3_ emissions. The highest values of RNDD are observed for the Netherlands, the western part of France, Denmark and Belgium, as well as some high values in the area of the Alps. This direct response of dry deposition to emissions is more apparent than for wet deposition, for which other factors such as precipitation act as essential drivers in addition to the varied wet scavenging parameterizations of models.

Significant differences can be found when looking at gas and particle deposition for the AQMEII3 participants (for ED information for the two phases was not available). Two gases, NO_2_ and HNO_3_, contribute to ONDD. As can be inferred from Sect. S2.3, in the case of AQ_DK1_HTAP and AQ_F11_HTAP the gas components (NO_2_ and HNO_3_) contribute more to ONDD than the particle phase, whereas in the case of AQ_TR1_MACC the largest contributions to ONDD come from the particle phase. This highlights the importance of taking measurements that can shed more light on these processes, providing modelers with data that can be used to parameterize and evaluate the different processes.

Spatial distributions are similar for the dry deposition of S (Sect. S2.5; higher values mainly over Poland, the Netherlands, United Kingdom, Germany and southeastern Europe), although in this case with higher differences in values, as can be inferred from the maps in Sect. S2.5. ED_CMAQ presents a different spatial pattern, with high values also over sea due to the consideration of sulfates coming from sea salt in this model application.

## 5 Ensemble

**Figure 4.**
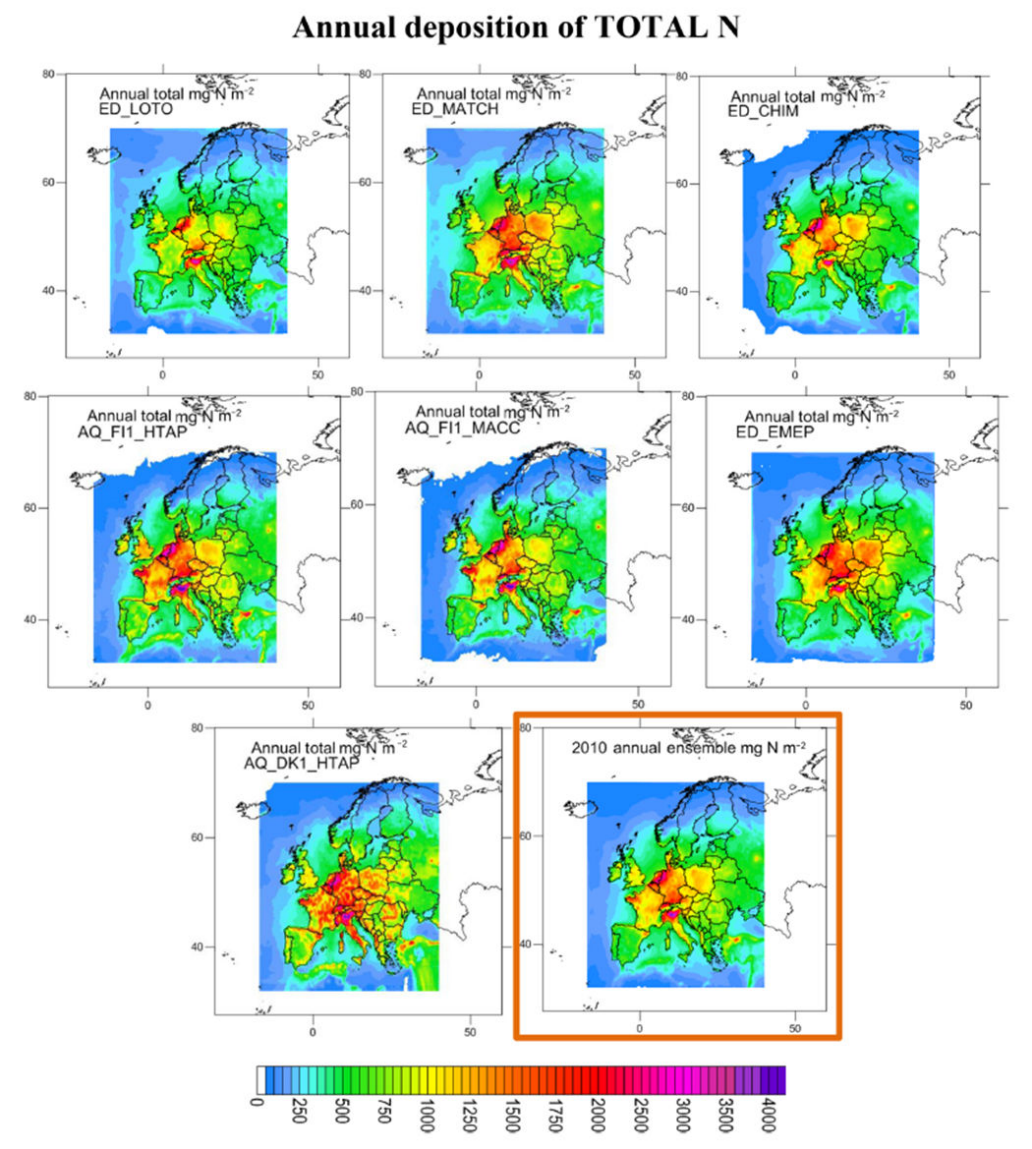
Maps of total N (mg N m^−2^) for the models showing acceptable performance for wet N deposition. The ensemble (mean of the models) is shown in the bottom right panel outlined in orange.

**Figure 5.**
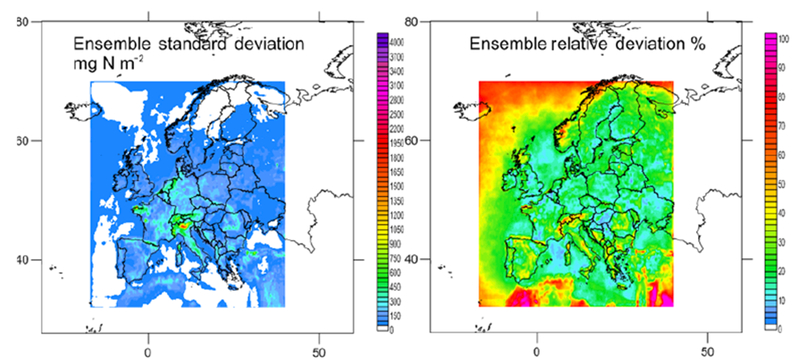
Maps of the standard deviation of total N in absolute and relative units (mg N m^−2^; % of annual mean) for the ensemble.

**Figure 6.**
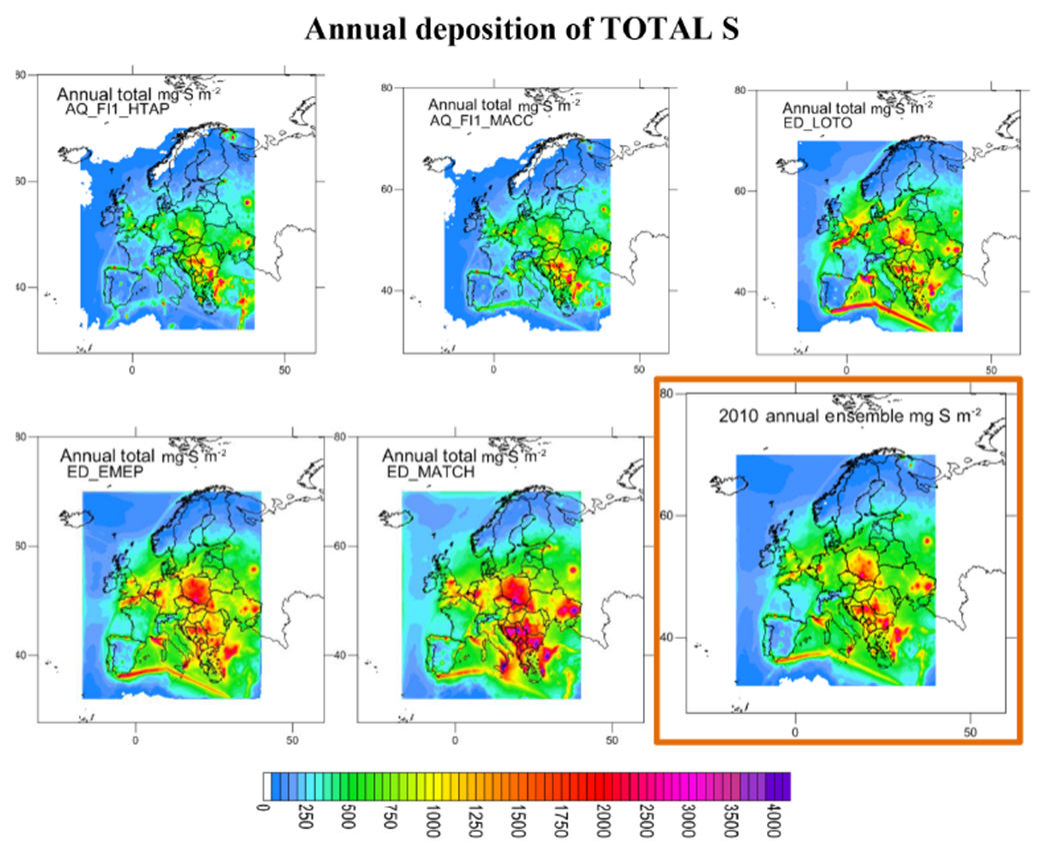
Maps of total S (mg N m^−2^) for the models showing acceptable performance for wet S deposition. The ensemble (mean of the models) is included (bottom right panel outlined in orange).

**Figure 7.**
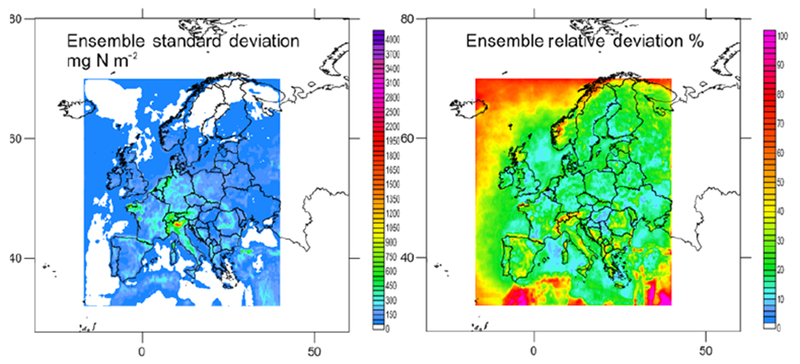
Maps of the standard deviation of total S in absolute and relative units (mg S m^−2^; % of annual mean) for the ensemble.

Considering the criteria in Sect. 2.1.3 and Tables S3.7 (calculated for all the available sites) and S3.8 (for common sites) jointly (that is, the criteria had to be met in both tables on an annual basis), the ensemble was composed of AQ_DK1_HTAP, ED_CHIM, ED_EMEP, ED_LOTO, AQ_FI1_MACC, AQ_FI1_HTAP and ED_MATCH for N deposition (considering both ON and RN at the same time; gridded information for AQ_UK1_MACC and AQ_UK2_HTAP, passing the acceptance criteria, was not available). For S deposition the models meeting the criteria for SO_2__S, PM_SO4_S and WSO4_S were ED_EMEP, ED_LOTO, ED_MATCH, AQ_FI1_HTAP, AQ_FI1_MACC and AQ_UK1_MACC (AQ_UK1_MACC gridded information was not available for all the variables, so it was not included in the ensemble). Figures 4 and 6 show the deposition of N and S for the selected models and the ensemble. The ensemble was calculated to facilitate the analysis in Sect. 7. Maps of annual wet deposition for all the models are shown in Sect. S1. Other criteria to select the models in the ensemble or methods to calculate would lead to a different ensemble. Figures 5 and 7 include maps of the standard deviation of total N and S, respectively, for the ensemble, calculated as shown in Table 4. For N deposition, the main differences are located in northern Italy (mainly due to the models estimating the largest deposition values in this region) and other areas, such as the Netherlands, for which there are notable differences in NO_*x*_ emissions between the ED and AQMEII3 simulations, and the Brittany region (northwestern France), where there are differences in ammonia emissions. For S deposition, the main differences are located over Poland and the English Channel and Mediterranean shipping routes, where there are differences between the SO_2_ emission inventories. Some of the models include volcanic emissions of SO_2_, which is why there are also large differences in S deposition close to the active volcano Etna on the island of Sicily (Italy).

Results for the ensemble are also included in smile plots and tables for wet deposition in order to show the performance of the ensemble.

## 6 Contribution of different regions (NA, EU, GLO) to N and S deposition in Europe

### 6.1 Methodology

As we have previously described in the framework of AQMEII3 activities and to give scientific support to the HTAP task force, research activities have included an evaluation of the influence of a reduction of emissions in some parts of the Northern Hemisphere on the air quality of other regions. Along these lines, some models ran simulations with (1) a 20 % reduction of global emissions (GLO), (2) a 20 % reduction of emissions in Europe (EUR) and (3) a 20 % reduction of emissions in North America (NAM). According to the acceptance criteria described in Sect. 2 and the availability of models running the different emission scenarios, we chose AQ_FI1_MACC as a representative model to demonstrate the effects of the different emission reduction scenarios. For WNO_3 the results from the AQ_FRES1_HTAP model were included as well, as this model performed acceptably for this pollutant and simulated the three perturbation scenarios.

The effect of each scenario was calculated in terms of deposition (mg N m^−2^) and percentage changes with respect to the base case (%). Differences between the base case simulation (no emission reduction) and the different scenarios were calculated for the wet and dry deposition of ON, RN and S, as well as for the total deposition of N and S.

### 6.2 Results

**Figure 8.**
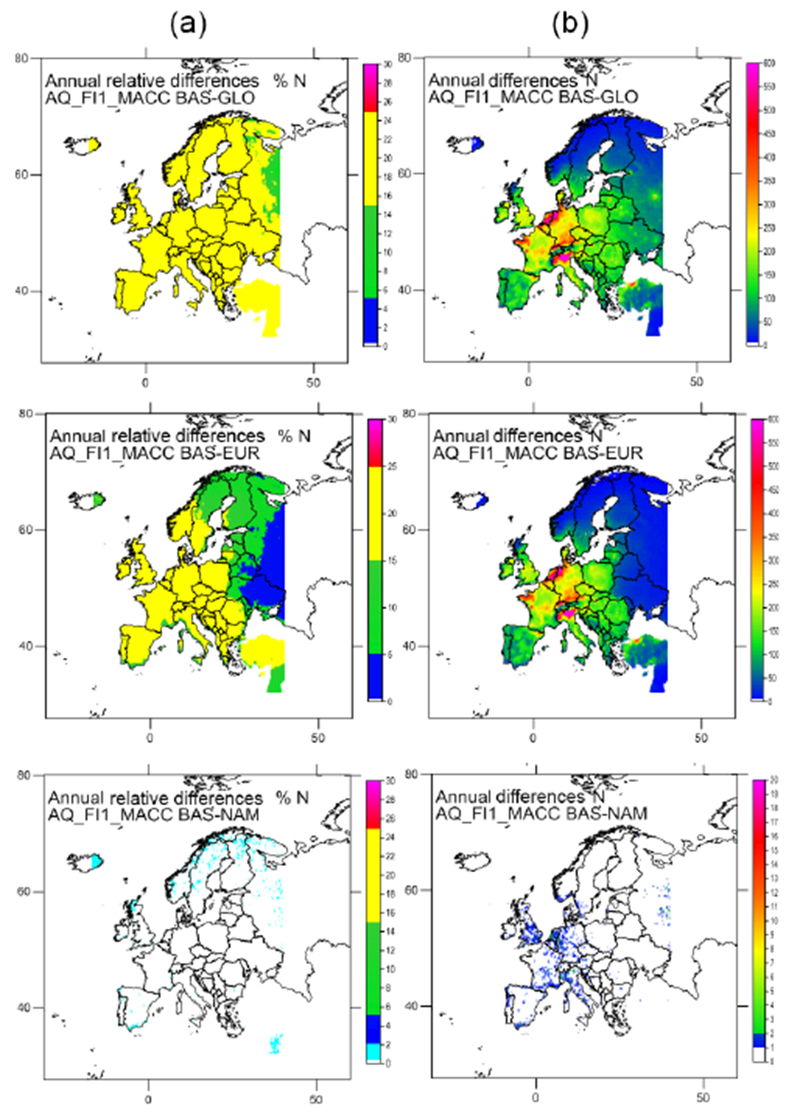
Effect on the N deposition in Europe of the 20 % reduction of emissions at global scale (GLO), in Europe (EUR) and in North America (NAM) according to AQ_FI1_MACC (%, **a**; mg N m^2^, **b**).

**Figure 9.**
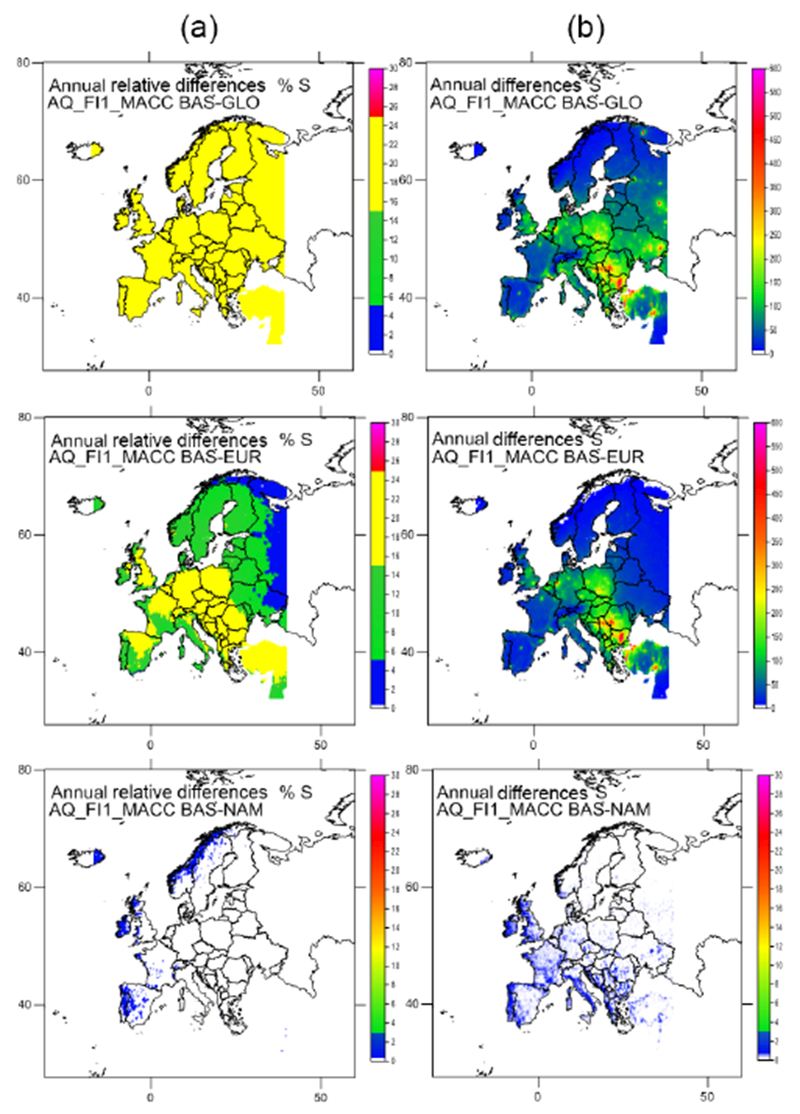
Effect on the S deposition in Europe of the 20 % reduction of emissions at global scale (GLO), in Europe (EUR) and in North America (NAM) according to AQ_FI1_MACC (%, **a**; mgN m^2^, **b**).

Maps reflecting the effect of the 20 % reduction of emissions in the different scenarios are included in Figs. 8 and 9 for total N and S (including both oxidized and reduced N, as well as wet and dry deposition) in absolute and relative terms. In general, a 20 % reduction of total N and S deposition is found when global emissions are reduced by 20 % (although somewhat lower for N in the United Kingdom, the Netherlands and Belgium). When a 20 % emission reduction is only applied in Europe, the deposition of N and S is decreased by 10–20 %. When emissions are reduced in North America only, deposition at the eastern areas of the domain is reduced by about 2 %, (Fig. 11). Im et al. (2018) also found an almost linear response to the change in emissions for NO_2_ and SO_2_ air concentration for the global perturbation scenario, with slighter smaller responses for the European perturbation scenario and a very small influence of long-range transport noticeable close to the boundaries.

Similar maps for wet and dry deposition are presented in Sects. S5 and S6. For WNO3_N the global emission reductions have the largest effect on European deposition, with the largest changes in wet deposition in the Alpine area (northern Italy, southern Germany). These areas are also affected in terms of WNH4_N, although in this case the emission reduction affects larger areas in Germany and the Netherlands. For WSO4_S (AM) the highest impacts are found on the Balkan Peninsula, especially the south of Bulgaria, Romania and Serbia. These quantities represent a reduction of about 20 % of the base case deposition in most parts of Europe, even a bit higher for WNO3_N in the Alpine area according to AQ_FI1_MACC. For AQ_FRES1_HTAP the reduction for WNO3_N is lower, in the range 14–20 % for the whole domain.

When emission reductions only occur in Europe, the changes in wet deposition are somewhat lower than for a global reduction according to AQ_FI1_MACC (Sects. S5.1, 5.2). Reductions in WNH4_N are similar to those of the global emission reduction scenario in western and central Europe, but substantially smaller in the eastern and northern parts of the domain, which are influenced more strongly by non-European emissions to the east. Larger differences are found between the global and European emission reduction scenarios for WNO3_N, with an influence of non-European emissions that extends throughout the domain. In many countries wet deposition decreases by about 10 % for the European emission reduction scenario, and a 20 % reduction is only found over some central areas. The situation is similar for WSO4_S, albeit with even larger contributions from non-European emissions. For AQ_FRES1_HTAP, the reduction of WNO3_N is similar to that estimated by AQ_FI1_MACC, although the range of reduction is smaller. Emission reductions in NA have a very small effect on European wet deposition (around 1–2 %), with reductions mostly concentrated in the western part of the domain (Iceland, Ireland, United Kingdom, Portugal, France, Spain, Norway). This pattern is also reproduced by AQ_FRES1_HTAP, although the absolute changes for AQ_FI1_MACC are larger in the central area and smaller on the Iberian Peninsula. The effect of global emission reductions on dry deposition is similar to that for wet deposition, although the relative reductions are slightly smaller for DNO3_N (except in the east and south of the domain) and slightly larger for DNH4_N and DSO4_S than for WNO3_N, WNH4_N and WSO4_S, respectively (Sects. S5, 6). The differences between the relative changes in wet and dry deposition are similar for the European emission reduction scenario, although the relative change is larger for the dry deposition in the east of the domain. The influence of emission reductions in NA on the wet deposition is generally larger than that on the dry deposition.

Differences between the global emissions reduction scenario and the European emission reduction scenario, discounting the effect of NAM, indicate that there is an influence of emissions from other regions, especially to the east of the domain, that could produce a 10 % reduction in deposition over certain areas. This is in agreement with results from studies carried out within the framework of the HTAP task force using global models, which estimate that 5–10 % of European N deposition is the result of non-European emissions (Dentener et al., 2011; Sanderson et al., 2008).

## 7 Deposition of N over areas in the Natura 2000 network

In this section, we first analyze the representativeness of the monitoring sites used in the evaluation of model deposition with a focus on habitat conservation. Secondly, the estimated deposition by the multi-model ensemble is used to evaluate the total N deposition (dry + wet) to the protected habitats. Finally, a simple evaluation (where possible) of the CL exceedances is presented. Together with S deposition, N deposition also contributes to acid deposition. However, as mentioned in the Introduction, only 5 % of the Natura 2000 area was at risk of acidification in 2010 and so the focus of this part of the study is on the exceedances of CLs for nutrient N.

### 7.1 Representativeness of monitoring sites for conservation purposes

**Figure 10.**
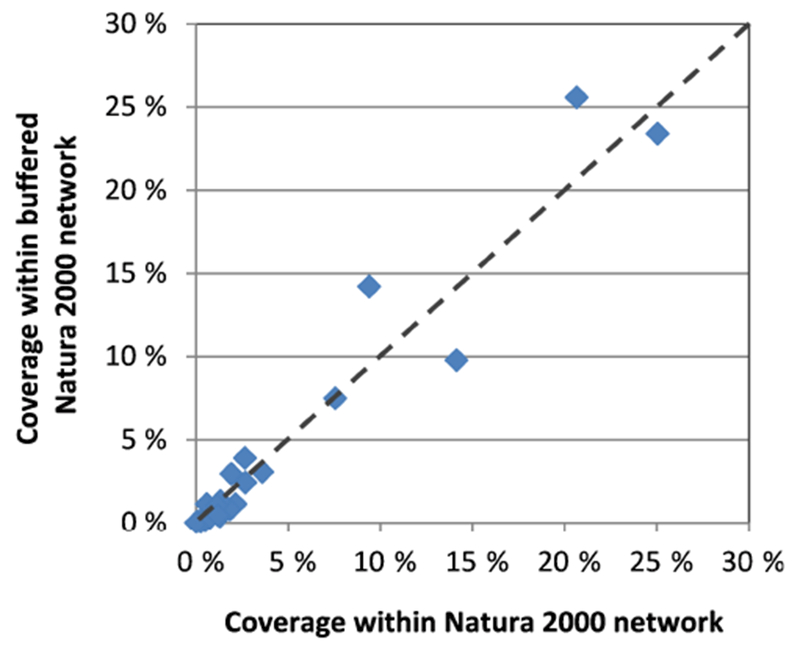
Coverage representation of EUNIS level-1 habitat classes within the entire Natura 2000 network versus the buffered areas.

The EMEP measurements are regionally representative (Tørseth et al., 2012; EMEP, 2014) and have historically been considered to represent an area larger than the size resolution of the EMEP atmospheric dispersion model (for the grid with 50 × 50 km^2^ of horizontal resolution). This resolution was taken as a reference for establishing a buffer zone of 2500 km^2^ around the receptors. The protected habitats inside the buffer zone were determined by intersecting the surface area of the Natura 2000 network (EEA, 2017) with the cover of the most likely habitats in Europe using EUNIS level-1 classification (EEA, 2015). Prior to this, aquatic, aquatic-related and anthropic habitats (such as gardens or arable lands) were excluded in order to study only natural and seminatural terrestrial ecosystems. The surface area covered by each habitat class included in the Natura 2000 network was plotted against the surface area of the same protected habitat classes within the abovementioned buffer zones in relative values with respect to their respective totals (Table 5, Fig. 10). The most represented terrestrial habitats in the entire network are broadleaved deciduous woodland, coniferous woodland, mesic grasslands and mixed deciduous and coniferous woodland (EUNIS classifications G1, G3, E2 and G4, respectively). The results indicate that the selected monitoring sites represent the main classes of terrestrial habitats fairly well, with G4 deviating most and an overrepresentation of 51 % within the protected buffered area with respect to the entire Natura 2000 network.

The same exercise was performed using only monitoring sites measuring all N species (including in precipitation, gaseous and particulate N). Only eight monitoring sites distributed between the United Kingdom, Switzerland and eastern Europe have the complete set of N pollutant measurements. Since the Natura 2000 network has no presence in Switzerland, only six sites could be evaluated for representativeness. Among the most represented habitats, G1 and G3 deviated the most in their representation. In any case, this subset can be considered small and poorly distributed across Europe. Therefore, the evaluation of model results for total concentration and the deposition of N pollutants in Europe is still far from being representative in terms of conservational purposes.

### 7.2 Risk assessment of atmospheric N deposition in the Natura 2000 network

The mean and standard deviation (SD) for the total deposition of N obtained from the ensemble model were combined with revised empirical CL (Bobbink and Hetteling, 2011) to provide a risk assessment of N deposition effects on vegetation in the Natura 2000 network. This evaluation constitutes a first approach, which helps to locate the most likely areas and major terrestrial habitat classes at risk of eutrophication as a result of atmospheric N deposition. Further research (particularly on habitat-specific CL) and a wider monitoring network (particularly to evaluate model performance for dry deposition) are needed to carry out a more accurate risk assessment. It is also interesting to bear in mind that even though recent studies (e.g., Cape et al., 2012; Izquieta-Rojano, 2016; Matsumoto et al., 2014) have highlighted the important contribution of the organic form to total N deposition (from 10 to more than 50 %), there are still important gaps in our knowledge of the role of the organic fraction in the N cycle and scarce attempts to include it in the measurement networks (e.g., Walker et al., 2012). The deposition of dissolved organic N constitutes another variable involving uncertainty in the actual understanding of the N cycle (Izquieta-Rojano et al., 2016) and consequently in the risk assessment of N deposition. Further research is therefore needed to understand the role that organic N plays in ecosystem functioning, biogeochemical cycles and even human health.

**Figure 11.**
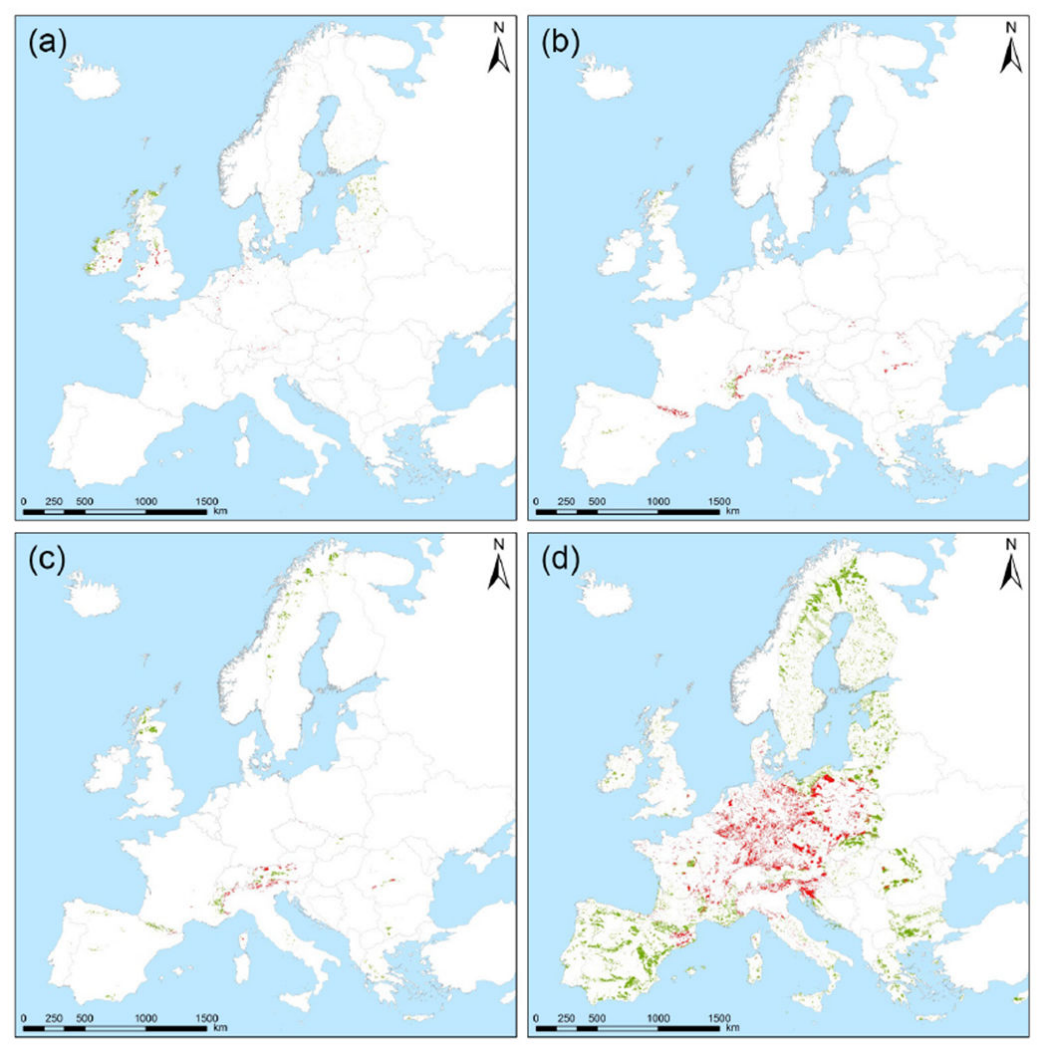
Habitat distribution and location of CL_exc_ for the most threatened habitat classes (**a**: D1 raised and blanket bogs and D2 valley mires, poor fens and transition mires; **b**: E4 alpine and subalpine grasslands; **c**: F2 arctic, alpine and subalpine scrub; **d**: G3 coniferous woodlands and G4 mixed deciduous and coniferous woodlands). The surface areas showing CL_exc_ are represented in red, while the areas with no CL_exc_ are represented in green.

**Figure 12.**
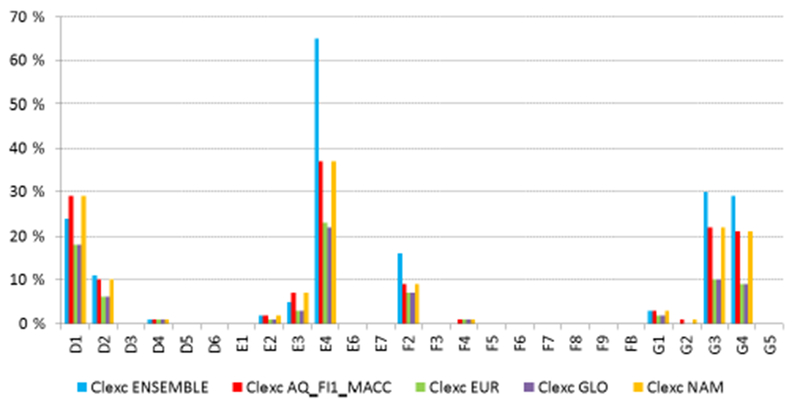
Proportion of habitat area for which the critical load is exceeded for major terrestrial habitat classes within the Natura 2000 network for the base case 2010 (ensemble and AQ_FI1_MACC) and for the EUR, GLO and NAM cases (AQ_FI1_MACC).

Ensemble deposition maps were projected and resampled to coincide with the EUNIS habitat grid (level-1 classification; ETRS89 LAEA projection; 100m × 100m cell size). The mean ± SD values were used as estimates of lower and upper uncertainty limits for the deposition, which were then compared to the mean CL attributed to each habitat class (Table 5; based on those from Bobbink and Hetteling, 2011). Those areas in which the class-attributed CL was exceeded by any of the values (mean SD; mean; mean + SD) were identified. The area presenting exceedances of empirical CL (CL_exc_) was summed for each EUNIS level-1 habitat class (Table 5). The areas showing CL_exc_ were mapped for the most threatened habitat classes (Fig. 11). In the case of similar habitats with similar distributions, a joint map is shown (D1 and D2; G3 and G4). Values of CL_ex_ in Fig. 12 indicate the area exposed to an exceedance of CL expressed as a percentage of the total area evaluated for each particular habitat class. These values were also calculated considering the total deposition of N from AQ_FI_MACC, as this model was used to estimate the variation in deposition due to changes in emissions, as will be explained later. All these operations were performed using ArcGIS 10.2 (ESRI; Redlands, CA, USA).

The six habitats with the largest surface area with a mean ensemble deposition above their respective CL were alpine and subalpine grasslands (E4), coniferous woodlands (G3), mixed deciduous and coniferous woodlands (G4), raised and blanket bogs (D1), arctic, alpine and subalpine scrub (F2) and valley mires, poor fens and transition mires (D2), with critical load exceedances covering 65, 34, 32, 24, 16 and 11 % of their respective areas (Table 5). Alpine and subalpine grasslands were also detected as the types most jeopardized by N deposition in a similar study for Spanish protected areas using 2008 simulations from EMEP and CHIMERE models (García-Gómez et al., 2014). These habitats are usually located in areas with complex topography where model estimates of atmospheric deposition can be more spatially inaccurate, as suggested in previous studies (e.g., García-Gómez et al., 2014; Simpson et al., 2006). The scarcity of monitoring sites at high altitude to evaluate model simulations can be considered as a major uncertainty in the risk assessment for N deposition.

The variation among the models included in the ensemble, represented here by the standard deviation (SD) of the ensemble, mostly affected E4 (Table 5). The reduction of the area at risk in this habitat class is remarkably high (−50 %) when the lower limit of the deposition is used (mean SD; Table 5). This might indicate that the CL is exceeded in most areas by a narrow margin. Within the other five habitat classes with the highest CL_exc_ area, the area at risk decreased by 13 % and increased by 16 % on average when the lower and upper limits of deposition are used. These same six habitats were again found to present the largest areas showing CL_exc_ when using AQ_FI1_MACC estimates, although some differences were found (Fig. 12).

Apart from the uncertainty in modeled deposition, the uncertainty in the CL attributed to the habitat classes should also be considered. On the one hand, some CLs proposed in the CLRTAP revision are based on expert judgment (e.g., those for E2, F5 or G4) and some were averaged from those proposed for several subclasses (e.g., for E1 and F4). On the other hand, even when the proposed CLs are reliable and match perfectly with the habitat classes evaluated in this study, an adjustment linked to more local conditions is recommended (e.g., for D1 it is recommended to vary the applied CL as a function of the precipitation range or the water table level). However, since a CL averaged from the proposed range was used for each habitat class and the evaluation was performed on a broad scale, we consider the results suitable for the purpose of this work, which is highlighting the protected areas and terrestrial habitats with the highest probability of suffering eutrophication. Finally, the use in this approach of a modeled dry deposition that is in fact weighted for the different land use inside each grid cell might lead to an underestimation of, for instance, forest risks, as the dry deposition for plant surfaces is higher than for other land uses and it is currently smoothed during the weighting process. To perform a more accurate assessment, habitat-type-specific values for the dry deposition of N are necessary. It is therefore recommended that chemical transport models provide dry deposition data as a function of leaf area index (LAI) or habitat type in order to be more suitable for risk assessment studies.

We also estimated how much the reductions in emissions described in Sect. 6 affected the risks of N impacts in the Natura 2000 areas. As can be inferred from Fig. 12, there is a significant reduction in the habitat area experiencing CL_exc_ for the scenarios GLO and EUR compared with the base case (AQ_FI1_MACC). Particularly, the most jeopardized habitat types showed a reduction of more than a third in their overall threatened area. Both reduction scenarios showed almost similar values of CL_exc_, with only slight differences in E4 (for which GLO reduction produces a slightly larger decrease in CL_exc_). G3 and G4 habitats are the most affected, for which the exceeded area was approximately halved as a result of the emission reduction. In the case of NAM, no decrease is observed, indicating the low impact of hemispheric transport from North America to Europe, at least in terms of N deposition in 2010.

## 8 Conclusions

A comparison of the wet and dry deposition of N and S estimated by 14 air quality models participating in the projects AQMEII3 and EURODELTAIII revealed considerable differences between the models. An evaluation of model performance was carried out, jointly considering air concentrations and wet deposition of the relevant compounds. Very few measurements of gaseous species (HNO_3_ or NH_3_) were available, making it difficult to do a fair and complete evaluation.

In general, for oxidized N wet deposition, most of the models meet at least two of the three acceptability criteria (NMSE < 1.5, |FB| < 0.3, FAC2 > 0.5) for both monthly and annual wet deposition values, with the exceptions of AQ_DE1_HTAP and ED_MINNI, which substantially underestimated deposition. In the case of AQ_DE1_HTAP this is a behavior occurring throughout the whole year and to some extent related to an underestimation of precipitation in this model. For ED_MINNI the underestimation of WNO3_N is more evident in winter and is not related to precipitation, which has a better agreement with observations during this period. All the models performed acceptably for TNO3_N, except for AQ_DE1_HTAP for the monthly data and ED_CMAQ for the annual data. All the models performed worse for atmospheric concentrations of the gaseous form (HNO3_N) than for the particulate form (PM_NO3_N), with no model performing acceptably for the monthly data and most models underestimating the HNO_3_ : TNO_3_ ratio during the winter months. It is, however, important to note that the observations of independent ${\mathrm{NO}}_{3}^{-}$ and HNO_3_ are not measured with an unbiased method (same as NH_3_ and ${\mathrm{NH}}_{4}^{+}$), so it is difficult to draw strong conclusions on the model performance for these compounds.

For reduced N wet deposition, there was a general underestimation, which seems to correlate with an overestimation of the gaseous form (NH3_N) on an annual basis (except for ED_EMEP, which has a very low bias for both pollutants, and ED_MATCH, which overestimates WNH4_N slightly). The overestimation of NH3_N is mainly observed in autumn and winter (January, February, November, December). Most models tend to underestimate WSO4_S, with the exception of AQ_TR1_MACC, AQ_UK2_HTAP, ED_EMEP and ED_MATCH. The underestimation of WSO4_S tends to be smaller (and even positive for some models) during the winter period (November-February), when there is a tendency by most models to overestimate the gaseous pollutant (SO2_S).

Considering the whole picture, wet deposition fluxes are generally underestimated for WSO4_S and WNH4_N and in winter in the case of WNO3_N. During the winter period, the results indicate an overestimation of primary gaseous pollutants, especially NH_3_ and SO_2_, and an underestimation of the secondary pollutant HNO_3_. Several factors can explain this behavior, such as too-slow or underrepresented aqueous and heterogeneous chemistry (e.g., those involving the conversion of NO_*x*_ to HNO_3_) and/or an overestimate of NH_3_ emissions during these months leading to an excessive decrease in HNO_3_ through the formation of nitrates (aerosol nitrate may be formed only if enough ammonia is available). The fact that sulfate concentrations are also low for several models in January and February and those of SO_2_ are somewhat high could be due to an underestimate of the conversion to aerosol (sulfate) via aqueous chemistry, which could be another cause of the excess NH_3_. More detailed studies would be needed to better understand the specific problems of each model, taking into account the multiple processes involved and all the relevant chemical and meteorological variables.

For dry deposition, large differences were found between the models, highlighting the importance of obtaining measurement data to evaluate model performance. This point is important considering the significant contribution of dry deposition to total deposition.

A multi-model ensemble was constructed using the better-performing models for wet deposition (N and S) and having also estimated dry deposition. For N, the ensemble was produced as the mean of AQ_FI1_MACC, AQ_FI1_HTAP, AQ_DK1_MACC, ED_EMEP and ED_MATCH models and was used to calculate exceedances of empirical critical loads for nitrogen in habitats in the European Natura 2000 network. Six habitats were identified as having critical load exceedances covering more than 10 % of their total area: alpine and subalpine grasslands (E4), coniferous woodlands (G3), mixed deciduous and coniferous woodlands (G4), raised and blanket bogs (D1), arctic, alpine and subalpine scrub (F2) and valley mires, poor fens and transition mires (D2), with critical load exceedances covering 60, 30, 29, 22, 13 and 10 % of their respective areas. The variation among the ensemble models in terms of the standard deviation of the ensemble mostly affected E4, with 85 % of the habitat area exceeded for the upper deposition estimate. It is important to point out that in addition to the uncertainty in modeled deposition, the CL attributed to a given habitat is also uncertain. Extending the deposition monitoring networks in European mountains would not only be beneficial for the study of atmospheric deposition, but also for model evaluation and risk assessment for these particularly threatened areas.

The 20 % reduction of emissions at global scale produces a 20 % reduction in the total deposition of N and S, with the main contributor being Europe according to the estimates of the A_FI1_MACC model. This reduction of total deposition is directly related to a decrease in CL_exc_ found for the different habitats in the Natura 2000 network, especially for G3 and G4, for which the exceeded area was approximately halved as a result of the emission reduction. The hemispheric transport of air pollutants from NAM has a low impact on wet deposition, mostly concentrated over the Atlantic area.

*Data availability.* The modeling and observational data generated for the AQMEII exercise are accessible through the ENSEMBLE data platform (http://ensemble3.jrc.it/, last access: 26 June 2018) upon contact with the managing organizations. References to the repositories of the observational data used have been also provided in Sect. 2. For the EURODELTA project simulations, technical details allowing forthcoming replication of the experiment are available on the wiki of the EMEP Task Force on Measurement and Modelling10 and that also provides ESGF links to corresponding input forcing data (see Colette et al. 2017 for more details and conditions). More information on the observational database is shown in Sect. 2.2.

## Supplementary Material

Supp

## Figures and Tables

**Table 1.** Abbreviations used in this publication. Note that “_N” or “_S” is added when referring to specific values that are calculated in terms of N or S.

Wet deposition of oxidized N	WNO_3_	WNO3_N
Wet deposition of reduced N	WNH_4_	WNH4_N
Wet deposition of S	WSO_4_	WSO4_S
Dry deposition of oxidized N	DNO_3_	DNO3_N
Dry deposition of reduced N	DNH_4_	DNH4_N
Dry deposition of S	DSO_4_	DSO4_S
Atmospheric concentration of N from nitric acid	HNO_3_	HNO3_N
Atmospheric concentration of N from nitrate in PM_10_	PM_NO_3_	PM_NO3_N
Total oxidized N concentration, HNO_3_ + PM_NO_3_	TNO_3_	TNO3_N
Atmospheric concentration of N from ammonia	NH_3_	NH3_N
Atmospheric concentration of N from ammonium in PM_10_	PM_NH_4_	PM_NH4_N
Total reduced N concentration, NH_3_ + PM_NH_4_	TNH_4_	TNH4_N
Atmospheric concentration of S	SO_2_	SO2_S
Atmospheric concentration of S from sulfate in PM_10_	PM_SO_4_	PM_SO4_S
Total S concentration, SO_2_ + PM_SO_4_	TSO_4_	TSO4_S
Precipitation	PRECIP	

**Table 2.** Meteorological model and CTM used by each participant. More specific information regarding both meteorological and chemical transport models is included in Solazzo et al. (2017) and Colette et al. (2017).

	AQMEII3		EDT		

	METEO*	CTM*		METEO**	CTM**
AQ_DE1_HTAP	COSMO-CLMy	CMAQ (v4.7.1)	ED_CHIM	WRF-Common***	CHIMERE (Chimere2017b v1.0)
AQ_DK1_HTAP	WRF (v 3.6)	DEHM	ED_CMAQ	WRF-Common (adapted to different projection)	CMAQ (v5.0.2)
AQ_FI1_HTAP/_MACC	ECMWF	SILAM	ED EMEP	WRF-Common	EMEP (rv4.7)
AQ_FRES1_HTAP	ECMWF	CHIMERE (vchim2013)	ED_LOTO	RACMO2	LOTOS (v1.10.005)
AQ_UK1_MACC	WRF (v3.4.1)	CMAQ (v5.0.2)	ED_MATCH	HIRLAM	MATCH (VSOA April 2016)
AQ_UK2_HTAP	WRF (v3.5.1)	CMAQ (v5.0.2)	ED MINNI	WRF-Common	MINNI (V4.7)
AQ_TR1_MACC	WRF (v3.5)	CMAQ (v4.7.1)			

EMISSIONS: Copernicus 0.125° × 0.0625°-HTAP_v2.2 0.1° × 0.1°; annual and monthly.			EMISSIONS: ECLIPSE_V5, 0.5° × 0.5°, regriddedto 0.25° × 0.25°; annual.		

BOUNDARY CONDITIONS: C-IFS (CB05), 0.125° × 0.125°; every 3 h.			BOUNDARY CONDITIONS: 1.5° × 1.5°; monthly.		

* More information in Solazzo et al. (2017).

** More information in Colette et al. (2017).

*** As defined in Colette et al. (2017).

**Table 3.** Number of sites for each pollutant.

WNO_3_: 59	TNO_3_: 45	HNO_3_: 12	PM_NO_3_: 32
WNH_4_: 61	TNH_4_: 39	NH_3_: 12	PM_NH_4_: 27
WSO_4_: 61	TSO_4_: 18*	SO_2_: 57	PM_SO_4_: 21

* Calculated as the addition of SO_2_ to PM_SO_4_; not directly measured using filter packs.

**Table 4.** The three metrics relating modeled concentrations (*M*) with the observed values (*O*) used for evaluating model performance in the smile plots and standard deviation for the ensemble.

NMSE	$\mathrm{NMSE}=\frac{\bar{{\left(O-M\right)}^{2}}}{\bar{O}\bar{M}}$	< = 1.5
FB	$\mathrm{FB}=\frac{2(\bar{M}-\bar{O})}{\left(\bar{O}+\bar{M}\right)}$	|FB| < = 0.3
FAC2	Fraction of model estimates within a factor of 2 of the observed values 0.5 ≤ $\frac{M}{O}$ ≤ 2.0	FAC2> = 0.5
SD	$\mathrm{SD}=\sqrt{\frac{1}{N-1}\sum_{i=1}^{N}{\left(M_{i}-\bar{M}\right)}^{2}}$	*N*: number of models in the ensemble $\bar{M}$ : ensemble, mean of models

**Table 5.** Coverage, mean ensemble deposition, attributed critical load and its exceedances (considering the mean and the mean plus or minus the standard deviation of the ensemble deposition) for the main terrestrial habitat classes within the Natura 2000 network.

Habitat group	EUNIS code	Habitat class	Natura 2000^a^	Receptors^b^	Avg. dep (kgN ha^−1^)^c^	CL (kgN ha^−1^)^d^	CL_exc_^e^	Cl_exc_ (Dep.-SD)^f^	Cl_exc_ (Dep. + SD)^f^
Peatlands	D1	Raised and blanket bogs	1.9%	2.9%	5.98	7.50	24%	13%	37%
	D2	Valley mires, poor fens and transition mires	0.2%	0.1 %	6.94	12.50	11 %	7%	16%
	D3	Aapa, palsa and polygon mires	2.1%	1.1 %	1.49				
	D4	Base-rich fens and calcareous spring mires	0.1%	0.1 %	9.02	21.25	1 %	0%	2%
	D5	Sedge and reedbeds	0.5%	0.3%	8.05				
	D6	Inland saline and brackish marshes and reedbeds	<0.1%	<0.1 %	11.34				

Grasslands	El	Dry grasslands	0.5%	0.1 %	5.41	15.75	0%	0%	0%
	E2	Mesic grasslands	14.1%	9.8%	9.02	20.00	2%	1%	3%
	E3	Seasonally wet and wet grasslands	1.8%	0.8%	8.83	16.25	5%	2%	10%
	E4	Alpine and subalpine grasslands	1.3%	1.3%	8.40	7.50	65%	15%	85%
	E6	Inland salt steppes	0.5%	0.1 %	7.60				
	E7	Sparsely wooded grasslands	1.3%	0.4%	5.24				

Shrublands	F2	Arctic, alpine and subalpine scrub	2.7%	3.9%	5.07	10.00	16%	5%	32%
	F3	Temperate and Mediterranean-montane scrub	3.6%	3.1 %	4.25				
	F4	Temperate shrub heathland	<0.1%	<0.1 %	4.67	15.00	0%	0%	1 %
	F5	Arborescent and thermo-Mediterranean brushes	2.7%	2.4%	6.11	25.00	0%	0%	0%
	F6	Garrigue	0.6%	1.1 %	6.39				
	F7	Spiny Mediterranean heaths	1.1%	1.1 %	5.72				
	F8	Thermo-Atlantic xerophytic scrub	0.3%	0.0%	nd				
	F9	Riverine and fen scrubs	<0.1%	<0.1 %	4.15				
	FB	Shrub plantations	0.8%	0.3%	7.63				

Woodlands	G1	Broadleaved deciduous woodland	25.1%	23.4%	8.50	15.00	4%	1%	14%
	G2	Broadleaved evergreen woodland	1.2%	0.4%	6.88	15.00	0%	0%	5%
	G3	Coniferous woodland	20.7 %	25.6 %	7.83	10.00	34%	14%	53%
	G4	Mixed deciduous and coniferous woodland	9.4%	14.2%	8.61	10.75	32%	13%	58%
	G5	Early-stage woodland and seminatural stands	7.6%	7.5%	6.16	7.50			

a Representation within the Natura 2000 network;

b representation within the Natura 2000 network in the joint of the buffered areas;

c weighted mean of N deposition for each habitat class according to ensemble results;

d attributed critical load in this work (based on empirical critical loads from Bobbink and Hetteling, 2011);

e area experiencing an exceedance of the CL, expressed as percentage of the total area evaluated for each particular habitat class;

f area experiencing an exceedance of the CL when using an ensemble deposition value of the mean plus or minus the standard deviation of the ensemble mean.
