# Supplementary material for: Modeled deposition of nitrogen and sulfur in Europe estimated by 14 air quality model systems: evaluation, effects of changes in emissions and implications for habitat protection
Source: Atmos Chem Phys. Author manuscript; Available in PMC 2018 Nov 15. (PMC6235743; doi:10.5194/acp-18-10199-2018)

Supplement of Atmos. Chem. Phys., 18, 10199–10218, 2018
https://doi.org/10.5194/acp-18-10199-2018-supplement
© Author(s) 2018. This work is distributed under
the Creative Commons Attribution 4.0 License.

Supplement of

Modeled deposition of nitrogen and sulfur in Europe estimated by 14 air quality model systems: evaluation, effects of changes in emissions and implications for habitat protection

Marta G. Vivanco et al.

Correspondence to: Marta G. Vivanco (m.garcia@ciemat.es)

The copyright of individual parts of the supplement might differ from the CC BY 4.0 License.

SUPPLEMENT

S1. MAPS OF WET DEPOSITION FOR DIFFERENT COMPOUNDS

S1.1 Annual deposition of WNH4_N

S1.2 Annual deposition of WNO3_N

S1.3 Annual deposition of total wet N (WNH4_N + WNO3_N)

S1.4 Annual deposition of WSO4_N

S2. MAPS OF DRY DEPOSITION FOR DIFFERENT COMPOUNDS

S2.1 Annual deposition of DNH4_N

S2.2 Annual deposition of DNO3_N

S2.3 Annual dry deposition of the gas and particle components contributing to DNO3_N

S2.4 Annual deposition of total dry N (DNH4_N + DNO3_N)

S2.5 Annual deposition of DSO4_S

S3. STATISTICS FOR WET DEPOSITION FOR DIFFERENT COMPOUNDS

S3.1 Statistics from monthly values (SMILE PLOTS)

S3.2 Statistics by month (FB, NMSE and FAC2) for oxidised-N species (SMILE PLOTS)

S3.3 Statistics by month (FB, NMSE and FAC2) for reduced-N species (SMILE PLOTS)

S3.4 Statistics by month (FB, NMSE and FAC2) for S species (SMILE PLOTS)

S3.5 Statistics by month (FB, NMSE and FAC2) for precipitation (SMILE PLOTS)

S3.6
Tables with complete statistics from monthly values. All available sites.

S3.7 Tables with complete statistics from annual values. All available sites.

S3.8 Tables with complete statistics from annual values. Common sites.

S3.9 Monthly values of WNO3_N, WNH4_N,WSO4_S and precipitation Definition

S3.10 Definition of statistical metrics in S3.6, 3.7 and 3.8

S4. Contribution of gas and particles phases

S5. Effect on wet deposition in Europe of a reduction of 20% of emissions 1) at a global scale (GLO), 2) in Europe (EUR) and 3) in North America (NAM)

S5.1. EFFECT ON WET DEPOSTION IN EUROPE OF A REDUCTION OF A 20% OF GLOBAL EMISSIONS (GLO) FOR WNH4_N (left), WNO3_N (middle) and WSO4_S (right), ACCORDING TO AQ_FI1_MACC

S5.2. EFFECT ON WET DEPOSTION IN EUROPE OF A REDUCTION OF A 20% OF EUROPEAN EMISSIONS (EUR) FOR WNH4_N (left), WNO3_N (middle) and WSO4_S (right), ACCORDING TO AQ_FI1_MACC

S5.3. EFFECT ON WET DEPOSTION IN EUROPE OF A REDUCTION OF A 20% OF NORTH-AMERICAN EMISSIONS (NAM) FOR WNH4_N (left), WNO3_N (middle) and WSO4_S (right), ACCORDING TO AQ_FI1_MACC

S5.4. Effect on Europe of the reduction of 20% of emissions at a global scale (left maps), in Europe (middle) and in North America (right) for WNO3_N, according to HT_FRES1_HTAP in terms of wet deposition

S6. Effect on dry deposition in Europe of a reduction of 20% of emissions 1) at a global scale (GLO), 2) in Europe (EUR) and 3) in North America (NAM)

S7A. Emissions of NOx, NH3 and SO2 for the AQMEII3/HTAP and ED/TFMM communities **B.** Differences between the emissions of NOX, NH3 and SO2 of the AQMEII3/HTAP and ED/TFMM communities

S1.1 Annual deposition of WNH4_N

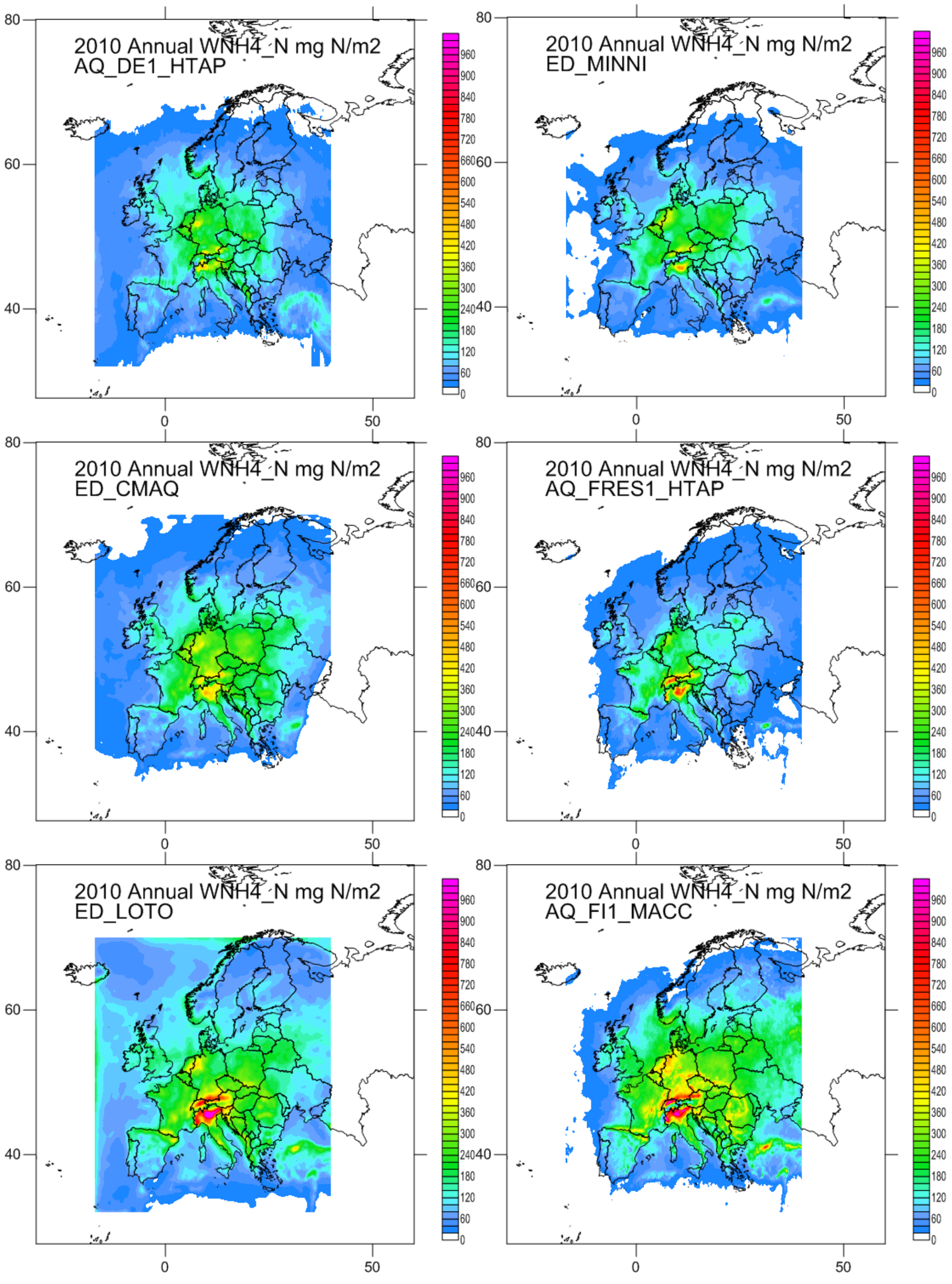

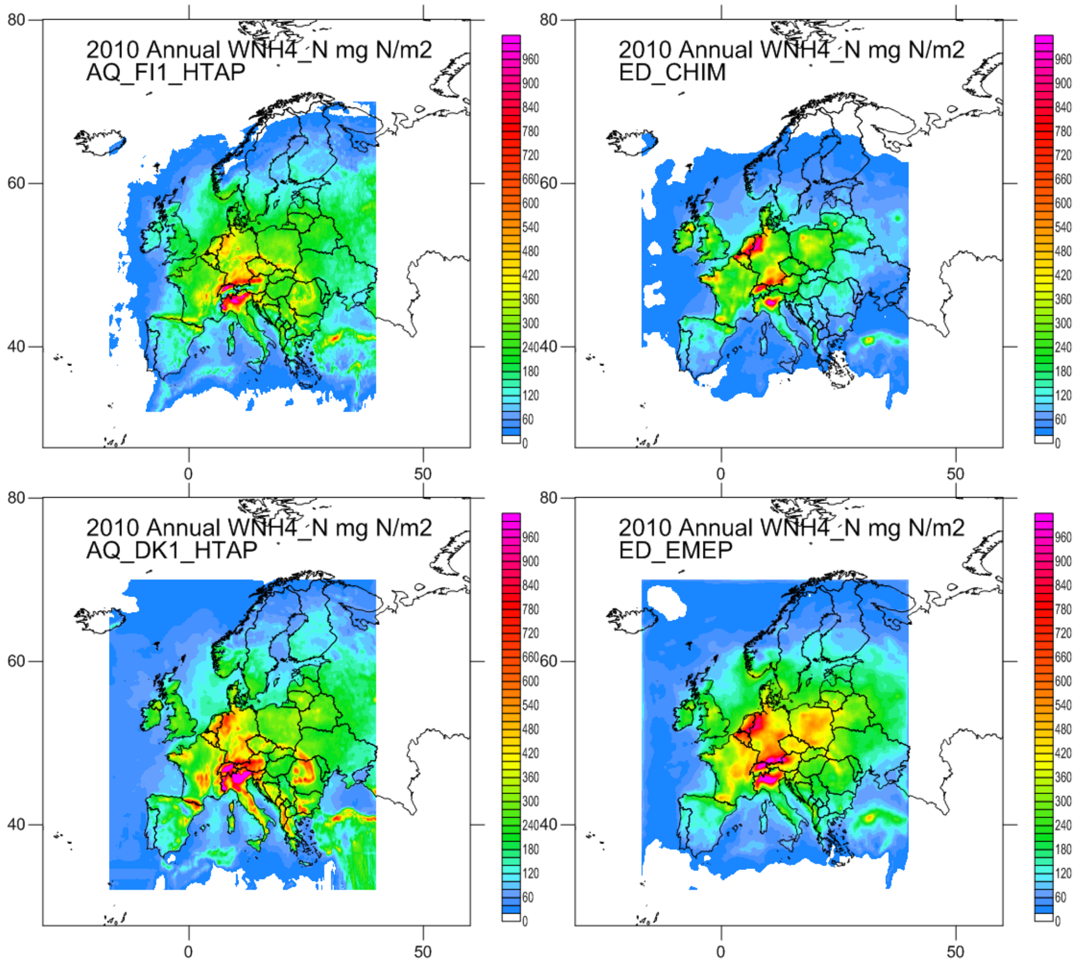
2010 Annual WNH4_N mg N/m2
AQ_FI1_HTAP
2010 Annual WNH4_N mg N/m2
ED_CHIM
2010 Annual WNH4_N mg N/m2
AQ_DK1_HTAP
2010 Annual WNH4_N mg N/m2
ED_EMEP
0 60 120 180 240 300 360 420 480 540 600 660 720 780 840 900 960
80 60 40
0 50

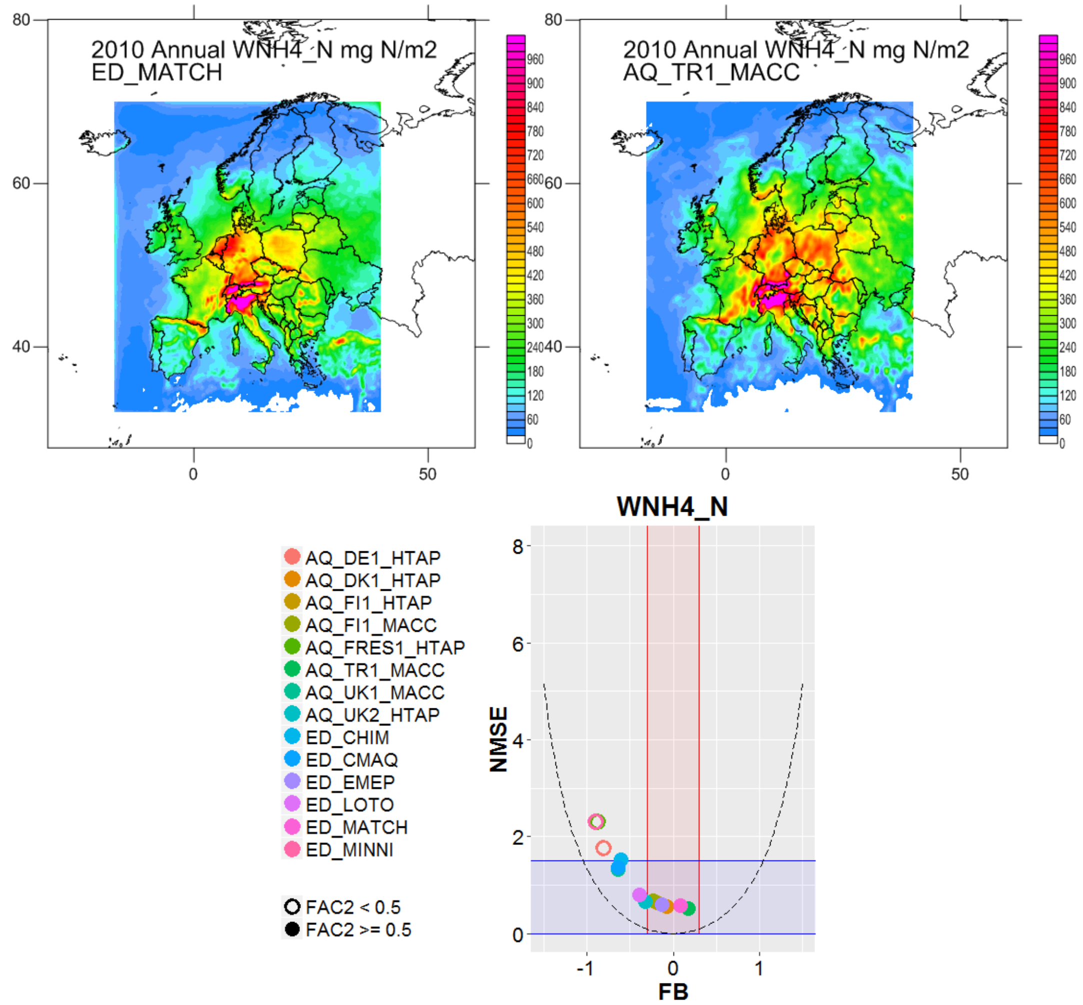

2010 Annual WNH4_N mg N/m2
ED_MATCH
2010 Annual WNH4_N mg N/m2
AQ_TR1_MACC
80
60
40
0
50
960
900
840
780
720
660
600
540
480
420
360
300
240
180
120
60
0
WNH4_N
AQ_DE1_HTAP
AQ_DK1_HTAP
AQ_FI1_HTAP
AQ_FI1_MACC
AQ_FRES1_HTAP
AQ_TR1_MACC
AQ_UK1_MACC
AQ_UK2_HTAP
ED_CHIM
ED_CMAQ
ED_EMEP
ED_LOTO
ED_MATCH
ED_MINNI
FAC2 < 0.5
FAC2 >= 0.5
NMSE
FB
8
6
4
2
0
-1
0
1

S1.2 Annual deposition of WNO3_N

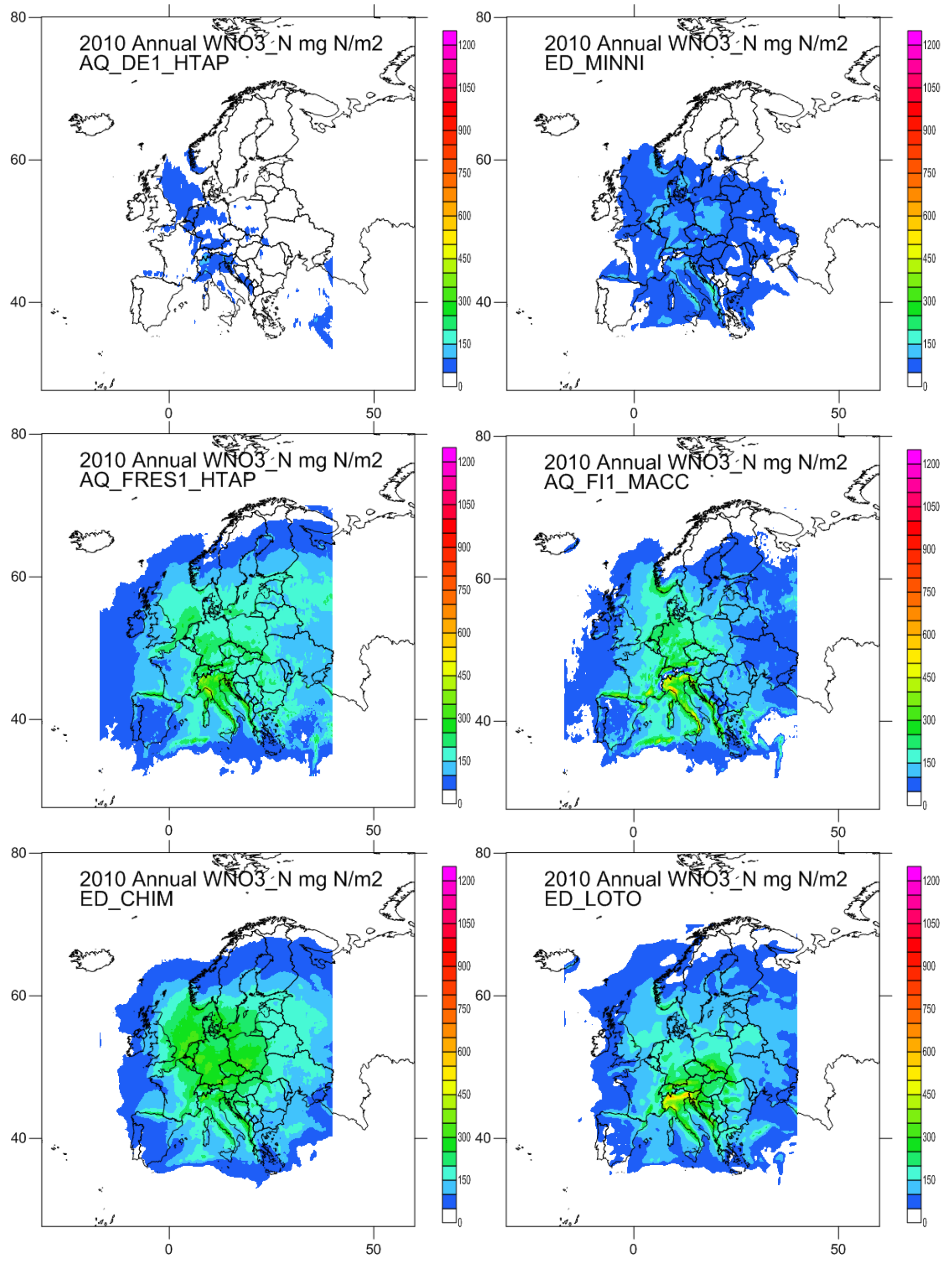

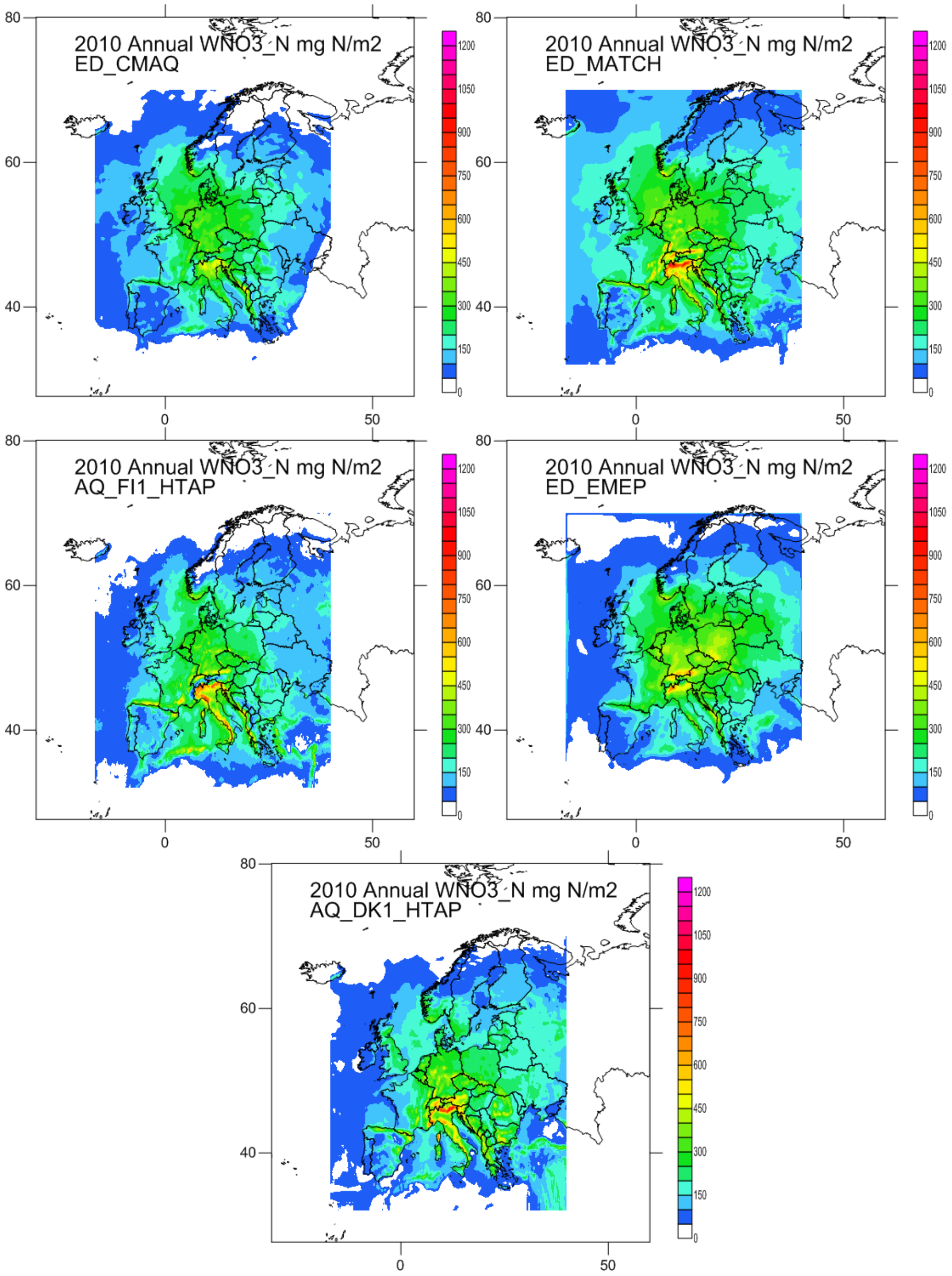
2010 Annual WNO3_N mg N/m2
ED_CMAQ
2010 Annual WNO3_N mg N/m2
ED_MATCH
2010 Annual WNO3_N mg N/m2
AQ_FI1_HTAP
2010 Annual WNO3_N mg N/m2
ED_EMEP
2010 Annual WNO3_N mg N/m2
AQ_DK1_HTAP
80
60
40
0
50
1200
1050
900
750
600
450
300
150
0

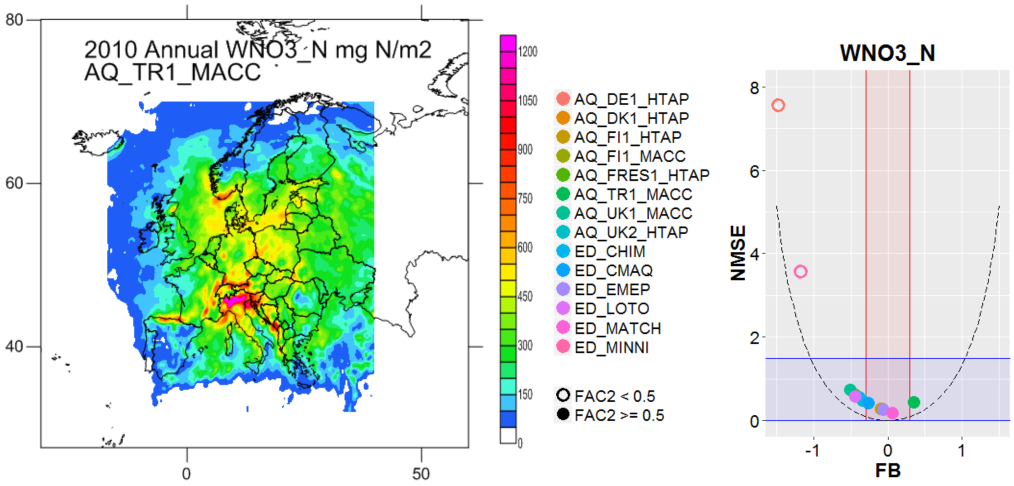
2010 Annual WNO3_N mg N/m2
AQ_TR1_MACC
80
60
40
0
50
1200
1050
900
750
600
450
300
150
0
AQ_DE1_HTAP
AQ_DK1_HTAP
AQ_FI1_HTAP
AQ_FI1_MACC
AQ_FRES1_HTAP
AQ_TR1_MACC
AQ_UK1_MACC
AQ_UK2_HTAP
ED_CHIM
ED_CMAQ
ED_EMEP
ED_LOTO
ED_MATCH
ED_MINNI
FAC2 < 0.5
FAC2 >= 0.5
WNO3_N
NMSE
8
6
4
2
0
-1
0
1
FB

S1.3 Annual deposition of total wet N (WNH4_N + WNO3_N)

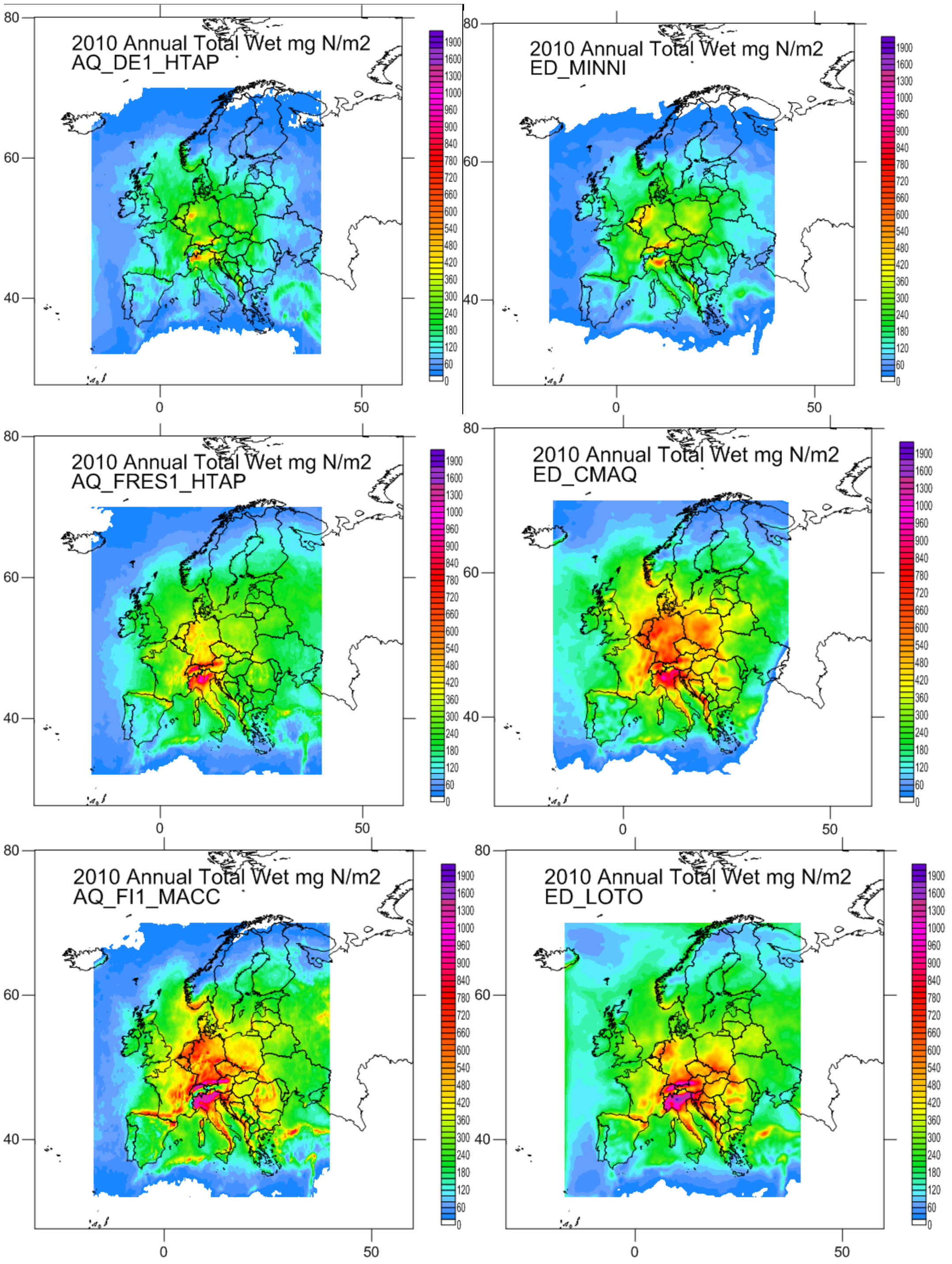

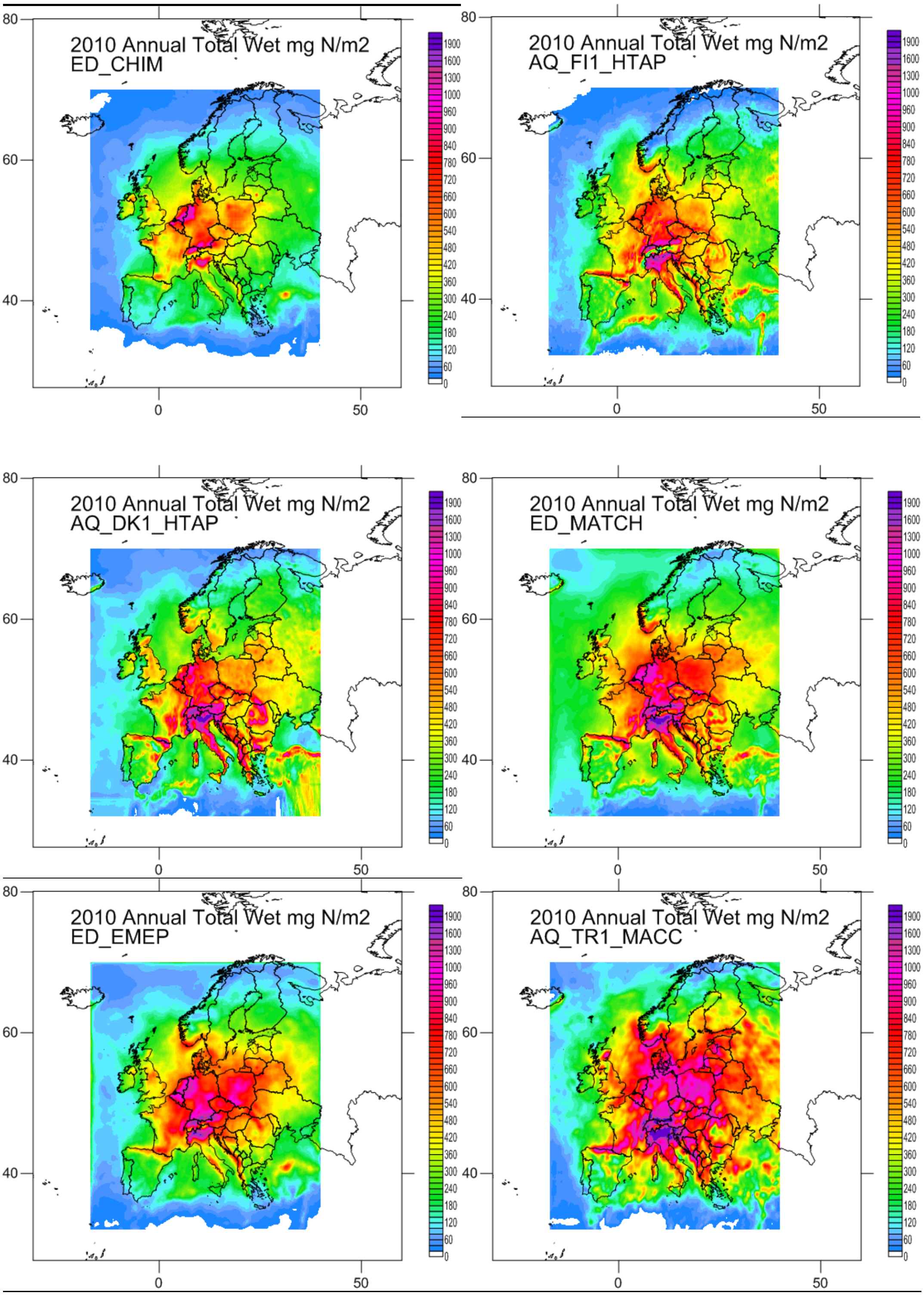

2010 Annual Total Wet mg N/m2
ED_CHIM
2010 Annual Total Wet mg N/m2
AQ_FI1_HTAP
2010 Annual Total Wet mg N/m2
AQ_DK1_HTAP
2010 Annual Total Wet mg N/m2
ED_MATCH
2010 Annual Total Wet mg N/m2
ED_EMEP
2010 Annual Total Wet mg N/m2
AQ_TR1_MACC
1900 1600 1300 1000 960 900 840 780 720 660 600 540 480 420 360 300 240 180 120 60 0
80 60 40
0 50

S1.3 Annual deposition of WSO4_S

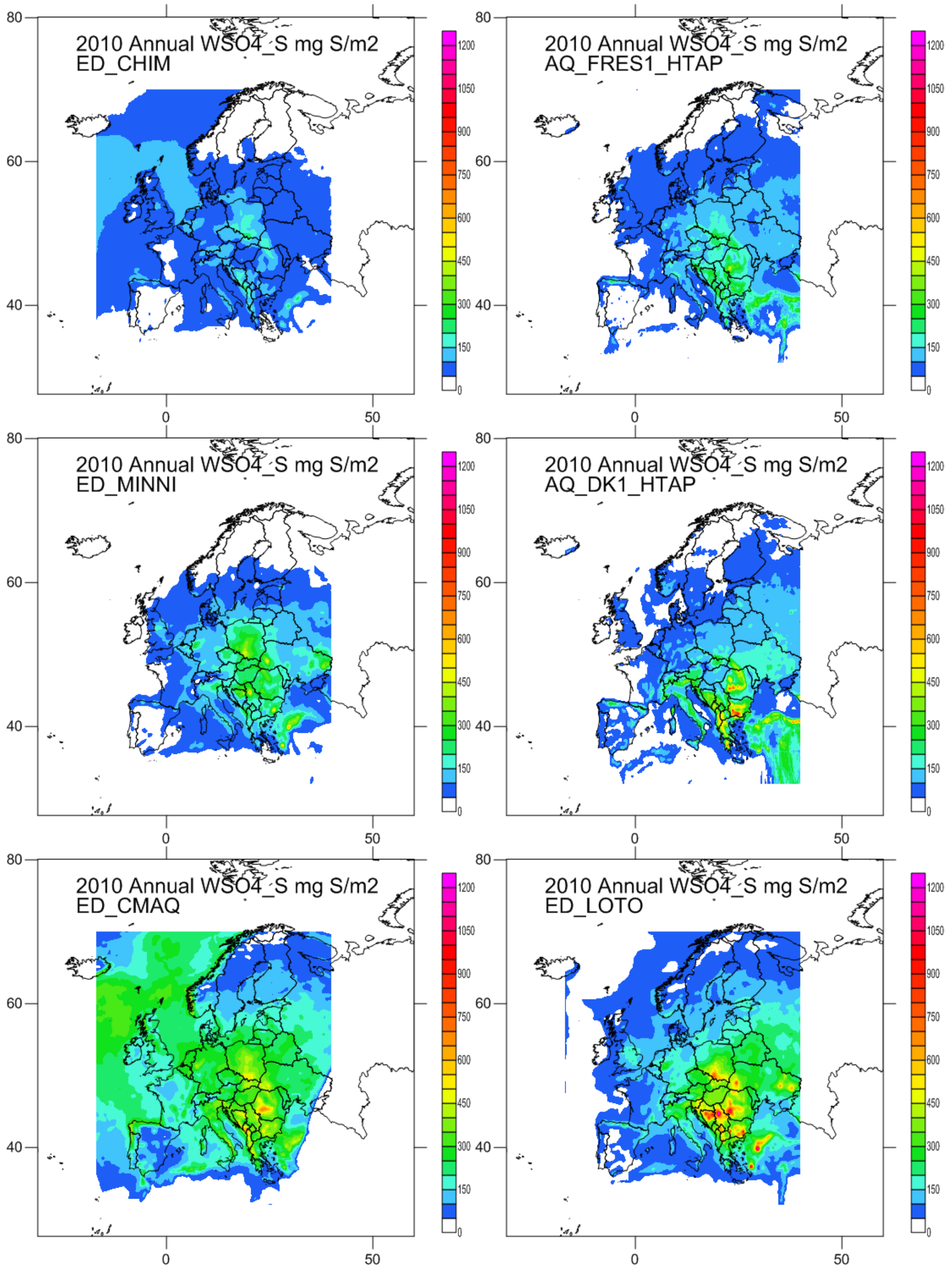

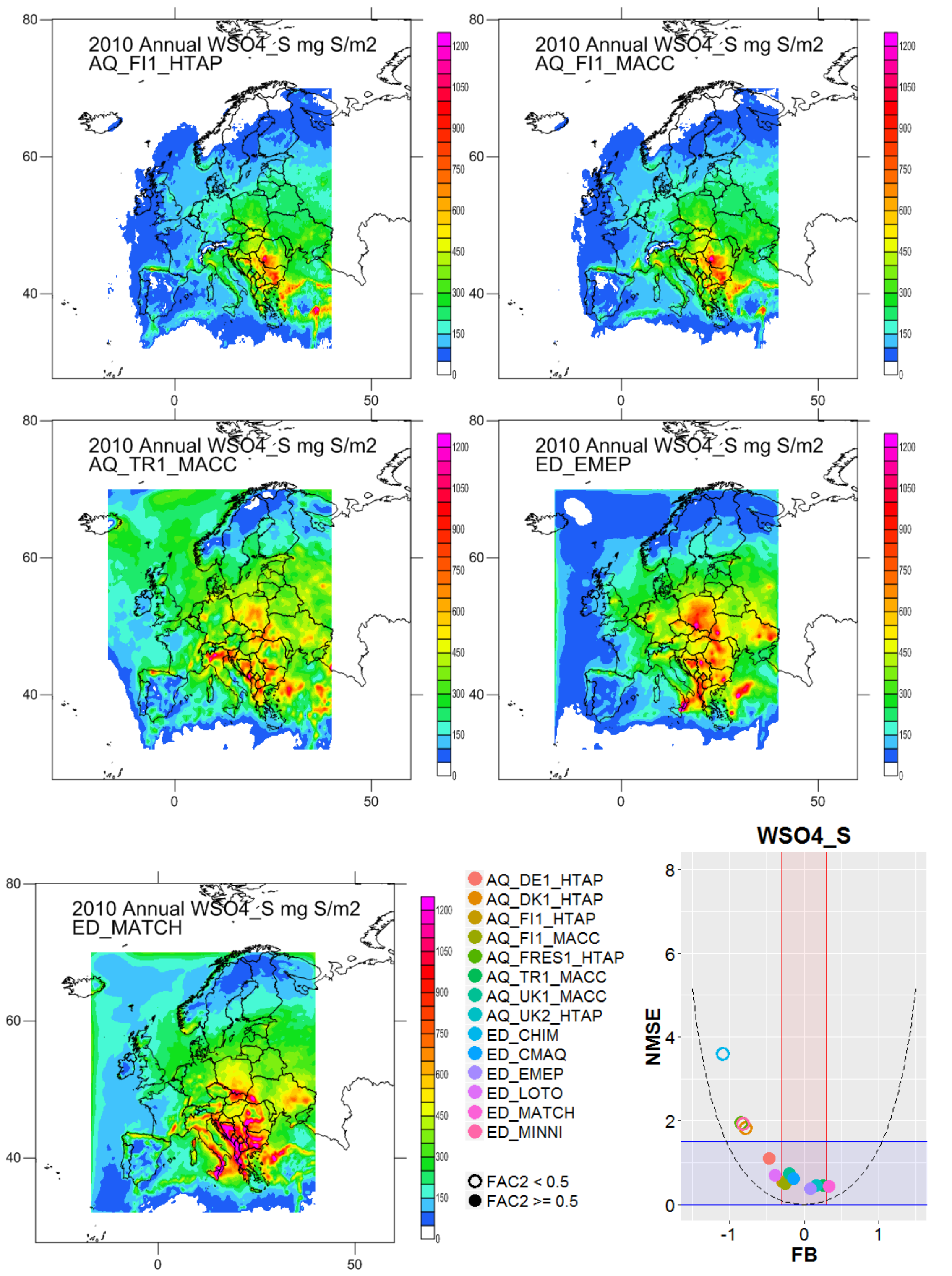

2010 Annual WSO4_S mg S/m2
AQ_FI1_HTAP
2010 Annual WSO4_S mg S/m2
AQ_FI1_MACC
2010 Annual WSO4_S mg S/m2
AQ_TR1_MACC
2010 Annual WSO4_S mg S/m2
ED_EMEP
2010 Annual WSO4_S mg S/m2
ED_MATCH
WSO4_S
NMSE
FB
AQ_DE1_HTAP
AQ_DK1_HTAP
AQ_FI1_HTAP
AQ_FI1_MACC
AQ_FRES1_HTAP
AQ_TR1_MACC
AQ_UK1_MACC
AQ_UK2_HTAP
ED_CHIM
ED_CMAQ
ED_EMEP
ED_LOTO
ED_MATCH
ED_MINNI
FAC2 < 0.5
FAC2 >= 0.5

S2. MAPS OF DRY DEPOSITION FOR DIFFERENT COMPOUNDS

S2.1 Annual deposition of DNH4_N

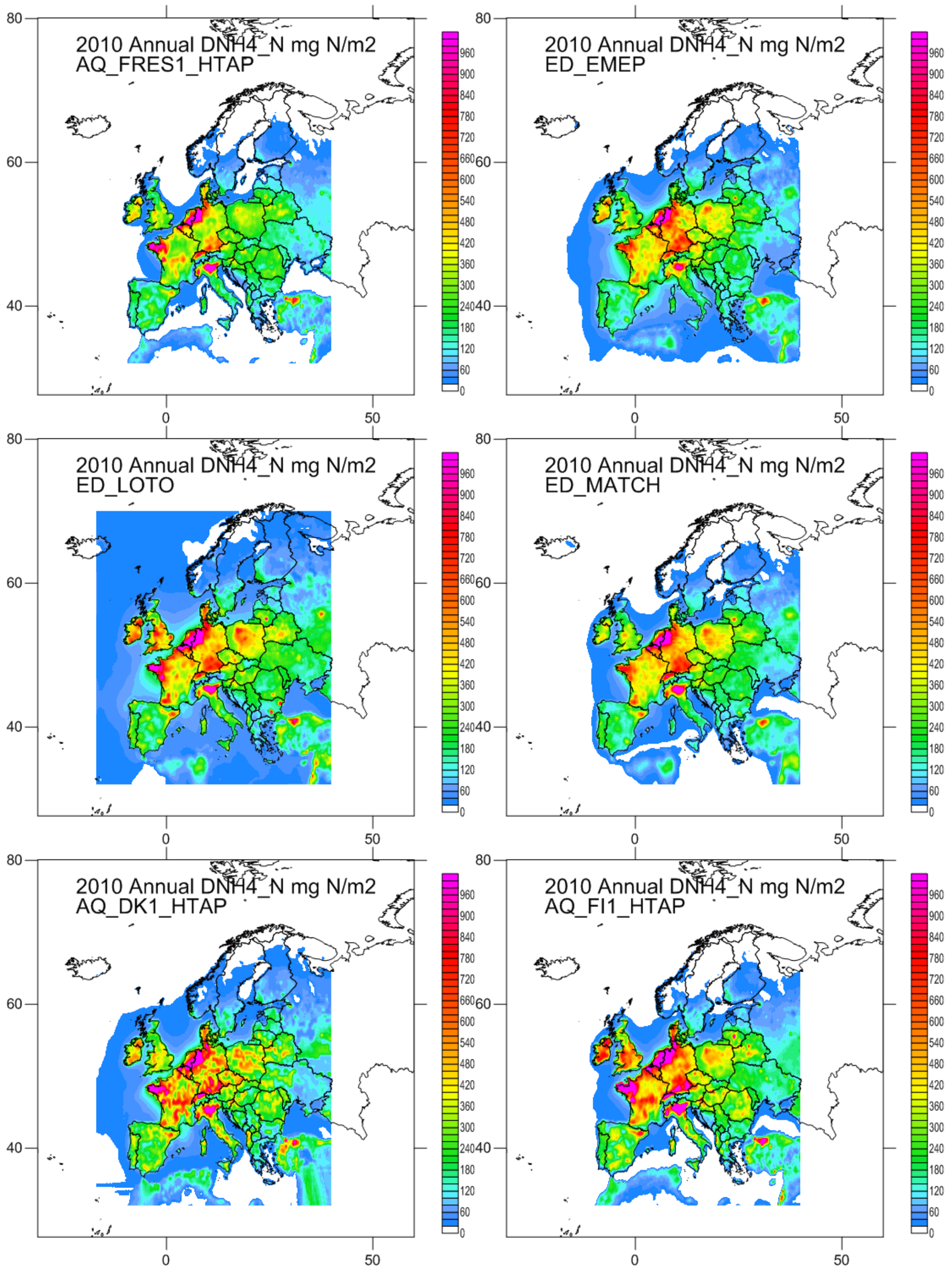

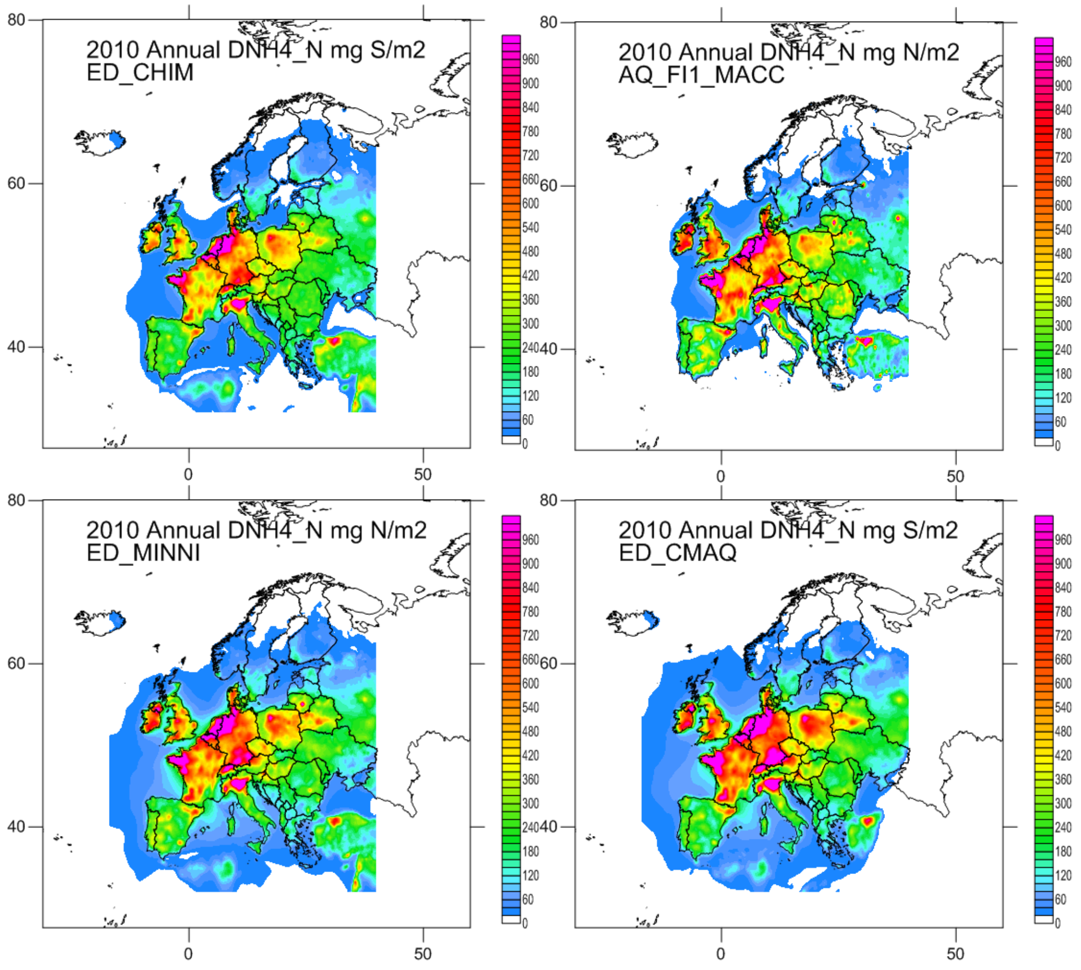
2010 Annual DNH4_N mg S/m2
ED_CHIM
2010 Annual DNH4_N mg N/m2
AQ_FI1_MACC
2010 Annual DNH4_N mg N/m2
ED_MINNI
2010 Annual DNH4_N mg S/m2
ED_CMAQ
80
60
40
0
50
960
900
840
780
720
660
600
540
480
420
360
300
240
180
120
60
0

S2.2 Annual deposition of DNO3_N

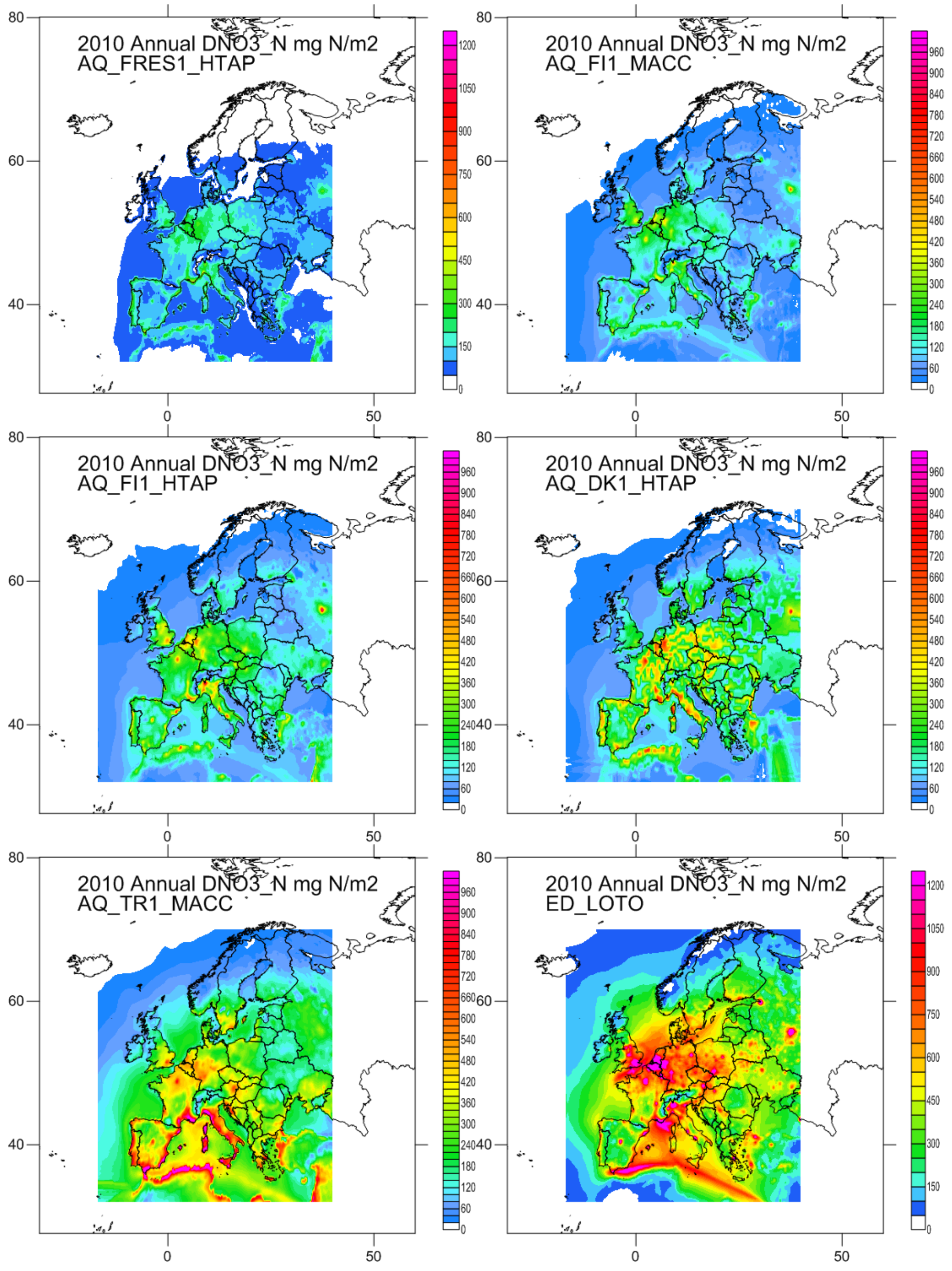

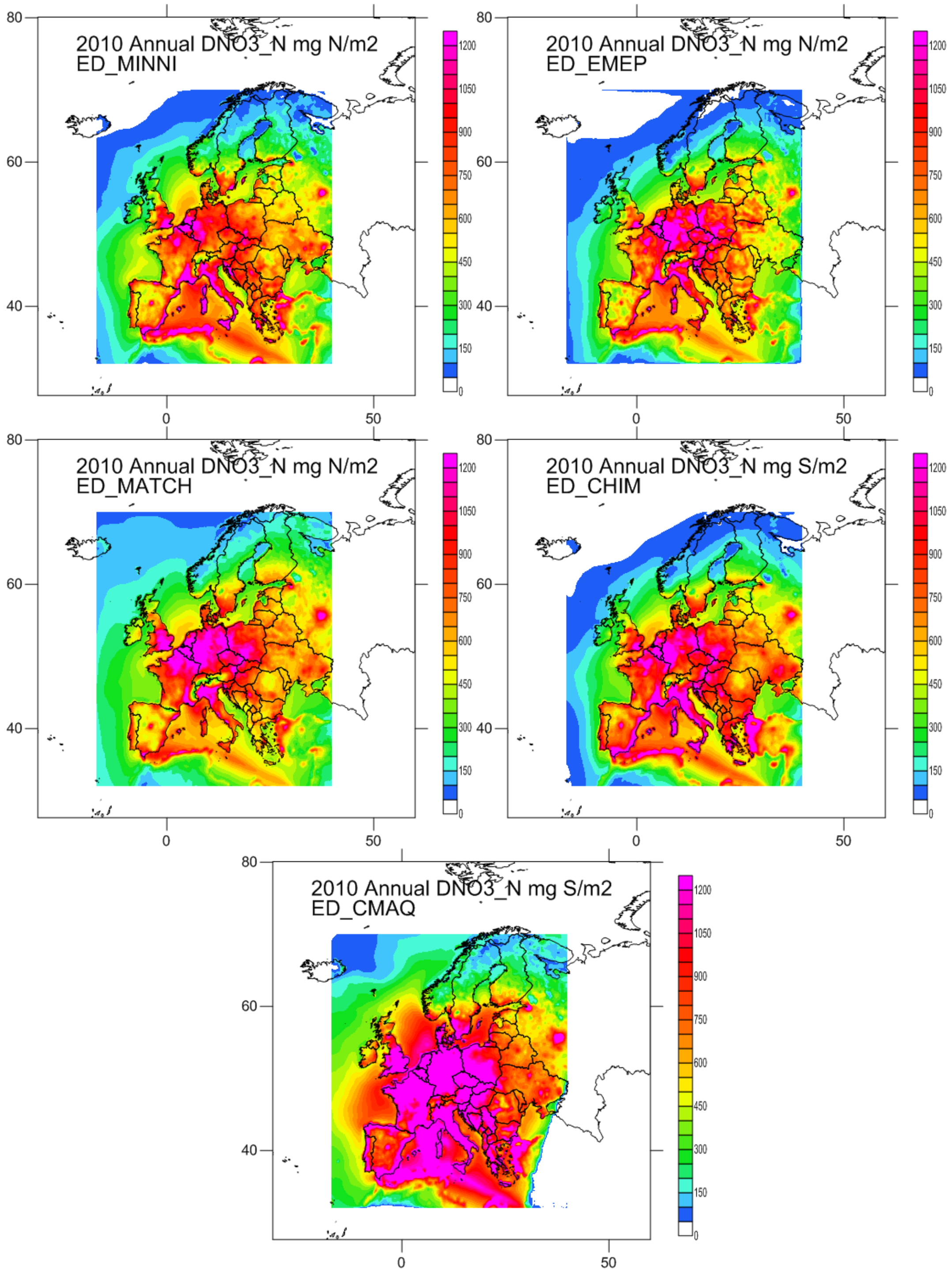

2010 Annual DNO3_N mg N/m2
ED_MINNI
2010 Annual DNO3_N mg N/m2
ED_EMEP
2010 Annual DNO3_N mg N/m2
ED_MATCH
2010 Annual DNO3_N mg S/m2
ED_CHIM
2010 Annual DNO3_N mg S/m2
ED_CMAQ
80
60
40
0
50
1200
1050
900
750
600
450
300
150
0

S2.3 Annual dry deposition of the gas and particle components contributing to DNO3_N

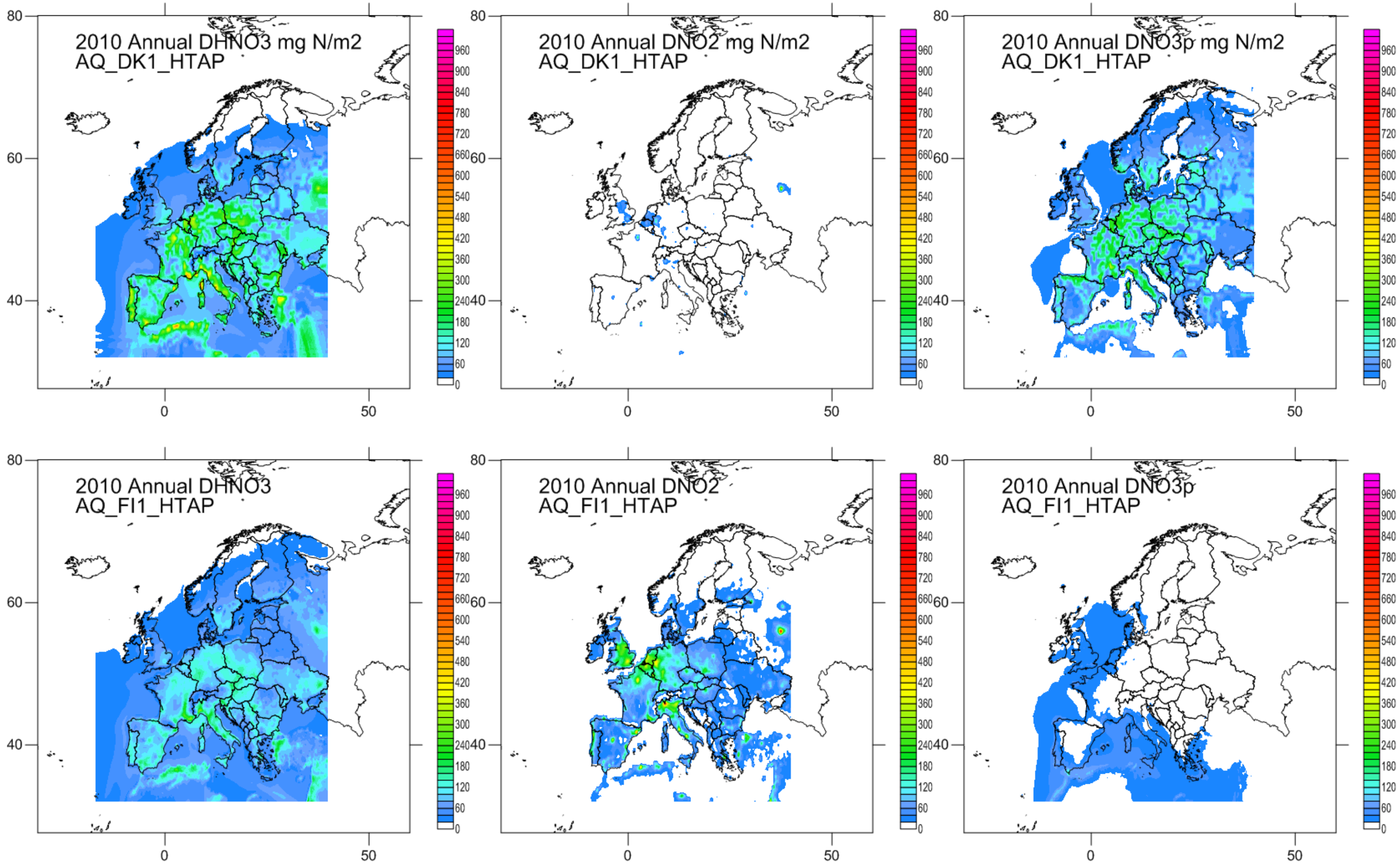

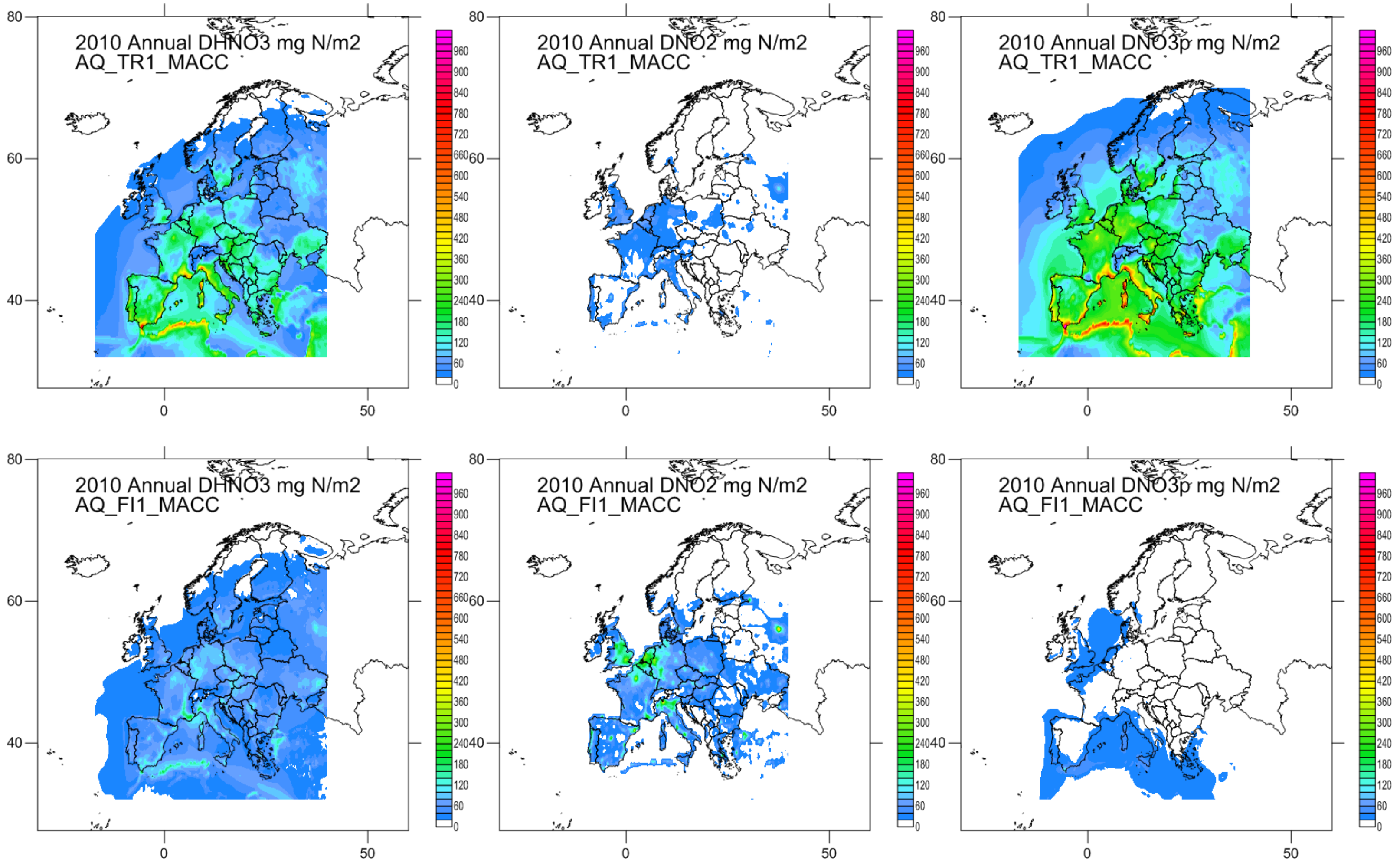
2010 Annual DHNO3 mg N/m2
AQ_TR1_MACC
2010 Annual DNO2 mg N/m2
AQ_TR1_MACC
2010 Annual DNO3p mg N/m2
AQ_TR1_MACC
2010 Annual DHNO3 mg N/m2
AQ_FI1_MACC
2010 Annual DNO2 mg N/m2
AQ_FI1_MACC
2010 Annual DNO3p mg N/m2
AQ_FI1_MACC
80
60
40
0
50
0
60
120
180
240
300
360
420
480
540
600
660
720
780
840
900
960

S2.4 Annual deposition of total dry N (DNH4_N + DNO3_N)

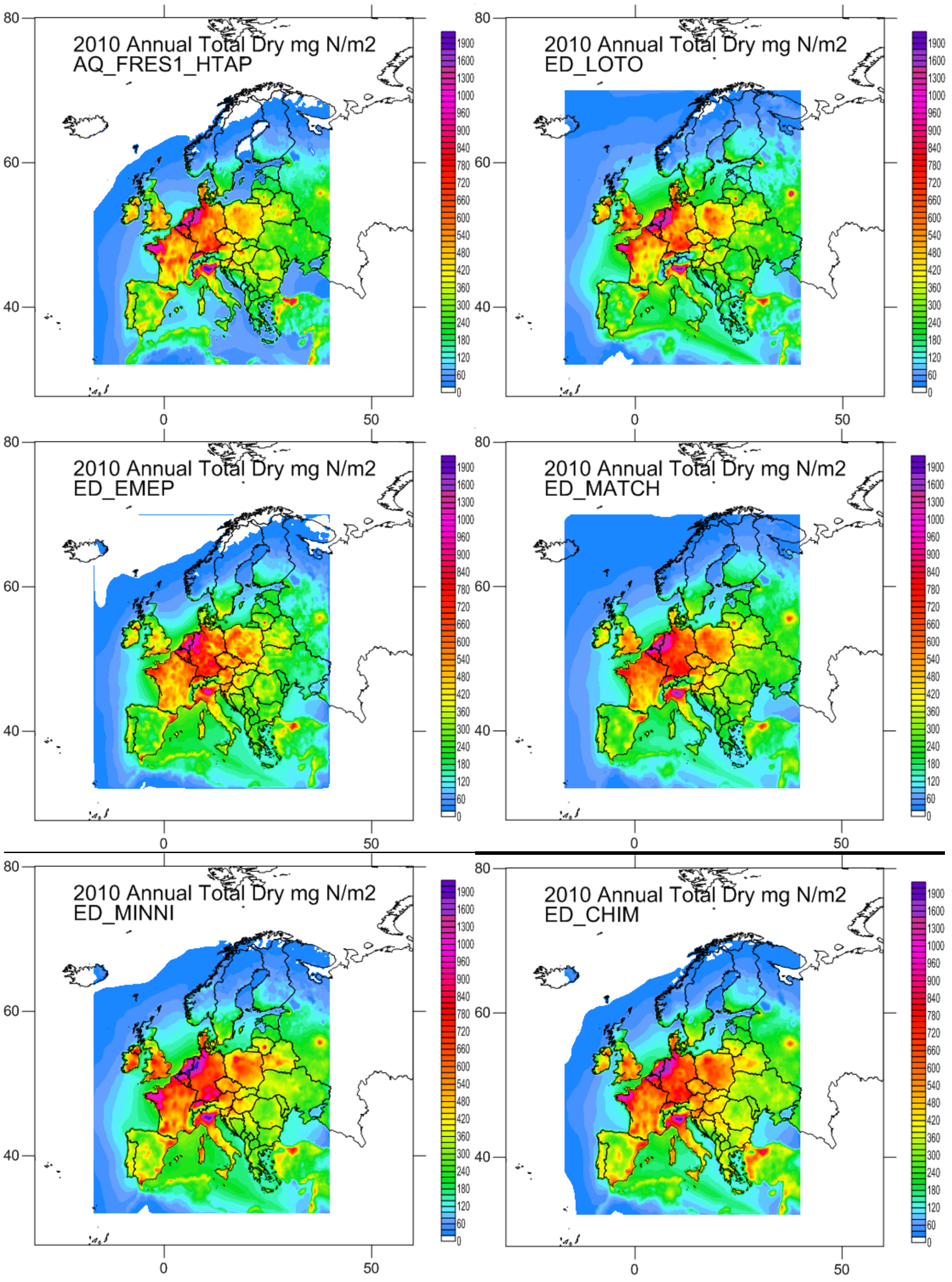

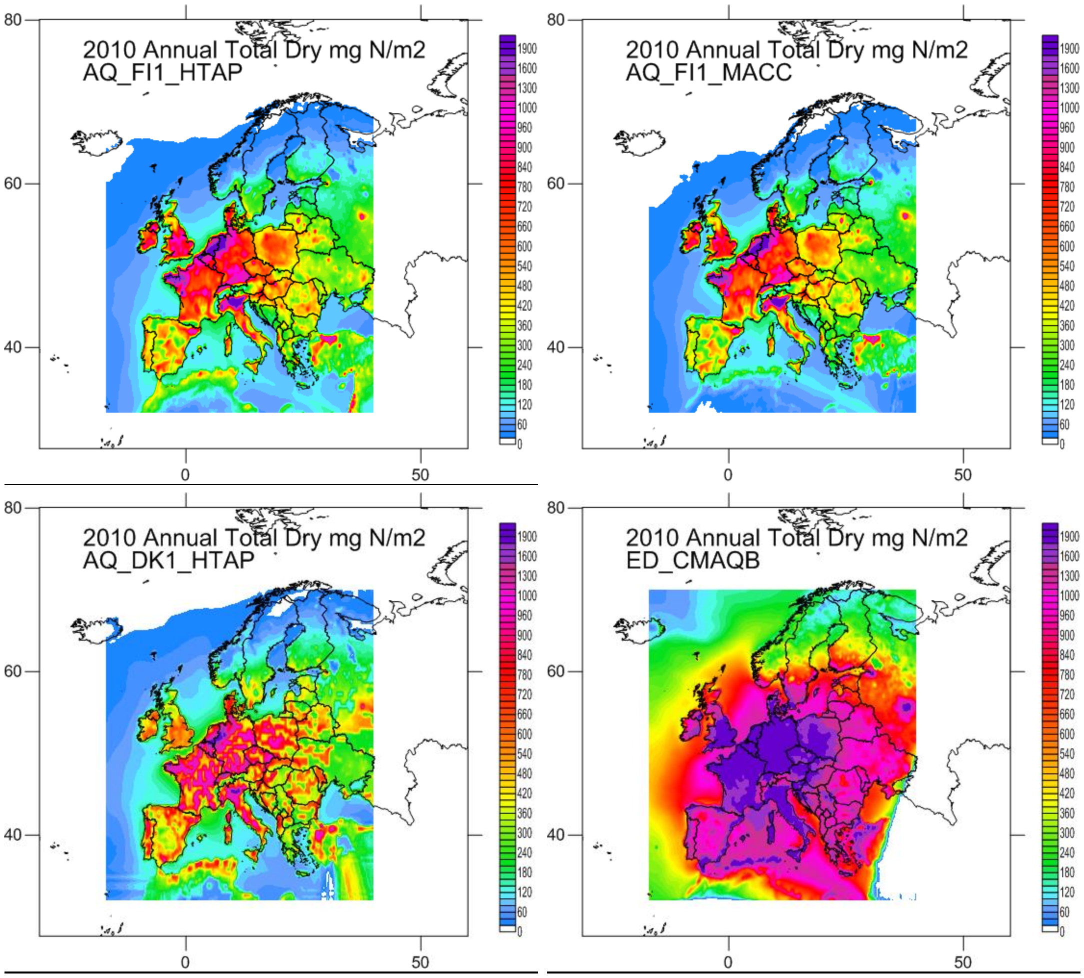
2010 Annual Total Dry mg N/m2
AQ_FI1_HTAP
2010 Annual Total Dry mg N/m2
AQ_FI1_MACC
2010 Annual Total Dry mg N/m2
AQ_DK1_HTAP
2010 Annual Total Dry mg N/m2
ED_CMAQB
80
60
40
0
50
1900
1600
1300
1000
960
900
840
780
720
660
600
540
480
420
360
300
240
180
120
60
0

S2.5 Annual deposition of DSO4_S

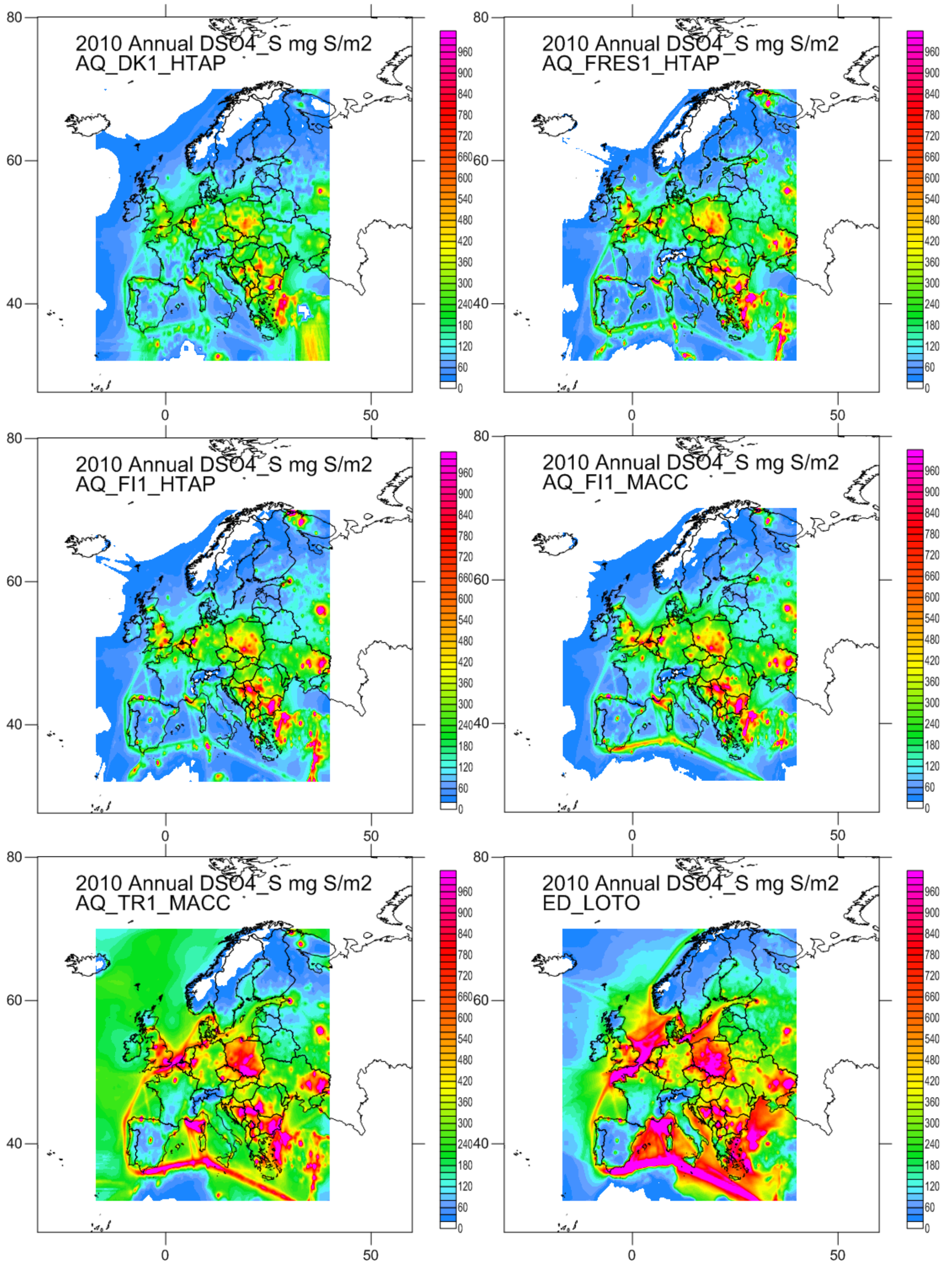

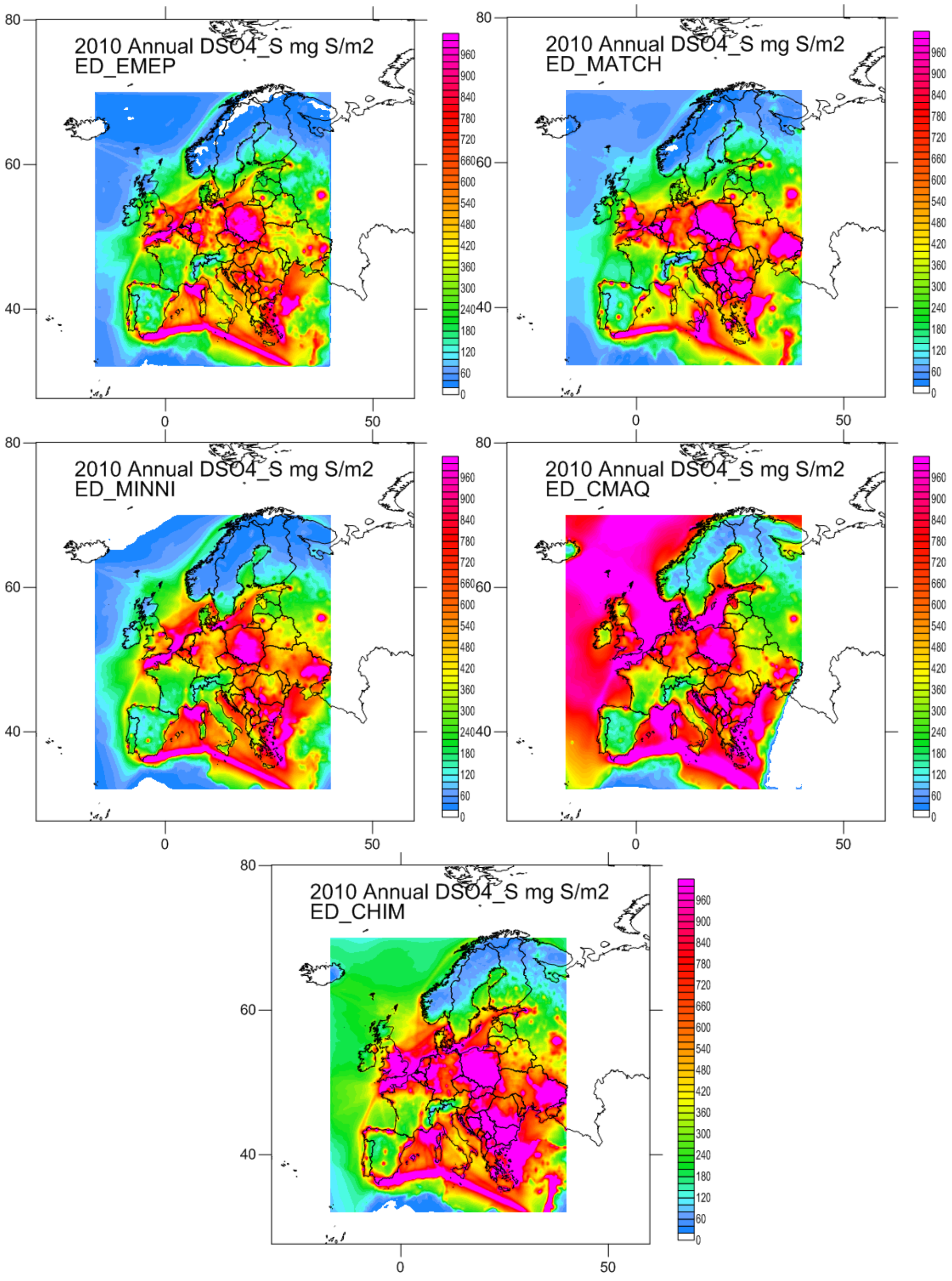
2010 Annual DSO4_S mg S/m2
ED_EMEP
2010 Annual DSO4_S mg S/m2
ED_MATCH
2010 Annual DSO4_S mg S/m2
ED_MINNI
2010 Annual DSO4_S mg S/m2
ED_CMAQ
2010 Annual DSO4_S mg S/m2
ED_CHIM
80
60
40
0
50
960
900
840
780
720
660
600
540
480
420
360
300
240
180
120
60
0

S3. STATISTICS FOR WET DEPOSITION FOR DIFFERENT COMPOUNDS

S3.1 STATISTICS FROM MONTHLY VALUES (FB, NMSE and FAC2)

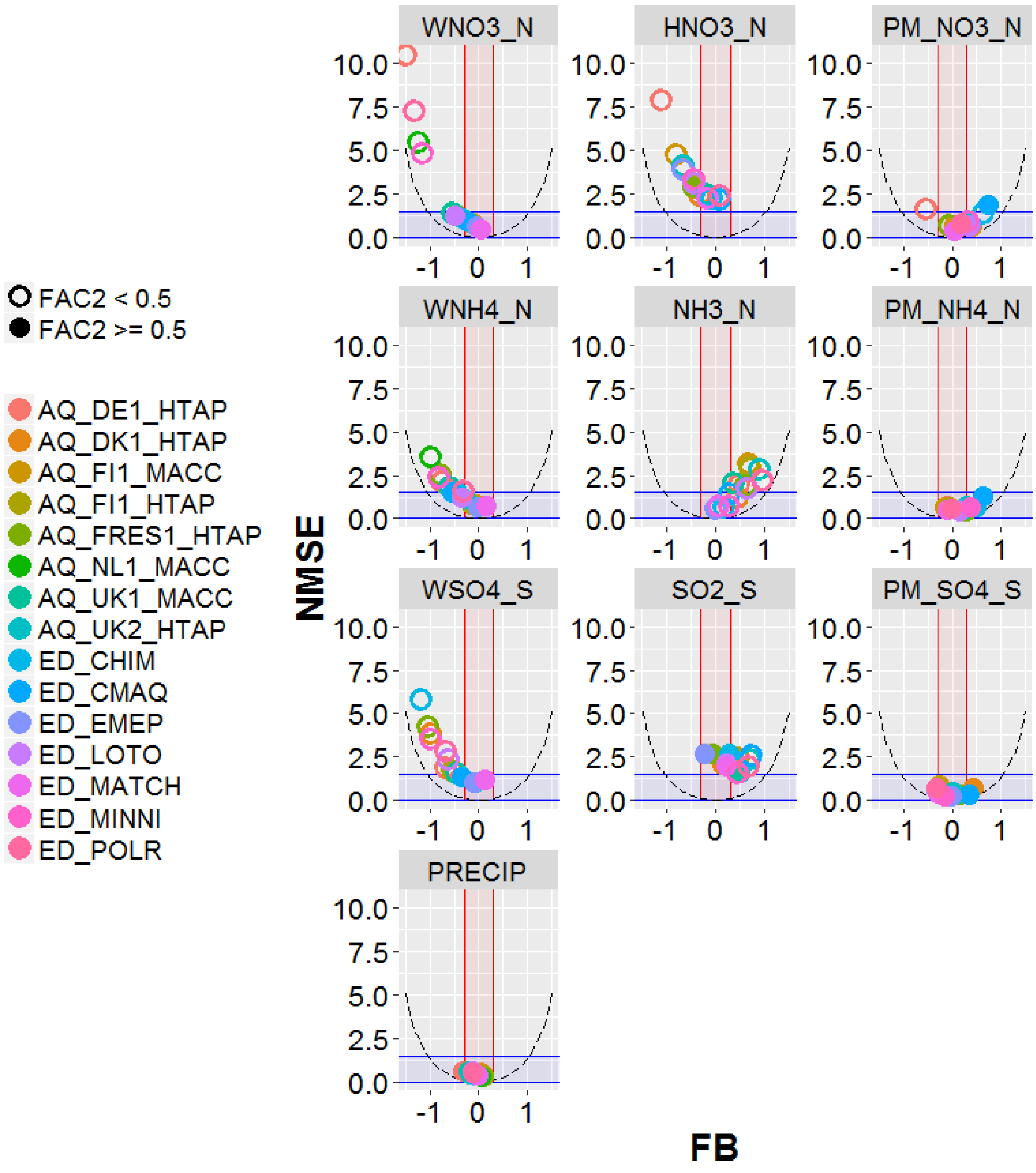

S3.2 STATISTICS BY MONTH (FB, NMSE and FAC2) FOR OXIDISED-N SPECIES

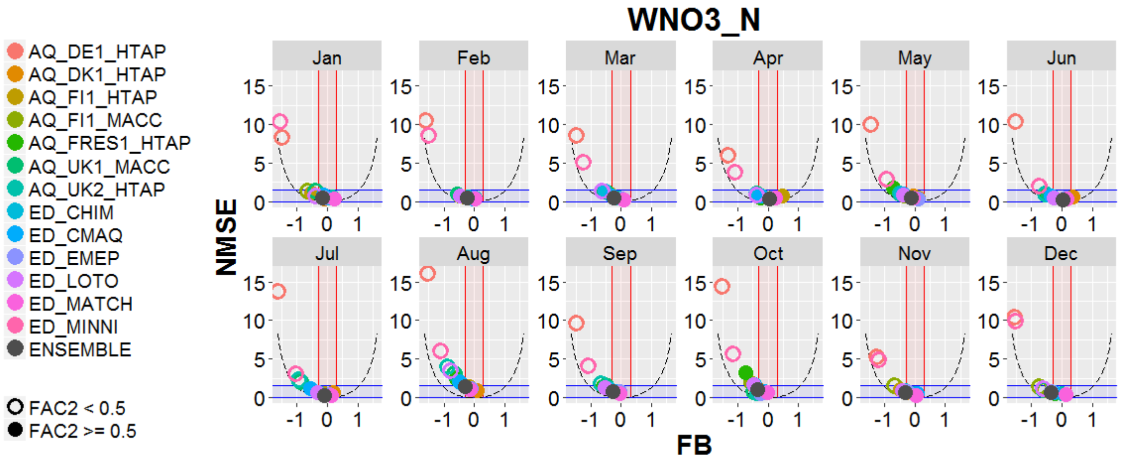

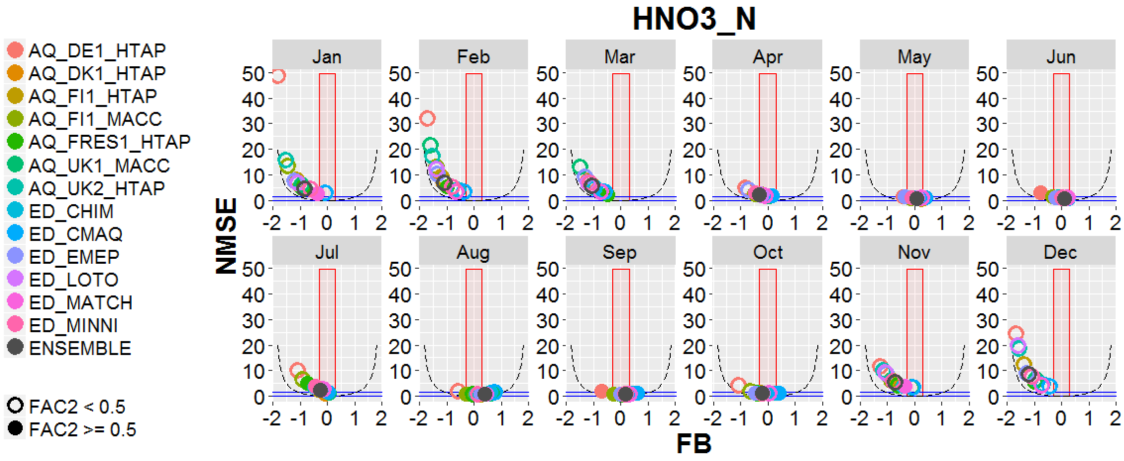

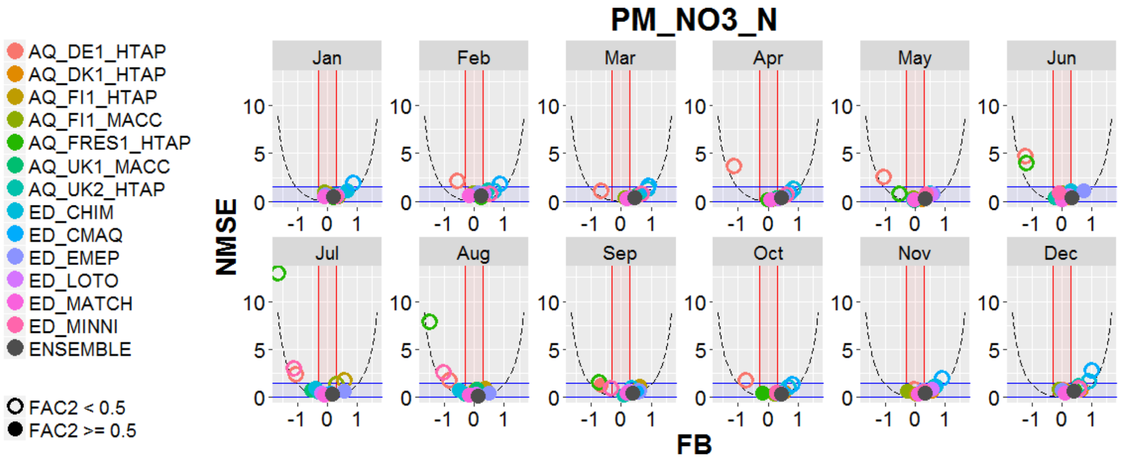

S3.3 STATISTICS BY MONTH (FB, NMSE and FAC2) FOR REDUCED-N SPECIES

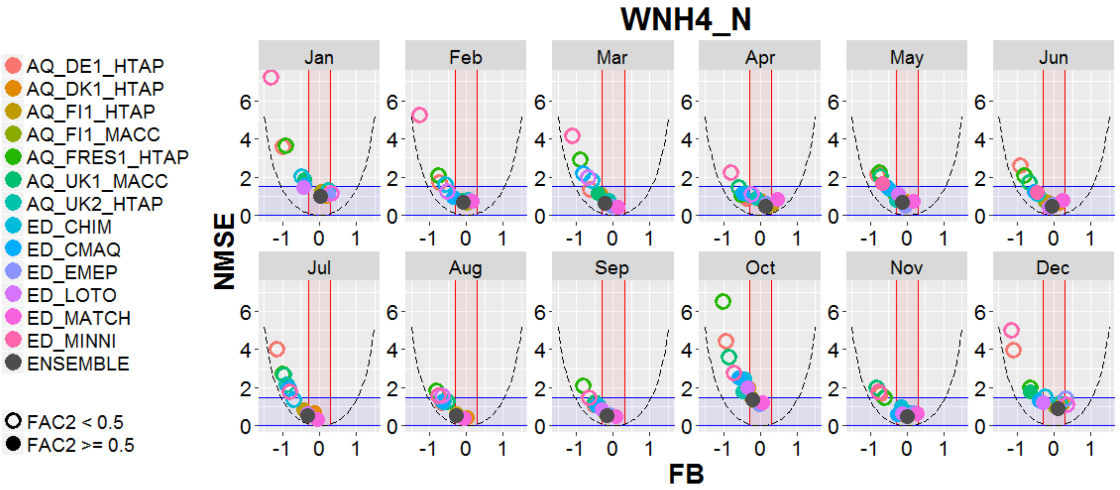

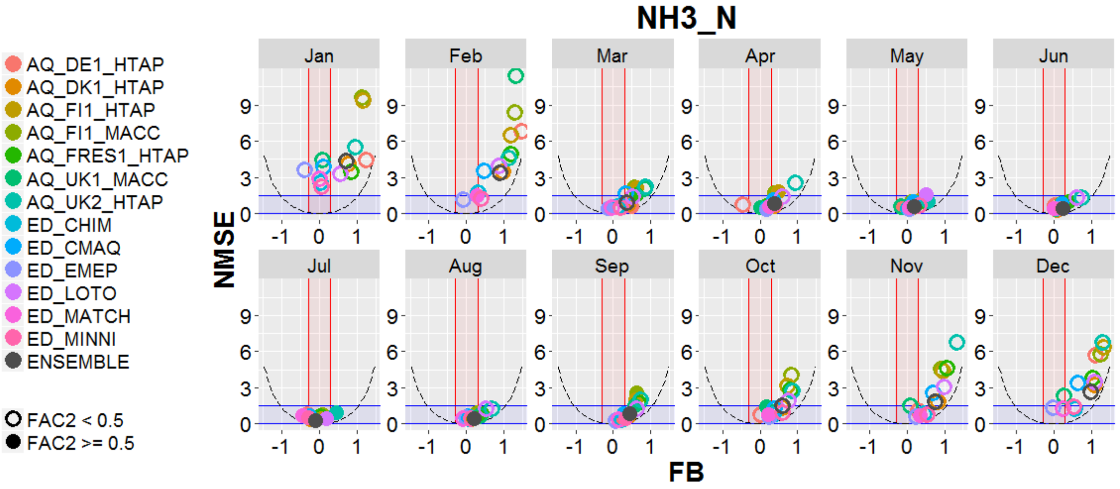

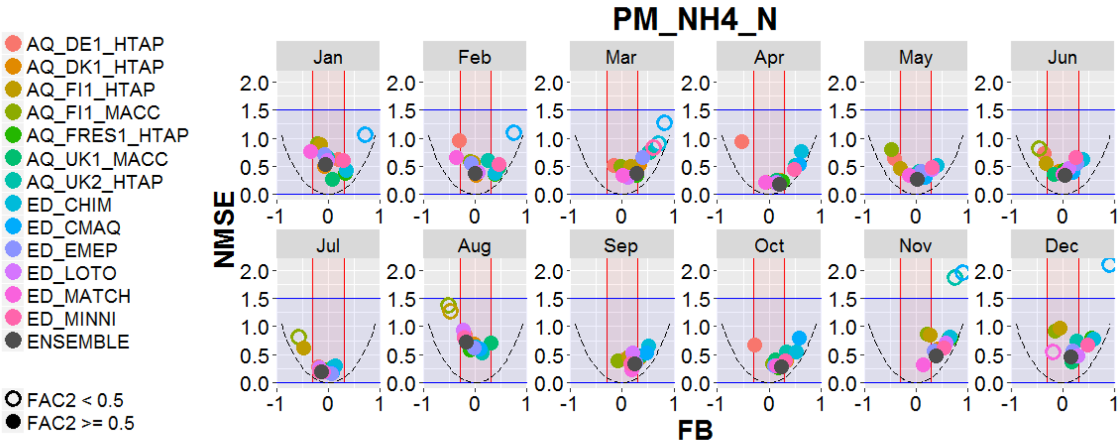

S3.4 Statistics by month (FB, NMSE and FAC2) for S species

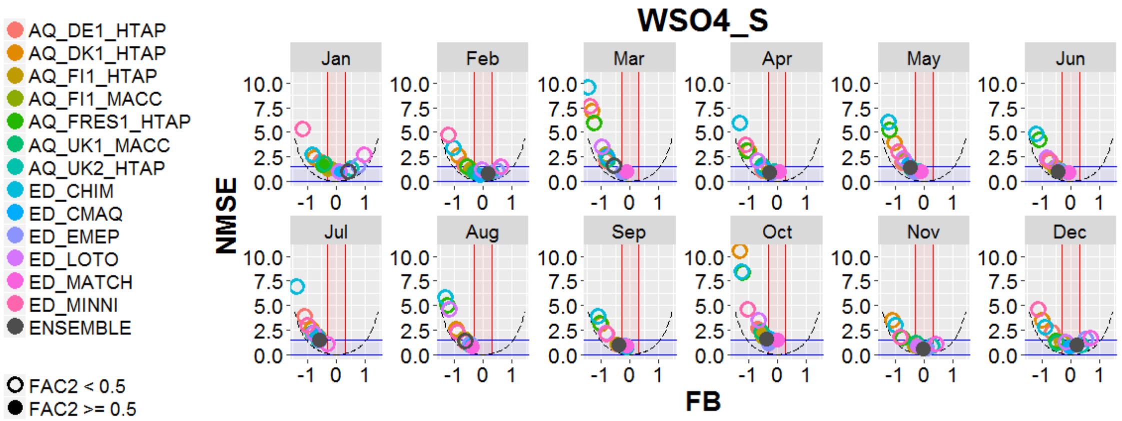

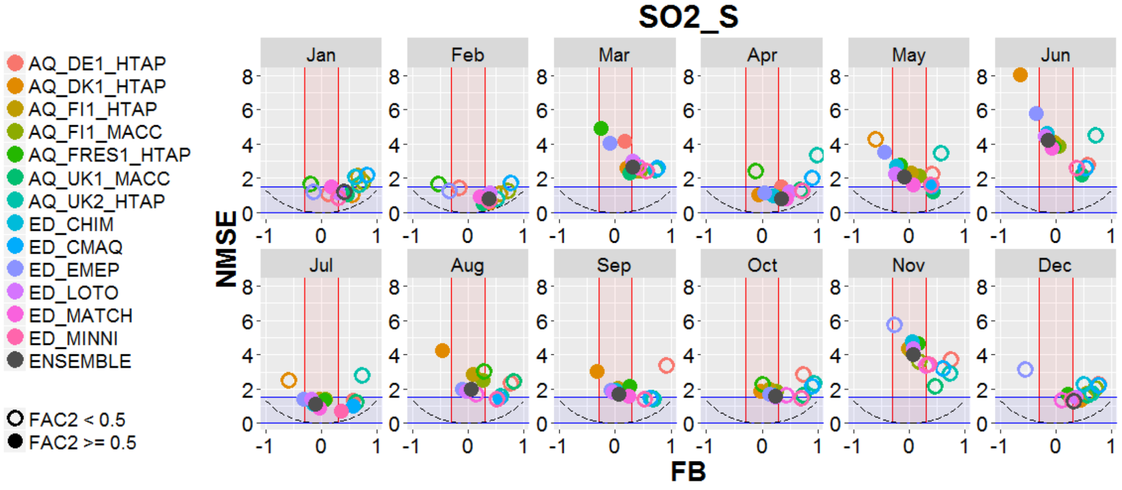

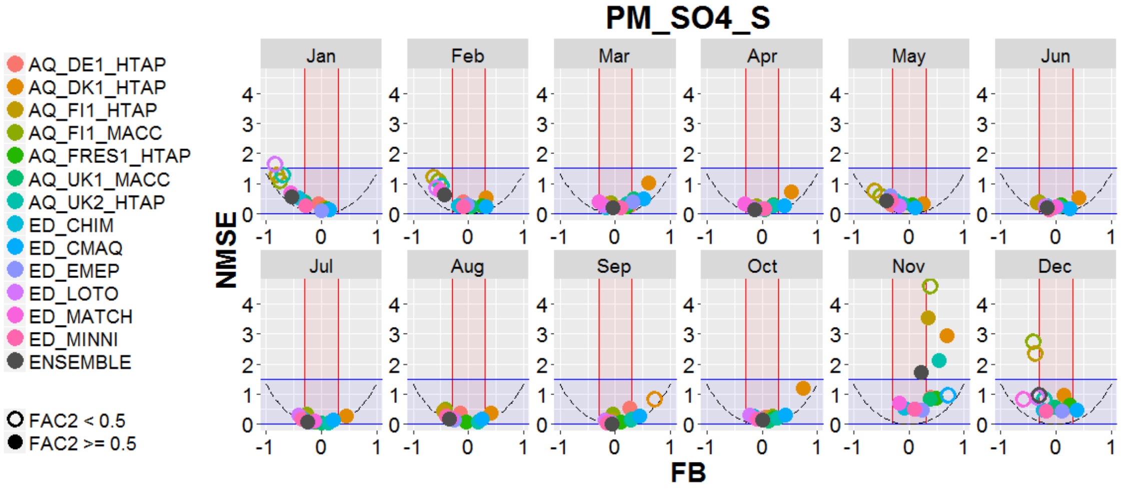

S3.5 Statistics by month (FB, NMSE and FAC2) for precipitation

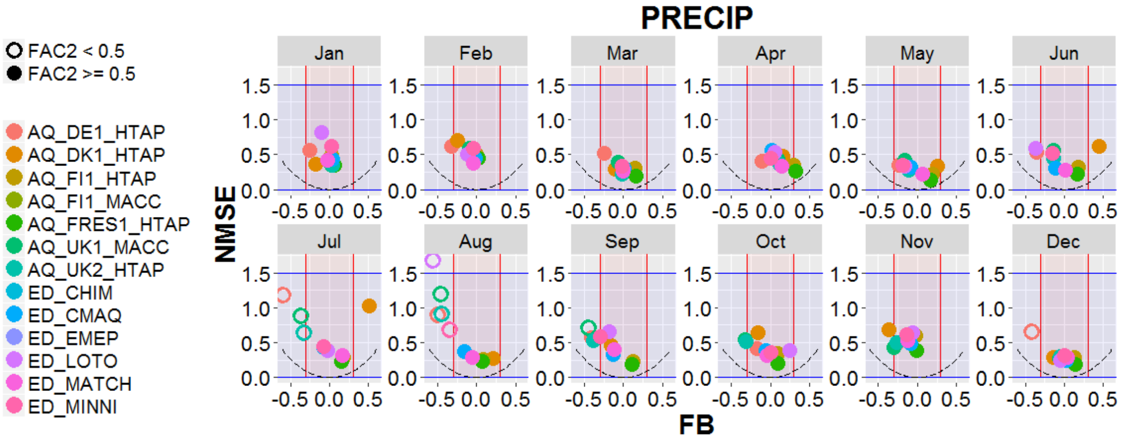

S3.6 . Tables with complete statistics from monthly values (definition in S3.10). All available sites

Variable	Model	n	FAC2	FB	NMSE	MB	MGE	NMB	NMGE	RMSE	r	COE	IOA
WNO3_N	AQ_DE1_HTAP	667	0.05	-1.50	10.50	-19.77	19.86	-0.86	0.86	28.43	0.54	-0.38	0.31
WNO3_N	AQ_DK1_HTAP	682	0.67	-0.10	0.78	-2.25	11.74	-0.10	0.51	19.10	0.54	0.18	0.59
WNO3_N	AQ_FI1_MACC	694	0.53	-0.47	1.26	-8.52	12.15	-0.38	0.54	20.01	0.55	0.15	0.57
WNO3_N	AQ_FI1_HTAP	694	0.63	-0.13	0.80	-2.79	11.65	-0.12	0.52	18.87	0.56	0.18	0.59
WNO3_N	AQ_FRES1_HTAP	694	0.64	-0.46	1.31	-8.38	11.53	-0.37	0.51	20.47	0.53	0.19	0.59
WNO3_N	AQ_UK1_MACC	694	0.47	-0.54	1.42	-9.58	12.66	-0.42	0.56	20.42	0.56	0.11	0.56
WNO3_N	AQ_UK2_HTAP	680	0.59	-0.32	1.06	-6.37	12.14	-0.28	0.53	20.00	0.52	0.15	0.58
WNO3_N	ED_CHIM	682	0.70	-0.36	1.07	-6.93	11.11	-0.30	0.49	19.73	0.54	0.22	0.61
WNO3_N	ED_CMAQ	682	0.69	-0.25	0.91	-5.01	10.97	-0.22	0.48	19.24	0.53	0.23	0.62
WNO3_N	ED_EMEP	682	0.68	-0.05	0.64	-1.08	11.41	-0.05	0.50	17.82	0.60	0.20	0.60
WNO3_N	ED_LOTO	682	0.54	-0.48	1.26	-8.83	12.20	-0.39	0.53	20.09	0.56	0.15	0.57
WNO3_N	ED_MATCH	682	0.75	0.05	0.49	1.21	10.20	0.05	0.45	16.41	0.67	0.29	0.64
WNO3_N	ED_MINNI	682	0.16	-1.16	4.84	-16.79	17.34	-0.74	0.76	25.85	0.48	-0.21	0.39
WNO3_N	ENSEMBLE	682	0.79	-0.20	0.66	-4.18	9.44	-0.18	0.41	16.81	0.68	0.34	0.67
HNO3_N	AQ_DE1_HTAP	166	0.23	-1.11	7.94	-0.10	0.11	-0.71	0.83	0.20	0.12	0.04	0.52
HNO3_N	AQ_DK1_HTAP	154	0.49	-0.35	2.42	-0.04	0.11	-0.30	0.78	0.18	0.35	0.11	0.56
HNO3_N	AQ_FI1_MACC	166	0.40	-0.80	4.79	-0.08	0.10	-0.57	0.78	0.19	0.19	0.10	0.55
HNO3_N	AQ_FI1_HTAP	166	0.43	-0.41	2.87	-0.05	0.11	-0.34	0.80	0.18	0.22	0.09	0.54
HNO3_N	AQ_FRES1_HTAP	166	0.50	-0.47	2.93	-0.05	0.10	-0.38	0.74	0.18	0.25	0.15	0.58
HNO3_N	AQ_UK1_MACC	154	0.45	-0.21	2.50	-0.03	0.12	-0.19	0.89	0.20	0.20	0.00	0.50
HNO3_N	AQ_UK2_HTAP	154	0.38	-0.67	4.14	-0.07	0.12	-0.50	0.85	0.20	0.14	0.04	0.52
HNO3_N	ED_CHIM	154	0.49	-0.09	2.30	-0.01	0.13	-0.09	0.92	0.20	0.15	-0.04	0.48
HNO3_N	ED_CMAQ	154	0.48	0.09	2.15	0.01	0.13	0.09	0.96	0.21	0.14	-0.09	0.45
HNO3_N	ED_EMEP	154	0.38	-0.67	3.92	-0.07	0.11	-0.50	0.81	0.19	0.22	0.08	0.54
HNO3_N	ED_LOTO	154	0.42	-0.44	3.16	-0.05	0.11	-0.36	0.83	0.20	0.15	0.06	0.53

Variable	Model	n	FAC2	FB	NMSE	MB	MGE	NMB	NMGE	RMSE	r	COE	IOA
HNO3_N	ED_MATCH	154	0.42	-0.16	2.24	-0.02	0.12	-0.15	0.88	0.19	0.15	0.00	0.50
HNO3_N	ED_MINNI	154	0.40	-0.45	3.37	-0.05	0.12	-0.37	0.87	0.20	0.06	0.02	0.51
HNO3_N	ENSEMBLE	154	0.50	-0.40	2.76	-0.05	0.11	-0.33	0.80	0.19	0.23	0.09	0.55
PM_NO3_N	AQ_DE1_HTAP	393	0.37	-0.55	1.61	-0.14	0.23	-0.43	0.68	0.32	0.43	-0.10	0.45
PM_NO3_N	AQ_DK1_HTAP	382	0.64	0.40	0.59	0.16	0.22	0.50	0.67	0.31	0.68	-0.06	0.47
PM_NO3_N	AQ_FI1_MACC	393	0.63	0.05	0.70	0.02	0.18	0.05	0.56	0.28	0.53	0.10	0.55
PM_NO3_N	AQ_FI1_HTAP	393	0.64	0.32	0.76	0.13	0.23	0.39	0.70	0.34	0.54	-0.12	0.44
PM_NO3_N	AQ_FRES1_HTAP	393	0.44	-0.08	0.68	-0.03	0.19	-0.08	0.58	0.26	0.71	0.07	0.53
PM_NO3_N	AQ_UK1_MACC	382	0.68	0.29	0.68	0.11	0.20	0.35	0.63	0.31	0.66	0.01	0.50
PM_NO3_N	AQ_UK2_HTAP	370	0.66	0.35	0.90	0.14	0.24	0.42	0.71	0.38	0.65	-0.14	0.43
PM_NO3_N	ED_CHIM	382	0.46	0.62	1.38	0.29	0.37	0.90	1.12	0.53	0.68	-0.78	0.11
PM_NO3_N	ED_CMAQ	382	0.50	0.74	1.85	0.39	0.41	1.19	1.27	0.66	0.67	-1.02	-0.01
PM_NO3_N	ED_EMEP	382	0.62	0.37	0.66	0.15	0.23	0.45	0.69	0.32	0.58	-0.10	0.45
PM_NO3_N	ED_LOTO	382	0.67	0.34	0.64	0.13	0.21	0.41	0.63	0.31	0.68	0.00	0.50
PM_NO3_N	ED_MATCH	382	0.76	0.04	0.39	0.01	0.14	0.04	0.43	0.21	0.74	0.32	0.66
PM_NO3_N	ED_MINNI	382	0.45	0.32	0.91	0.12	0.26	0.37	0.79	0.36	0.63	-0.25	0.37
PM_NO3_N	ENSEMBLE	382	0.70	0.34	0.48	0.13	0.18	0.40	0.56	0.27	0.72	0.11	0.56
WNH4_N	AQ_DE1_HTAP	690	0.39	-0.77	2.14	-15.30	17.36	-0.55	0.63	26.96	0.62	0.07	0.53
WNH4_N	AQ_DK1_HTAP	704	0.59	-0.03	0.84	-0.91	15.54	-0.03	0.58	24.17	0.53	0.16	0.58
WNH4_N	AQ_FI1_MACC	715	0.59	-0.16	0.82	-3.87	14.39	-0.14	0.54	22.39	0.60	0.23	0.61
WNH4_N	AQ_FI1_HTAP	715	0.59	-0.12	0.82	-2.99	14.79	-0.11	0.55	22.86	0.59	0.20	0.60
WNH4_N	AQ_FRES1_HTAP	716	0.39	-0.80	2.53	-15.30	17.11	-0.57	0.64	27.86	0.54	0.08	0.54
WNH4_N	AQ_UK1_MACC	716	0.45	-0.61	1.82	-12.55	16.59	-0.47	0.62	26.33	0.53	0.11	0.55
WNH4_N	AQ_UK2_HTAP	714	0.53	-0.26	1.11	-6.25	16.12	-0.23	0.60	24.73	0.51	0.13	0.57
WNH4_N	ED_CHIM	704	0.49	-0.47	1.50	-10.17	16.07	-0.38	0.60	25.86	0.49	0.14	0.57
WNH4_N	ED_CMAQ	704	0.55	-0.55	1.55	-11.62	14.81	-0.43	0.55	25.12	0.59	0.20	0.60
WNH4_N	ED_EMEP	704	0.63	-0.01	0.67	-0.29	14.53	-0.01	0.54	21.85	0.64	0.22	0.61

Variable	Model	n	FAC2	FB	NMSE	MB	MGE	NMB	NMGE	RMSE	r	COE	IOA
WNH4_N	ED_LOTO	704	0.54	-0.35	1.21	-8.06	15.00	-0.30	0.56	24.65	0.53	0.19	0.60
WNH4_N	ED_MATCH	704	0.61	0.17	0.70	4.82	16.00	0.18	0.60	24.42	0.62	0.14	0.57
WNH4_N	ED_MINNI	704	0.35	-0.81	2.36	-15.47	16.92	-0.58	0.63	26.75	0.63	0.09	0.54
WNH4_N	ENSEMBLE	703	0.68	-0.12	0.69	-2.99	13.14	-0.11	0.49	20.97	0.65	0.29	0.65
NH3_N	AQ_DE1_HTAP	166	0.42	0.47	1.97	0.52	0.96	0.62	1.14	1.50	-0.08	-0.93	0.04
NH3_N	AQ_DK1_HTAP	154	0.48	0.46	1.17	0.50	0.80	0.59	0.96	1.14	0.39	-0.55	0.23
NH3_N	AQ_FI1_MACC	166	0.43	0.66	3.14	0.83	1.21	0.98	1.43	2.10	0.32	-1.41	-0.17
NH3_N	AQ_FI1_HTAP	166	0.49	0.69	2.92	0.88	1.16	1.04	1.38	2.06	0.34	-1.32	-0.14
NH3_N	AQ_FRES1_HTAP	166	0.45	0.62	1.92	0.76	1.06	0.90	1.26	1.61	0.40	-1.11	-0.05
NH3_N	AQ_UK1_MACC	154	0.48	0.37	2.07	0.38	0.80	0.46	0.96	1.45	0.40	-0.54	0.23
NH3_N	AQ_UK2_HTAP	154	0.32	0.89	2.83	1.33	1.55	1.59	1.86	2.26	0.49	-2.00	-0.33
NH3_N	ED_CHIM	154	0.44	0.16	0.69	0.14	0.60	0.17	0.72	0.75	0.52	-0.16	0.42
NH3_N	ED_CMAQ	154	0.48	0.28	1.48	0.27	0.75	0.32	0.89	1.17	0.26	-0.45	0.28
NH3_N	ED_EMEP	154	0.48	0.01	0.59	0.01	0.52	0.01	0.62	0.64	0.53	0.00	0.50
NH3_N	ED_LOTO	154	0.40	0.64	1.73	0.78	1.05	0.93	1.26	1.53	0.59	-1.04	-0.02
NH3_N	ED_MATCH	154	0.49	0.07	0.72	0.06	0.56	0.07	0.67	0.73	0.45	-0.08	0.46
NH3_N	ED_MINNI	154	0.40	0.29	0.75	0.28	0.68	0.34	0.82	0.84	0.48	-0.32	0.34
NH3_N	ENSEMBLE	154	0.46	0.45	1.22	0.48	0.81	0.58	0.97	1.16	0.47	-0.57	0.21
PM_NH4_N	AQ_DE1_HTAP	350	0.63	-0.10	0.72	-0.06	0.33	-0.10	0.55	0.49	0.56	0.19	0.59
PM_NH4_N	AQ_DK1_HTAP	339	0.73	0.14	0.45	0.09	0.30	0.15	0.49	0.44	0.69	0.30	0.65
PM_NH4_N	AQ_FI1_MACC	350	0.61	-0.15	0.70	-0.09	0.31	-0.14	0.51	0.47	0.61	0.25	0.63
PM_NH4_N	AQ_FI1_HTAP	350	0.64	-0.03	0.64	-0.02	0.32	-0.03	0.52	0.48	0.60	0.23	0.62
PM_NH4_N	AQ_FRES1_HTAP	350	0.72	0.29	0.47	0.20	0.32	0.33	0.53	0.48	0.75	0.23	0.61
PM_NH4_N	AQ_UK1_MACC	339	0.71	0.23	0.51	0.16	0.31	0.27	0.52	0.49	0.74	0.25	0.63
PM_NH4_N	AQ_UK2_HTAP	327	0.66	0.29	0.72	0.21	0.39	0.34	0.63	0.61	0.67	0.10	0.55
PM_NH4_N	ED_CHIM	339	0.66	0.47	0.67	0.38	0.44	0.62	0.72	0.63	0.75	-0.04	0.48
PM_NH4_N	ED_CMAQ	339	0.56	0.63	1.29	0.56	0.61	0.92	0.99	0.96	0.73	-0.43	0.28

Variable	Model	n	FAC2	FB	NMSE	MB	MGE	NMB	NMGE	RMSE	r	COE	IOA
PM_NH4_N	ED_EMEP	339	0.72	0.13	0.54	0.09	0.31	0.14	0.50	0.48	0.66	0.27	0.64
PM_NH4_N	ED_LOTO	339	0.74	0.14	0.46	0.09	0.30	0.15	0.49	0.44	0.70	0.30	0.65
PM_NH4_N	ED_MATCH	339	0.73	-0.10	0.48	-0.06	0.26	-0.10	0.43	0.40	0.73	0.37	0.69
PM_NH4_N	ED_MINNI	339	0.66	0.36	0.65	0.27	0.38	0.44	0.62	0.59	0.69	0.10	0.55
PM_NH4_N	ENSEMBLE	339	0.78	0.11	0.40	0.07	0.27	0.11	0.44	0.41	0.73	0.37	0.69
WSO4_S	AQ_DE1_HTAP	692	0.39	-0.67	1.92	-13.89	17.44	-0.50	0.63	26.88	0.55	0.06	0.53
WSO4_S	AQ_DK1_HTAP	704	0.30	-0.99	3.89	-17.75	19.47	-0.66	0.73	30.67	0.42	-0.05	0.48
WSO4_S	AQ_FI1_MACC	715	0.47	-0.51	1.65	-10.82	16.64	-0.40	0.62	26.60	0.46	0.10	0.55
WSO4_S	AQ_FI1_HTAP	715	0.46	-0.54	1.73	-11.39	16.62	-0.42	0.62	26.75	0.46	0.10	0.55
WSO4_S	AQ_FRES1_HTAP	716	0.30	-1.04	4.27	-18.31	19.67	-0.68	0.73	31.09	0.44	-0.06	0.47
WSO4_S	AQ_UK1_MACC	716	0.45	-0.47	1.53	-10.14	16.90	-0.38	0.63	26.14	0.49	0.09	0.54
WSO4_S	AQ_UK2_HTAP	714	0.51	-0.08	1.03	-2.05	17.58	-0.08	0.65	26.18	0.43	0.05	0.53
WSO4_S	ED_CHIM	704	0.24	-1.20	5.81	-20.09	20.72	-0.75	0.77	32.26	0.48	-0.11	0.44
WSO4_S	ED_CMAQ	704	0.55	-0.34	1.36	-7.81	15.89	-0.29	0.59	26.33	0.40	0.15	0.57
WSO4_S	ED_EMEP	704	0.52	-0.11	1.04	-2.75	17.34	-0.10	0.65	25.93	0.41	0.07	0.53
WSO4_S	ED_LOTO	704	0.39	-0.63	2.32	-12.77	18.72	-0.48	0.70	29.54	0.29	-0.01	0.50
WSO4_S	ED_MATCH	704	0.51	0.12	1.15	3.29	20.15	0.12	0.75	30.50	0.35	-0.08	0.46
WSO4_S	ED_MINNI	704	0.28	-0.98	3.53	-17.67	18.88	-0.66	0.71	29.34	0.55	-0.02	0.49
WSO4_S	ENSEMBLE	703	0.53	-0.29	1.21	-6.83	16.11	-0.25	0.60	25.47	0.45	0.13	0.57
SO2_S	AQ_DE1_HTAP	695	0.39	0.49	2.45	0.36	0.66	0.65	1.19	1.12	0.31	-0.34	0.33
SO2_S	AQ_DK1_HTAP	671	0.59	0.14	2.07	0.09	0.41	0.15	0.72	0.88	0.54	0.19	0.60
SO2_S	AQ_FI1_MACC	695	0.50	0.48	2.43	0.36	0.57	0.64	1.03	1.11	0.52	-0.16	0.42
SO2_S	AQ_FI1_HTAP	695	0.57	0.36	2.52	0.24	0.48	0.43	0.86	1.06	0.48	0.03	0.52
SO2_S	AQ_FRES1_HTAP	695	0.53	-0.05	2.69	-0.03	0.42	-0.05	0.75	0.89	0.42	0.16	0.58
SO2_S	AQ_UK1_MACC	683	0.50	0.51	1.57	0.39	0.54	0.69	0.96	0.92	0.62	-0.08	0.46
SO2_S	AQ_UK2_HTAP	671	0.36	0.70	2.32	0.62	0.77	1.08	1.35	1.25	0.55	-0.52	0.24
SO2_S	ED_CHIM	671	0.56	0.28	2.70	0.18	0.48	0.32	0.84	1.08	0.48	0.05	0.53

Variable	Model	n	FAC2	FB	NMSE	MB	MGE	NMB	NMGE	RMSE	r	COE	IOA
SO2_S	ED_CMAQ	671	0.35	0.73	2.56	0.65	0.80	1.14	1.40	1.34	0.56	-0.58	0.21
SO2_S	ED_EMEP	671	0.51	-0.21	2.63	-0.11	0.38	-0.19	0.66	0.83	0.51	0.25	0.63
SO2_S	ED_LOTO	671	0.59	0.21	2.11	0.13	0.43	0.23	0.76	0.92	0.50	0.14	0.57
SO2_S	ED_MATCH	671	0.52	0.22	1.93	0.14	0.44	0.25	0.78	0.89	0.51	0.12	0.56
SO2_S	ED_MINNI	671	0.41	0.44	1.56	0.32	0.55	0.57	0.96	0.89	0.55	-0.08	0.46
SO2_S	ENSEMBLE	671	0.58	0.23	1.89	0.15	0.42	0.26	0.73	0.88	0.54	0.18	0.59
PM_SO4_S	AQ_DE1_HTAP	288	0.75	-0.01	0.38	-0.01	0.19	-0.01	0.44	0.26	0.50	0.13	0.57
PM_SO4_S	AQ_DK1_HTAP	265	0.74	0.44	0.70	0.24	0.28	0.56	0.67	0.44	0.57	-0.39	0.30
PM_SO4_S	AQ_FI1_MACC	288	0.57	-0.26	0.89	-0.10	0.22	-0.23	0.51	0.35	0.37	-0.01	0.49
PM_SO4_S	AQ_FI1_HTAP	288	0.55	-0.30	0.83	-0.11	0.21	-0.26	0.50	0.33	0.40	0.01	0.50
PM_SO4_S	AQ_FRES1_HTAP	288	0.86	0.15	0.29	0.07	0.15	0.16	0.36	0.25	0.69	0.29	0.65
PM_SO4_S	AQ_UK1_MACC	277	0.79	0.06	0.28	0.02	0.15	0.06	0.37	0.22	0.65	0.27	0.64
PM_SO4_S	AQ_UK2_HTAP	277	0.72	0.01	0.48	0.01	0.18	0.01	0.45	0.28	0.45	0.11	0.56
PM_SO4_S	ED_CHIM	265	0.83	-0.22	0.32	-0.08	0.15	-0.20	0.36	0.21	0.68	0.26	0.63
PM_SO4_S	ED_CMAQ	265	0.74	0.33	0.28	0.17	0.20	0.40	0.48	0.27	0.71	0.00	0.50
PM_SO4_S	ED_EMEP	265	0.85	-0.02	0.27	-0.01	0.14	-0.02	0.33	0.22	0.74	0.31	0.66
PM_SO4_S	ED_LOTO	265	0.71	-0.32	0.53	-0.12	0.19	-0.28	0.45	0.26	0.54	0.08	0.54
PM_SO4_S	ED_MATCH	265	0.75	-0.31	0.43	-0.11	0.16	-0.27	0.39	0.24	0.64	0.20	0.60
PM_SO4_S	ED_MINNI	265	0.83	-0.14	0.25	-0.06	0.14	-0.13	0.32	0.20	0.74	0.34	0.67
PM_SO4_S	ENSEMBLE	265	0.79	-0.23	0.41	-0.09	0.16	-0.21	0.37	0.24	0.61	0.24	0.62
PRECIP	AQ_DE1_HTAP	661	0.63	-0.33	0.60	-23.45	36.92	-0.28	0.44	54.65	0.68	0.23	0.62
PRECIP	AQ_DK1_HTAP	668	0.71	0.05	0.54	3.98	40.01	0.05	0.49	60.94	0.61	0.17	0.58
PRECIP	AQ_FI1_MACC	706	0.81	0.11	0.37	9.43	30.81	0.12	0.39	50.88	0.71	0.36	0.68
PRECIP	AQ_FI1_HTAP	706	0.81	0.11	0.37	9.43	30.81	0.12	0.39	50.88	0.71	0.36	0.68
PRECIP	AQ_FRES1_HTAP	708	0.83	0.11	0.26	9.48	28.27	0.12	0.36	42.91	0.79	0.41	0.70
PRECIP	AQ_UK1_MACC	695	0.63	-0.20	0.56	-14.57	37.43	-0.18	0.47	54.53	0.66	0.22	0.61
PRECIP	AQ_UK2_HTAP	681	0.68	-0.17	0.47	-12.82	35.49	-0.16	0.44	51.37	0.68	0.26	0.63

Variable	Model	n	FAC2	FB	NMSE	MB	MGE	NMB	NMGE	RMSE	r	COE	IOA
PRECIP	ED_CHIM	708	0.66	-0.11	0.52	-8.39	36.46	-0.11	0.46	53.92	0.62	0.24	0.62
PRECIP	ED_CMAQ	708	0.75	-0.06	0.39	-4.47	32.03	-0.06	0.40	48.39	0.70	0.33	0.67
PRECIP	ED_EMEP	708	0.66	-0.11	0.52	-8.39	36.46	-0.11	0.46	53.92	0.62	0.24	0.62
PRECIP	ED_LOTO	708	0.68	-0.11	0.59	-8.50	38.21	-0.11	0.48	57.73	0.57	0.20	0.60
PRECIP	ED_MATCH	708	0.76	-0.01	0.35	-0.40	30.30	-0.01	0.38	47.15	0.73	0.37	0.68
PRECIP	ED_MINNI	708	0.66	-0.11	0.52	-8.39	36.46	-0.11	0.46	53.92	0.62	0.24	0.62
TNO3_N	AQ_DE1_HTAP	554	0.41	-0.70	1.46	-0.25	0.29	-0.52	0.59	0.41	0.52	-0.04	0.48
TNO3_N	AQ_DK1_HTAP	544	0.77	0.27	0.31	0.15	0.23	0.31	0.47	0.31	0.73	0.18	0.59
TNO3_N	AQ_FI1_MACC	554	0.80	-0.06	0.45	-0.03	0.21	-0.05	0.44	0.32	0.59	0.23	0.61
TNO3_N	AQ_FI1_HTAP	554	0.74	0.22	0.49	0.12	0.26	0.25	0.53	0.38	0.57	0.07	0.54
TNO3_N	AQ_FRES1_HTAP	554	0.73	-0.13	0.31	-0.06	0.18	-0.12	0.37	0.26	0.77	0.35	0.68
TNO3_N	AQ_UK1_MACC	544	0.84	0.20	0.31	0.11	0.20	0.23	0.41	0.30	0.75	0.28	0.64
TNO3_N	AQ_UK2_HTAP	544	0.85	0.17	0.40	0.09	0.21	0.18	0.44	0.34	0.74	0.24	0.62
TNO3_N	ED_CHIM	544	0.63	0.53	0.73	0.36	0.39	0.73	0.79	0.55	0.76	-0.39	0.31
TNO3_N	ED_CMAQ	544	0.53	0.65	1.05	0.47	0.49	0.97	1.00	0.70	0.74	-0.75	0.12
TNO3_N	ED_EMEP	544	0.72	0.23	0.43	0.13	0.26	0.26	0.52	0.36	0.60	0.08	0.54
TNO3_N	ED_LOTO	544	0.81	0.17	0.30	0.09	0.20	0.18	0.41	0.29	0.73	0.29	0.64
TNO3_N	ED_MATCH	544	0.83	0.05	0.26	0.03	0.17	0.06	0.35	0.25	0.75	0.38	0.69
TNO3_N	ED_MINNI	544	0.79	0.20	0.37	0.11	0.22	0.22	0.44	0.33	0.74	0.22	0.61
TNO3_N	ENSEMBLE	544	0.81	0.22	0.27	0.12	0.20	0.25	0.41	0.28	0.76	0.28	0.64
TNH4_N	AQ_DE1_HTAP	472	0.59	0.10	1.01	0.15	0.96	0.11	0.66	1.53	0.29	-0.11	0.44
TNH4_N	AQ_DK1_HTAP	461	0.74	0.23	0.46	0.38	0.74	0.26	0.51	1.10	0.69	0.15	0.57
TNH4_N	AQ_FI1_MACC	472	0.72	0.19	0.79	0.30	0.84	0.21	0.58	1.41	0.54	0.03	0.51
TNH4_N	AQ_FI1_HTAP	472	0.72	0.22	0.77	0.36	0.83	0.25	0.57	1.41	0.55	0.04	0.52
TNH4_N	AQ_FRES1_HTAP	472	0.74	0.32	0.64	0.55	0.87	0.38	0.60	1.36	0.65	0.00	0.50
TNH4_N	AQ_UK1_MACC	461	0.72	0.17	0.69	0.26	0.78	0.18	0.54	1.30	0.57	0.11	0.55
TNH4_N	AQ_UK2_HTAP	461	0.54	0.66	1.40	1.42	1.58	0.98	1.09	2.41	0.76	-0.81	0.10

Variable	Model	n	FAC2	FB	NMSE	MB	MGE	NMB	NMGE	RMSE	r	COE	IOA
TNH4_N	ED_CHIM	461	0.73	0.24	0.42	0.39	0.73	0.27	0.51	1.06	0.71	0.16	0.58
TNH4_N	ED_CMAQ	461	0.64	0.36	0.74	0.63	0.99	0.43	0.68	1.49	0.58	-0.13	0.43
TNH4_N	ED_EMEP	461	0.79	0.01	0.35	0.02	0.57	0.01	0.39	0.86	0.73	0.35	0.68
TNH4_N	ED_LOTO	461	0.74	0.34	0.63	0.60	0.86	0.41	0.60	1.36	0.68	0.01	0.51
TNH4_N	ED_MATCH	461	0.79	-0.10	0.39	-0.14	0.56	-0.10	0.39	0.86	0.73	0.36	0.68
TNH4_N	ED_MINNI	461	0.73	0.24	0.42	0.39	0.74	0.27	0.51	1.06	0.69	0.15	0.57
TNH4_N	ENSEMBLE	461	0.79	0.17	0.40	0.28	0.67	0.19	0.46	1.00	0.70	0.23	0.62
TSO4_S	AQ_DE1_HTAP	245	0.57	0.37	0.80	0.34	0.57	0.45	0.76	0.81	0.52	-0.39	0.31
TSO4_S	AQ_DK1_HTAP	222	0.80	0.35	0.48	0.32	0.38	0.42	0.49	0.64	0.81	0.08	0.54
TSO4_S	AQ_FI1_MACC	245	0.74	0.18	0.57	0.15	0.39	0.20	0.51	0.62	0.71	0.06	0.53
TSO4_S	AQ_FI1_HTAP	245	0.79	0.06	0.37	0.04	0.28	0.06	0.37	0.47	0.75	0.33	0.66
TSO4_S	AQ_FRES1_HTAP	245	0.81	0.07	0.44	0.05	0.32	0.07	0.43	0.52	0.59	0.22	0.61
TSO4_S	AQ_UK1_MACC	234	0.84	0.26	0.44	0.22	0.35	0.30	0.48	0.56	0.68	0.15	0.57
TSO4_S	AQ_UK2_HTAP	234	0.72	0.37	0.76	0.33	0.50	0.45	0.67	0.77	0.63	-0.20	0.40
TSO4_S	ED_CHIM	222	0.84	0.00	0.52	0.00	0.29	0.00	0.38	0.55	0.76	0.28	0.64
TSO4_S	ED_CMAQ	222	0.65	0.52	0.90	0.54	0.62	0.70	0.80	0.95	0.77	-0.50	0.25
TSO4_S	ED_EMEP	222	0.81	-0.13	0.42	-0.09	0.29	-0.12	0.38	0.46	0.73	0.29	0.64
TSO4_S	ED_LOTO	222	0.83	-0.08	0.40	-0.06	0.29	-0.07	0.37	0.47	0.75	0.30	0.65
TSO4_S	ED_MATCH	222	0.81	-0.08	0.38	-0.06	0.30	-0.08	0.40	0.45	0.72	0.26	0.63
TSO4_S	ED_MINNI	222	0.80	0.20	0.46	0.17	0.36	0.22	0.47	0.57	0.74	0.12	0.56
TSO4_S	ENSEMBLE	222	0.88	-0.02	0.28	-0.02	0.24	-0.02	0.32	0.40	0.81	0.41	0.71

S3.7 . Tables with complete statistics from annual values (definition in S3.10). All available sites

Variable	Model	n	FAC2	FB	NMSE	MB	MGE	NMB	NMGE	RMSE	r	COE	IOA
WNO3_N	AQ_DE1_HTAP	59	0.00	-1.50	7.77	-227.79	227.79	-0.86	0.86	279.67	0.70	-0.80	0.10
WNO3_N	AQ_DK1_HTAP	58	0.88	-0.09	0.29	-22.81	94.48	-0.08	0.35	138.04	0.64	0.25	0.63
WNO3_N	AQ_FI1_MACC	59	0.68	-0.45	0.58	-98.20	110.99	-0.37	0.42	160.47	0.72	0.12	0.56
WNO3_N	AQ_FI1_HTAP	59	0.92	-0.12	0.25	-30.28	77.75	-0.11	0.29	124.09	0.74	0.39	0.69
WNO3_N	AQ_FRES1_HTAP	59	0.86	-0.44	0.60	-95.39	104.40	-0.36	0.39	164.29	0.73	0.17	0.59
WNO3_N	AQ_UK1_MACC	59	0.61	-0.53	0.74	-111.10	121.67	-0.42	0.46	174.25	0.66	0.04	0.52
WNO3_N	AQ_UK2_HTAP	58	0.81	-0.30	0.38	-70.33	88.30	-0.26	0.33	141.62	0.73	0.30	0.65
WNO3_N	ED_CHIM	58	0.84	-0.34	0.50	-78.79	97.40	-0.29	0.36	159.14	0.66	0.23	0.61
WNO3_N	ED_CMAQ	58	0.91	-0.23	0.38	-56.18	91.54	-0.21	0.34	147.47	0.65	0.27	0.64
WNO3_N	ED_EMEP	58	0.93	-0.03	0.23	-9.12	83.17	-0.03	0.31	126.36	0.70	0.34	0.67
WNO3_N	ED_LOTO	58	0.69	-0.47	0.57	-101.85	113.84	-0.38	0.42	159.40	0.74	0.10	0.55
WNO3_N	ED_MATCH	58	0.91	0.07	0.18	19.16	86.91	0.07	0.32	118.49	0.76	0.31	0.66
WNO3_N	ED_MINNI	58	0.05	-1.16	3.33	-197.09	198.24	-0.73	0.74	252.68	0.63	-0.57	0.21
WNO3_N	ENSEMBLE	58	0.95	-0.19	0.26	-46.05	74.44	-0.17	0.28	125.09	0.77	0.41	0.71
HNO3_N	AQ_DE1_HTAP	13	0.15	-1.11	5.04	-0.10	0.11	-0.71	0.79	0.17	0.17	0.02	0.51
HNO3_N	AQ_DK1_HTAP	12	0.58	-0.35	1.18	-0.04	0.09	-0.30	0.60	0.13	0.47	0.26	0.63
HNO3_N	AQ_FI1_MACC	13	0.46	-0.82	2.81	-0.08	0.10	-0.58	0.72	0.15	0.41	0.10	0.55
HNO3_N	AQ_FI1_HTAP	13	0.62	-0.42	1.43	-0.05	0.09	-0.35	0.63	0.13	0.40	0.22	0.61
HNO3_N	AQ_FRES1_HTAP	13	0.54	-0.48	1.69	-0.05	0.09	-0.39	0.64	0.14	0.28	0.21	0.60
HNO3_N	AQ_UK1_MACC	12	0.67	-0.21	1.20	-0.03	0.09	-0.19	0.66	0.14	0.27	0.19	0.60
HNO3_N	AQ_UK2_HTAP	12	0.42	-0.67	2.33	-0.07	0.10	-0.50	0.73	0.15	0.19	0.11	0.55
HNO3_N	ED_CHIM	12	0.50	-0.11	1.26	-0.01	0.11	-0.10	0.74	0.15	0.15	0.09	0.55
HNO3_N	ED_CMAQ	12	0.58	0.08	1.29	0.01	0.11	0.09	0.77	0.17	0.11	0.05	0.52
HNO3_N	ED_EMEP	12	0.33	-0.67	2.18	-0.07	0.11	-0.50	0.77	0.15	0.35	0.06	0.53
HNO3_N	ED_LOTO	12	0.50	-0.44	1.54	-0.05	0.10	-0.36	0.71	0.14	0.30	0.12	0.56

Variable	Model	n	FAC2	FB	NMSE	MB	MGE	NMB	NMGE	RMSE	r	COE	IOA
HNO3_N	ED_MATCH	12	0.58	-0.17	1.22	-0.02	0.11	-0.15	0.79	0.15	0.12	0.04	0.52
HNO3_N	ED_MINNI	12	0.42	-0.46	1.83	-0.05	0.11	-0.37	0.75	0.15	0.07	0.08	0.54
HNO3_N	ENSEMBLE	12	0.58	-0.41	1.47	-0.05	0.10	-0.34	0.66	0.14	0.30	0.18	0.59
PM_NO3_N	AQ_DE1_HTAP	33	0.45	-0.57	0.97	-0.15	0.18	-0.44	0.55	0.25	0.52	-0.10	0.45
PM_NO3_N	AQ_DK1_HTAP	32	0.69	0.40	0.32	0.16	0.18	0.50	0.56	0.23	0.77	-0.08	0.46
PM_NO3_N	AQ_FI1_MACC	33	0.79	0.08	0.30	0.03	0.14	0.08	0.44	0.19	0.59	0.14	0.57
PM_NO3_N	AQ_FI1_HTAP	33	0.70	0.35	0.34	0.14	0.19	0.43	0.56	0.23	0.66	-0.11	0.45
PM_NO3_N	AQ_FRES1_HTAP	33	0.58	-0.11	0.29	-0.03	0.14	-0.10	0.42	0.17	0.74	0.16	0.58
PM_NO3_N	AQ_UK1_MACC	32	0.75	0.30	0.32	0.11	0.16	0.35	0.49	0.22	0.69	0.05	0.53
PM_NO3_N	AQ_UK2_HTAP	31	0.74	0.35	0.41	0.14	0.19	0.42	0.55	0.26	0.69	-0.11	0.44
PM_NO3_N	ED_CHIM	32	0.59	0.61	0.79	0.29	0.32	0.88	0.96	0.40	0.73	-0.86	0.07
PM_NO3_N	ED_CMAQ	32	0.50	0.75	1.04	0.39	0.40	1.19	1.22	0.50	0.71	-1.36	-0.15
PM_NO3_N	ED_EMEP	32	0.72	0.37	0.31	0.15	0.17	0.46	0.52	0.22	0.76	-0.01	0.49
PM_NO3_N	ED_LOTO	32	0.78	0.34	0.35	0.13	0.17	0.41	0.51	0.23	0.75	0.02	0.51
PM_NO3_N	ED_MATCH	32	0.81	0.04	0.16	0.01	0.10	0.04	0.32	0.14	0.81	0.39	0.69
PM_NO3_N	ED_MINNI	32	0.66	0.30	0.39	0.11	0.18	0.35	0.56	0.24	0.68	-0.08	0.46
PM_NO3_N	ENSEMBLE	32	0.75	0.34	0.24	0.13	0.15	0.41	0.46	0.19	0.82	0.10	0.55
WNH4_N	AQ_DE1_HTAP	61	0.34	-0.77	1.18	-175.31	178.63	-0.56	0.57	227.76	0.77	-0.29	0.36
WNH4_N	AQ_DK1_HTAP	60	0.85	-0.03	0.29	-8.19	120.15	-0.03	0.38	168.00	0.62	0.15	0.57
WNH4_N	AQ_FI1_MACC	61	0.87	-0.15	0.23	-43.58	100.68	-0.14	0.32	140.83	0.75	0.27	0.64
WNH4_N	AQ_FI1_HTAP	61	0.89	-0.11	0.23	-33.29	101.76	-0.11	0.32	142.16	0.74	0.27	0.63
WNH4_N	AQ_FRES1_HTAP	61	0.34	-0.80	1.34	-179.62	183.18	-0.57	0.58	239.02	0.60	-0.32	0.34
WNH4_N	AQ_UK1_MACC	61	0.54	-0.61	0.83	-146.99	161.42	-0.47	0.51	209.19	0.65	-0.16	0.42
WNH4_N	AQ_UK2_HTAP	61	0.79	-0.26	0.33	-71.80	114.49	-0.23	0.36	159.83	0.70	0.17	0.59
WNH4_N	ED_CHIM	60	0.57	-0.47	0.71	-119.51	149.64	-0.38	0.47	209.71	0.53	-0.06	0.47
WNH4_N	ED_CMAQ	60	0.65	-0.55	0.72	-135.88	142.47	-0.43	0.45	201.13	0.70	-0.01	0.49
WNH4_N	ED_EMEP	60	0.90	-0.01	0.16	-2.02	90.99	-0.01	0.29	126.65	0.81	0.35	0.68

Variable	Model	n	FAC2	FB	NMSE	MB	MGE	NMB	NMGE	RMSE	r	COE	IOA
WNH4_N	ED_LOTO	60	0.82	-0.35	0.38	-93.79	125.30	-0.30	0.40	163.54	0.74	0.11	0.56
WNH4_N	ED_MATCH	60	0.82	0.17	0.31	59.53	123.99	0.19	0.39	190.34	0.72	0.12	0.56
WNH4_N	ED_MINNI	60	0.30	-0.81	1.30	-181.83	184.02	-0.58	0.58	234.07	0.78	-0.31	0.35
WNH4_N	ENSEMBLE	60	0.93	-0.11	0.21	-33.40	92.82	-0.11	0.29	134.95	0.76	0.34	0.67
NH3_N	AQ_DE1_HTAP	13	0.54	0.40	0.59	0.42	0.62	0.50	0.74	0.78	0.03	-0.83	0.08
NH3_N	AQ_DK1_HTAP	12	0.67	0.43	0.79	0.46	0.65	0.55	0.79	0.91	0.67	-0.83	0.08
NH3_N	AQ_FI1_MACC	13	0.46	0.66	2.59	0.81	1.09	0.98	1.31	1.89	0.59	-2.25	-0.38
NH3_N	AQ_FI1_HTAP	13	0.62	0.68	2.37	0.87	1.02	1.04	1.23	1.83	0.62	-2.04	-0.34
NH3_N	AQ_FRES1_HTAP	13	0.46	0.62	1.59	0.75	0.96	0.89	1.15	1.45	0.64	-1.84	-0.30
NH3_N	AQ_UK1_MACC	12	0.67	0.37	1.21	0.37	0.63	0.45	0.76	1.09	0.63	-0.77	0.11
NH3_N	AQ_UK2_HTAP	12	0.42	0.85	2.31	1.21	1.39	1.47	1.68	1.97	0.70	-2.90	-0.49
NH3_N	ED_CHIM	12	0.58	0.11	0.37	0.10	0.46	0.12	0.56	0.53	0.71	-0.30	0.35
NH3_N	ED_CMAQ	12	0.58	0.28	1.17	0.27	0.60	0.32	0.73	1.03	0.34	-0.69	0.16
NH3_N	ED_EMEP	12	0.67	-0.04	0.27	-0.03	0.34	-0.04	0.42	0.42	0.70	0.03	0.52
NH3_N	ED_LOTO	12	0.58	0.63	1.61	0.76	0.92	0.92	1.12	1.45	0.77	-1.60	-0.23
NH3_N	ED_MATCH	12	0.58	0.06	0.44	0.05	0.43	0.06	0.52	0.56	0.57	-0.21	0.39
NH3_N	ED_MINNI	12	0.50	0.25	0.45	0.24	0.54	0.29	0.65	0.63	0.64	-0.51	0.25
NH3_N	ENSEMBLE	12	0.67	0.44	0.97	0.46	0.69	0.56	0.84	1.01	0.69	-0.95	0.02
PM_NH4_N	AQ_DE1_HTAP	28	0.79	-0.07	0.31	-0.04	0.23	-0.07	0.40	0.31	0.70	0.39	0.69
PM_NH4_N	AQ_DK1_HTAP	27	0.74	0.17	0.25	0.11	0.23	0.19	0.39	0.32	0.74	0.40	0.70
PM_NH4_N	AQ_FI1_MACC	28	0.68	-0.12	0.38	-0.07	0.24	-0.11	0.42	0.34	0.64	0.36	0.68
PM_NH4_N	AQ_FI1_HTAP	28	0.82	0.00	0.29	0.00	0.23	0.00	0.40	0.32	0.69	0.39	0.69
PM_NH4_N	AQ_FRES1_HTAP	28	0.75	0.32	0.28	0.22	0.26	0.37	0.45	0.37	0.78	0.31	0.66
PM_NH4_N	AQ_UK1_MACC	27	0.78	0.25	0.29	0.17	0.25	0.29	0.42	0.36	0.76	0.36	0.68
PM_NH4_N	AQ_UK2_HTAP	26	0.77	0.31	0.46	0.22	0.34	0.37	0.56	0.48	0.71	0.15	0.58
PM_NH4_N	ED_CHIM	27	0.74	0.48	0.45	0.38	0.38	0.63	0.64	0.51	0.81	0.03	0.51
PM_NH4_N	ED_CMAQ	27	0.56	0.65	0.77	0.57	0.57	0.96	0.96	0.73	0.78	-0.46	0.27

Variable	Model	n	FAC2	FB	NMSE	MB	MGE	NMB	NMGE	RMSE	r	COE	IOA
PM_NH4_N	ED_EMEP	27	0.78	0.17	0.26	0.11	0.23	0.18	0.39	0.33	0.75	0.41	0.71
PM_NH4_N	ED_LOTO	27	0.78	0.17	0.24	0.11	0.21	0.18	0.36	0.32	0.78	0.45	0.73
PM_NH4_N	ED_MATCH	27	0.81	-0.07	0.24	-0.04	0.21	-0.07	0.36	0.28	0.78	0.45	0.73
PM_NH4_N	ED_MINNI	27	0.74	0.39	0.35	0.28	0.31	0.48	0.53	0.43	0.77	0.20	0.60
PM_NH4_N	ENSEMBLE	27	0.78	0.14	0.21	0.09	0.20	0.15	0.34	0.29	0.78	0.49	0.75
WSO4_S	AQ_DE1_HTAP	61	0.72	-0.50	1.02	-101.28	116.67	-0.40	0.46	198.53	0.55	0.09	0.54
WSO4_S	AQ_DK1_HTAP	59	0.34	-0.82	1.87	-149.52	152.26	-0.58	0.59	227.10	0.65	-0.17	0.42
WSO4_S	AQ_FI1_MACC	61	0.80	-0.30	0.47	-67.02	78.54	-0.26	0.31	150.17	0.75	0.39	0.69
WSO4_S	AQ_FI1_HTAP	61	0.82	-0.34	0.52	-73.15	79.73	-0.29	0.31	153.87	0.76	0.38	0.69
WSO4_S	AQ_FRES1_HTAP	61	0.33	-0.87	1.99	-153.64	153.64	-0.60	0.60	225.45	0.76	-0.20	0.40
WSO4_S	AQ_UK1_MACC	61	0.80	-0.27	0.70	-59.95	99.36	-0.24	0.39	186.09	0.49	0.22	0.61
WSO4_S	AQ_UK2_HTAP	60	0.85	0.14	0.35	38.89	110.23	0.15	0.43	162.01	0.64	0.15	0.57
WSO4_S	ED_CHIM	59	0.20	-1.07	3.35	-178.72	178.72	-0.70	0.70	259.29	0.60	-0.37	0.31
WSO4_S	ED_CMAQ	59	0.85	-0.14	0.59	-34.54	95.94	-0.13	0.37	183.13	0.49	0.26	0.63
WSO4_S	ED_EMEP	59	0.90	0.10	0.36	27.86	103.39	0.11	0.40	162.36	0.64	0.21	0.60
WSO4_S	ED_LOTO	59	0.75	-0.42	0.72	-89.42	101.21	-0.35	0.39	175.85	0.68	0.22	0.61
WSO4_S	ED_MATCH	59	0.80	0.33	0.40	101.40	132.97	0.39	0.52	191.78	0.77	-0.02	0.49
WSO4_S	ED_MINNI	59	0.32	-0.83	1.89	-150.73	151.05	-0.59	0.59	227.12	0.67	-0.16	0.42
WSO4_S	ENSEMBLE	59	0.93	-0.08	0.29	-19.75	69.14	-0.08	0.27	132.35	0.77	0.47	0.73
SO2_S	AQ_DE1_HTAP	58	0.48	0.50	1.83	0.38	0.60	0.67	1.07	0.98	0.31	-0.38	0.31
SO2_S	AQ_DK1_HTAP	56	0.79	0.14	1.26	0.09	0.33	0.15	0.57	0.69	0.53	0.26	0.63
SO2_S	AQ_FI1_MACC	58	0.53	0.49	1.31	0.36	0.54	0.64	0.97	0.82	0.49	-0.25	0.37
SO2_S	AQ_FI1_HTAP	58	0.60	0.36	1.40	0.25	0.44	0.44	0.79	0.79	0.45	-0.02	0.49
SO2_S	AQ_FRES1_HTAP	58	0.62	-0.04	1.86	-0.02	0.34	-0.04	0.61	0.74	0.43	0.21	0.61
SO2_S	AQ_UK1_MACC	57	0.63	0.52	1.05	0.39	0.51	0.70	0.90	0.75	0.67	-0.16	0.42
SO2_S	AQ_UK2_HTAP	56	0.38	0.70	1.62	0.62	0.75	1.08	1.32	1.05	0.57	-0.71	0.15
SO2_S	ED_CHIM	56	0.59	0.28	1.49	0.18	0.43	0.32	0.76	0.80	0.47	0.01	0.51

Variable	Model	n	FAC2	FB	NMSE	MB	MGE	NMB	NMGE	RMSE	r	COE	IOA
SO2_S	ED_CMAQ	56	0.34	0.73	1.62	0.65	0.78	1.14	1.36	1.06	0.56	-0.76	0.12
SO2_S	ED_EMEP	56	0.55	-0.21	1.78	-0.11	0.32	-0.19	0.55	0.69	0.54	0.28	0.64
SO2_S	ED_LOTO	56	0.66	0.20	1.33	0.13	0.38	0.23	0.66	0.73	0.50	0.14	0.57
SO2_S	ED_MATCH	56	0.57	0.22	1.28	0.14	0.40	0.25	0.70	0.72	0.52	0.09	0.54
SO2_S	ED_MINNI	56	0.41	0.44	1.14	0.32	0.51	0.57	0.89	0.76	0.55	-0.15	0.42
SO2_S	ENSEMBLE	56	0.64	0.23	1.23	0.15	0.38	0.26	0.67	0.71	0.53	0.13	0.56
PM_SO4_S	AQ_DE1_HTAP	23	0.87	0.00	0.22	0.00	0.12	0.00	0.29	0.20	0.55	0.34	0.67
PM_SO4_S	AQ_DK1_HTAP	21	0.81	0.46	0.65	0.26	0.26	0.60	0.62	0.44	0.55	-0.53	0.24
PM_SO4_S	AQ_FI1_MACC	23	0.61	-0.25	0.57	-0.09	0.19	-0.22	0.45	0.28	0.40	-0.03	0.49
PM_SO4_S	AQ_FI1_HTAP	23	0.65	-0.29	0.51	-0.11	0.20	-0.25	0.47	0.26	0.46	-0.06	0.47
PM_SO4_S	AQ_FRES1_HTAP	23	0.91	0.18	0.24	0.08	0.12	0.20	0.29	0.23	0.64	0.35	0.67
PM_SO4_S	AQ_UK1_MACC	22	0.77	0.06	0.19	0.03	0.11	0.06	0.26	0.18	0.63	0.41	0.70
PM_SO4_S	AQ_UK2_HTAP	22	0.82	0.03	0.26	0.01	0.13	0.03	0.31	0.21	0.57	0.27	0.64
PM_SO4_S	ED_CHIM	21	0.86	-0.20	0.24	-0.08	0.15	-0.18	0.34	0.19	0.59	0.16	0.58
PM_SO4_S	ED_CMAQ	21	0.71	0.34	0.24	0.18	0.19	0.41	0.44	0.25	0.63	-0.08	0.46
PM_SO4_S	ED_EMEP	21	0.95	0.00	0.19	0.00	0.10	0.00	0.24	0.19	0.69	0.42	0.71
PM_SO4_S	ED_LOTO	21	0.71	-0.32	0.32	-0.12	0.17	-0.27	0.40	0.21	0.61	0.02	0.51
PM_SO4_S	ED_MATCH	21	0.71	-0.28	0.32	-0.11	0.17	-0.25	0.39	0.21	0.57	0.04	0.52
PM_SO4_S	ED_MINNI	21	0.86	-0.13	0.18	-0.05	0.11	-0.12	0.27	0.17	0.71	0.34	0.67
PM_SO4_S	ENSEMBLE	21	0.86	-0.21	0.29	-0.08	0.15	-0.19	0.36	0.21	0.59	0.12	0.56
PRECIP	AQ_DE1_HTAP	58	0.90	-0.34	0.28	-281.73	315.20	-0.29	0.33	426.21	0.66	0.06	0.53
PRECIP	AQ_DK1_HTAP	56	0.95	0.05	0.15	47.22	282.85	0.05	0.29	389.18	0.65	0.15	0.58
PRECIP	AQ_FI1_MACC	59	0.90	0.11	0.14	112.66	240.34	0.12	0.25	375.83	0.65	0.29	0.64
PRECIP	AQ_FI1_HTAP	59	0.90	0.11	0.14	112.66	240.34	0.12	0.25	375.83	0.65	0.29	0.64
PRECIP	AQ_FRES1_HTAP	59	0.93	0.11	0.09	113.76	209.91	0.12	0.22	294.53	0.78	0.38	0.69
PRECIP	AQ_UK1_MACC	58	0.91	-0.20	0.20	-174.59	302.22	-0.18	0.31	388.63	0.66	0.09	0.55
PRECIP	AQ_UK2_HTAP	57	0.93	-0.17	0.14	-153.14	254.83	-0.16	0.26	336.58	0.71	0.24	0.62

Variable	Model	n	FAC2	FB	NMSE	MB	MGE	NMB	NMGE	RMSE	r	COE	IOA
PRECIP	ED_CHIM	56	0.91	-0.08	0.17	-71.14	270.52	-0.07	0.28	382.92	0.50	0.19	0.59
PRECIP	ED_CMAQ	56	0.96	-0.02	0.12	-21.57	247.43	-0.02	0.26	326.47	0.65	0.26	0.63
PRECIP	ED_EMEP	56	0.91	-0.08	0.17	-71.14	270.52	-0.07	0.28	382.92	0.50	0.19	0.59
PRECIP	ED_LOTO	56	1.00	-0.08	0.11	-72.56	229.08	-0.07	0.24	306.88	0.72	0.31	0.66
PRECIP	ED_MATCH	56	0.98	0.03	0.09	29.91	215.48	0.03	0.22	298.74	0.74	0.35	0.68
PRECIP	ED_MINNI	56	0.91	-0.08	0.17	-71.14	270.52	-0.07	0.28	382.92	0.50	0.19	0.59
TNO3_N	AQ_DE1_HTAP	46	0.52	-0.70	0.97	-0.25	0.26	-0.52	0.53	0.33	0.68	-0.11	0.44
TNO3_N	AQ_DK1_HTAP	45	0.82	0.28	0.19	0.16	0.19	0.33	0.40	0.24	0.79	0.18	0.59
TNO3_N	AQ_FI1_MACC	46	0.89	-0.05	0.23	-0.02	0.17	-0.05	0.36	0.22	0.63	0.26	0.63
TNO3_N	AQ_FI1_HTAP	46	0.80	0.23	0.22	0.13	0.19	0.26	0.40	0.25	0.69	0.18	0.59
TNO3_N	AQ_FRES1_HTAP	46	0.93	-0.13	0.13	-0.06	0.12	-0.12	0.26	0.16	0.85	0.46	0.73
TNO3_N	AQ_UK1_MACC	45	0.96	0.21	0.18	0.11	0.17	0.24	0.36	0.23	0.79	0.27	0.64
TNO3_N	AQ_UK2_HTAP	45	0.91	0.17	0.19	0.09	0.16	0.19	0.34	0.23	0.78	0.30	0.65
TNO3_N	ED_CHIM	45	0.67	0.54	0.52	0.35	0.35	0.73	0.74	0.45	0.79	-0.51	0.25
TNO3_N	ED_CMAQ	45	0.44	0.66	0.75	0.47	0.47	0.98	0.98	0.58	0.79	-1.01	0.00
TNO3_N	ED_EMEP	45	0.89	0.24	0.19	0.13	0.19	0.28	0.39	0.23	0.77	0.20	0.60
TNO3_N	ED_LOTO	45	0.89	0.17	0.15	0.09	0.15	0.19	0.30	0.20	0.82	0.38	0.69
TNO3_N	ED_MATCH	45	0.91	0.06	0.11	0.03	0.12	0.06	0.26	0.17	0.82	0.48	0.74
TNO3_N	ED_MINNI	45	0.91	0.20	0.16	0.11	0.17	0.22	0.35	0.22	0.80	0.29	0.65
TNO3_N	ENSEMBLE	45	0.87	0.23	0.15	0.13	0.17	0.26	0.35	0.21	0.82	0.29	0.64
TNH4_N	AQ_DE1_HTAP	40	0.78	0.10	0.46	0.15	0.69	0.11	0.48	1.02	0.48	0.07	0.54
TNH4_N	AQ_DK1_HTAP	39	0.87	0.25	0.27	0.40	0.60	0.28	0.42	0.84	0.80	0.21	0.60
TNH4_N	AQ_FI1_MACC	40	0.85	0.19	0.54	0.31	0.69	0.22	0.48	1.15	0.62	0.07	0.54
TNH4_N	AQ_FI1_HTAP	40	0.88	0.23	0.51	0.36	0.66	0.26	0.46	1.14	0.64	0.11	0.56
TNH4_N	AQ_FRES1_HTAP	40	0.83	0.33	0.44	0.56	0.73	0.39	0.52	1.11	0.78	0.01	0.50
TNH4_N	AQ_UK1_MACC	39	0.87	0.17	0.33	0.27	0.59	0.19	0.41	0.88	0.72	0.22	0.61
TNH4_N	AQ_UK2_HTAP	39	0.56	0.67	1.20	1.45	1.53	1.02	1.08	2.21	0.86	-1.02	-0.01

Variable	Model	n	FAC2	FB	NMSE	MB	MGE	NMB	NMGE	RMSE	r	COE	IOA
TNH4_N	ED_CHIM	39	0.82	0.25	0.27	0.40	0.59	0.28	0.41	0.84	0.79	0.23	0.61
TNH4_N	ED_CMAQ	39	0.72	0.37	0.51	0.64	0.84	0.45	0.59	1.22	0.68	-0.11	0.44
TNH4_N	ED_EMEP	39	0.90	0.03	0.20	0.04	0.48	0.03	0.34	0.64	0.81	0.37	0.68
TNH4_N	ED_LOTO	39	0.74	0.36	0.50	0.62	0.72	0.44	0.51	1.20	0.76	0.05	0.52
TNH4_N	ED_MATCH	39	0.82	-0.08	0.23	-0.12	0.48	-0.08	0.34	0.66	0.80	0.36	0.68
TNH4_N	ED_MINNI	39	0.85	0.25	0.26	0.40	0.57	0.28	0.40	0.81	0.79	0.25	0.63
TNH4_N	ENSEMBLE	39	0.85	0.19	0.26	0.29	0.55	0.21	0.38	0.79	0.78	0.28	0.64
TSO4_S	AQ_DE1_HTAP	20	0.75	0.32	0.49	0.30	0.44	0.39	0.57	0.64	0.73	-0.47	0.27
TSO4_S	AQ_DK1_HTAP	18	0.94	0.32	0.26	0.30	0.33	0.38	0.41	0.47	0.89	-0.13	0.44
TSO4_S	AQ_FI1_MACC	20	0.85	0.14	0.28	0.12	0.31	0.16	0.41	0.44	0.68	-0.04	0.48
TSO4_S	AQ_FI1_HTAP	20	0.90	0.02	0.13	0.02	0.19	0.02	0.24	0.29	0.78	0.37	0.69
TSO4_S	AQ_FRES1_HTAP	20	0.90	0.04	0.17	0.03	0.23	0.04	0.30	0.33	0.70	0.24	0.62
TSO4_S	AQ_UK1_MACC	19	0.89	0.23	0.25	0.20	0.27	0.26	0.36	0.42	0.71	0.11	0.56
TSO4_S	AQ_UK2_HTAP	19	0.84	0.31	0.36	0.28	0.40	0.37	0.53	0.53	0.78	-0.33	0.34
TSO4_S	ED_CHIM	18	0.89	-0.02	0.24	-0.02	0.23	-0.02	0.29	0.38	0.81	0.20	0.60
TSO4_S	ED_CMAQ	18	0.78	0.49	0.56	0.51	0.56	0.65	0.71	0.75	0.86	-0.93	0.04
TSO4_S	ED_EMEP	18	0.89	-0.16	0.18	-0.12	0.22	-0.15	0.28	0.31	0.85	0.24	0.62
TSO4_S	ED_LOTO	18	0.89	-0.11	0.16	-0.08	0.21	-0.10	0.27	0.30	0.84	0.27	0.64
TSO4_S	ED_MATCH	18	0.89	-0.12	0.14	-0.09	0.22	-0.11	0.28	0.28	0.86	0.25	0.62
TSO4_S	ED_MINNI	18	0.89	0.17	0.25	0.14	0.31	0.18	0.40	0.43	0.85	-0.08	0.46
TSO4_S	ENSEMBLE	18	0.89	-0.06	0.10	-0.04	0.19	-0.06	0.24	0.24	0.90	0.34	0.67

S3.8 . Tables with complete statistics from annual values (definition in S3.10). Common sites

Variable	Model	n	FAC2	FB	NMSE	MB	MGE	NMB	NMGE	RMSE	r	COE	IOA
WNO3_N	AQ_DE1_HTAP	10	0.00	-1.50	6.99	-182.27	182.27	-0.86	0.86	212.33	0.73	-0.93	0.04
WNO3_N	AQ_DK1_HTAP	10	1.00	0.05	0.06	10.85	44.58	0.05	0.21	55.45	0.91	0.53	0.76
WNO3_N	AQ_FI1_MACC	10	0.60	-0.50	0.44	-85.55	88.51	-0.40	0.42	109.07	0.85	0.06	0.53
WNO3_N	AQ_FI1_HTAP	10	0.90	-0.12	0.13	-24.26	53.33	-0.11	0.25	71.96	0.84	0.44	0.72
WNO3_N	AQ_FRES1_HTAP	10	1.00	-0.35	0.26	-62.88	65.40	-0.30	0.31	91.18	0.87	0.31	0.65
WNO3_N	AQ_UK1_MACC	10	0.50	-0.48	0.60	-81.64	110.23	-0.38	0.52	129.09	0.58	-0.17	0.42
WNO3_N	AQ_UK2_HTAP	10	0.80	-0.33	0.29	-59.79	75.59	-0.28	0.36	96.92	0.77	0.20	0.60
WNO3_N	ED_CHIM	10	1.00	-0.26	0.18	-48.95	50.57	-0.23	0.24	79.37	0.88	0.47	0.73
WNO3_N	ED_CMAQ	10	1.00	-0.16	0.15	-31.91	61.28	-0.15	0.29	75.29	0.82	0.35	0.68
WNO3_N	ED_EMEP	10	1.00	0.11	0.12	23.99	61.58	0.11	0.29	78.24	0.86	0.35	0.67
WNO3_N	ED_LOTO	10	0.80	-0.27	0.17	-50.93	60.31	-0.24	0.28	75.66	0.88	0.36	0.68
WNO3_N	ED_MATCH	10	1.00	0.21	0.21	48.61	80.02	0.23	0.38	107.57	0.84	0.15	0.58
WNO3_N	ED_MINNI	10	0.00	-1.12	2.57	-153.06	153.06	-0.72	0.72	180.23	0.82	-0.62	0.19
WNO3_N	ENSEMBLE	10	1.00	-0.09	0.08	-18.04	40.45	-0.08	0.19	56.60	0.89	0.57	0.79
HNO3_N	AQ_DE1_HTAP	10	0.10	-1.27	5.94	-0.13	0.13	-0.78	0.82	0.19	0.29	-0.07	0.46
HNO3_N	AQ_DK1_HTAP	10	0.60	-0.49	1.19	-0.06	0.09	-0.40	0.55	0.14	0.53	0.27	0.64
HNO3_N	AQ_FI1_MACC	10	0.40	-0.95	3.02	-0.11	0.12	-0.65	0.71	0.17	0.50	0.06	0.53
HNO3_N	AQ_FI1_HTAP	10	0.60	-0.60	1.50	-0.08	0.10	-0.46	0.61	0.15	0.52	0.20	0.60
HNO3_N	AQ_FRES1_HTAP	10	0.50	-0.67	1.86	-0.08	0.10	-0.50	0.62	0.16	0.38	0.18	0.59
HNO3_N	AQ_UK1_MACC	10	0.70	-0.39	1.08	-0.05	0.09	-0.32	0.56	0.14	0.44	0.27	0.64
HNO3_N	AQ_UK2_HTAP	10	0.40	-0.86	2.56	-0.10	0.11	-0.60	0.69	0.17	0.40	0.09	0.55
HNO3_N	ED_CHIM	10	0.50	-0.31	1.13	-0.04	0.10	-0.27	0.62	0.15	0.31	0.19	0.59
HNO3_N	ED_CMAQ	10	0.60	-0.18	0.84	-0.03	0.09	-0.16	0.56	0.14	0.41	0.26	0.63
HNO3_N	ED_EMEP	10	0.30	-0.83	2.29	-0.10	0.12	-0.59	0.72	0.16	0.49	0.05	0.53
HNO3_N	ED_LOTO	10	0.50	-0.63	1.60	-0.08	0.11	-0.48	0.65	0.15	0.47	0.14	0.57

Variable	Model	n	FAC2	FB	NMSE	MB	MGE	NMB	NMGE	RMSE	r	COE	IOA
HNO3_N	ED_MATCH	10	0.60	-0.36	1.19	-0.05	0.11	-0.30	0.69	0.15	0.27	0.10	0.55
HNO3_N	ED_MINNI	10	0.40	-0.66	1.91	-0.08	0.11	-0.50	0.69	0.16	0.29	0.10	0.55
HNO3_N	ENSEMBLE	10	0.60	-0.58	1.50	-0.07	0.10	-0.45	0.61	0.15	0.45	0.20	0.60
PM_NO3_N	AQ_DE1_HTAP	10	0.70	-0.46	0.64	-0.13	0.14	-0.37	0.39	0.22	0.67	0.26	0.63
PM_NO3_N	AQ_DK1_HTAP	10	0.80	0.44	0.34	0.20	0.22	0.56	0.61	0.26	0.79	-0.18	0.41
PM_NO3_N	AQ_FI1_MACC	10	0.90	-0.12	0.23	-0.04	0.11	-0.12	0.31	0.16	0.76	0.39	0.70
PM_NO3_N	AQ_FI1_HTAP	10	0.90	0.18	0.14	0.07	0.11	0.19	0.31	0.15	0.84	0.41	0.70
PM_NO3_N	AQ_FRES1_HTAP	10	0.70	0.18	0.16	0.07	0.12	0.20	0.34	0.15	0.85	0.34	0.67
PM_NO3_N	AQ_UK1_MACC	10	0.70	0.30	0.32	0.12	0.18	0.35	0.50	0.23	0.70	0.03	0.52
PM_NO3_N	AQ_UK2_HTAP	10	0.60	0.36	0.53	0.15	0.24	0.44	0.69	0.31	0.55	-0.33	0.34
PM_NO3_N	ED_CHIM	10	0.50	0.68	0.79	0.36	0.37	1.03	1.04	0.45	0.71	-1.01	0.00
PM_NO3_N	ED_CMAQ	10	0.40	0.77	1.03	0.44	0.45	1.26	1.26	0.54	0.78	-1.43	-0.18
PM_NO3_N	ED_EMEP	10	0.70	0.34	0.31	0.14	0.19	0.41	0.53	0.23	0.70	-0.03	0.48
PM_NO3_N	ED_LOTO	10	0.90	0.45	0.35	0.20	0.21	0.58	0.59	0.26	0.93	-0.14	0.43
PM_NO3_N	ED_MATCH	10	0.90	0.08	0.21	0.03	0.12	0.09	0.35	0.17	0.72	0.33	0.66
PM_NO3_N	ED_MINNI	10	0.70	0.41	0.35	0.18	0.21	0.52	0.59	0.26	0.76	-0.14	0.43
PM_NO3_N	ENSEMBLE	10	0.80	0.33	0.23	0.14	0.17	0.39	0.47	0.20	0.82	0.09	0.55
WNH4_N	AQ_DE1_HTAP	9	0.44	-0.76	1.06	-170.51	170.51	-0.55	0.55	215.16	0.55	-0.55	0.23
WNH4_N	AQ_DK1_HTAP	9	0.89	0.18	0.30	63.05	137.57	0.20	0.44	186.71	0.82	-0.25	0.38
WNH4_N	AQ_FI1_MACC	9	0.89	0.05	0.22	14.57	113.54	0.05	0.37	149.34	0.83	-0.03	0.48
WNH4_N	AQ_FI1_HTAP	9	0.89	0.07	0.24	22.81	118.26	0.07	0.38	158.39	0.82	-0.07	0.46
WNH4_N	AQ_FRES1_HTAP	9	0.33	-0.55	0.52	-133.56	141.45	-0.43	0.46	169.61	0.79	-0.28	0.36
WNH4_N	AQ_UK1_MACC	9	0.67	-0.43	0.51	-110.16	159.01	-0.35	0.51	177.79	0.55	-0.44	0.28
WNH4_N	AQ_UK2_HTAP	9	0.89	-0.12	0.22	-35.30	102.05	-0.11	0.33	137.08	0.67	0.07	0.54
WNH4_N	ED_CHIM	9	0.78	-0.13	0.23	-37.29	117.57	-0.12	0.38	140.77	0.66	-0.07	0.47
WNH4_N	ED_CMAQ	9	0.78	-0.40	0.29	-102.82	103.72	-0.33	0.33	135.97	0.80	0.06	0.53
WNH4_N	ED_EMEP	9	0.89	0.23	0.22	82.66	135.40	0.27	0.44	165.63	0.81	-0.23	0.39

Variable	Model	n	FAC2	FB	NMSE	MB	MGE	NMB	NMGE	RMSE	r	COE	IOA
WNH4_N	ED_LOTO	9	0.89	-0.07	0.19	-20.34	111.97	-0.07	0.36	132.33	0.82	-0.02	0.49
WNH4_N	ED_MATCH	9	0.78	0.38	0.61	146.90	191.90	0.47	0.62	295.22	0.83	-0.74	0.13
WNH4_N	ED_MINNI	9	0.56	-0.63	0.65	-149.29	149.29	-0.48	0.48	181.22	0.81	-0.36	0.32
WNH4_N	ENSEMBLE	9	0.89	0.12	0.23	38.91	117.59	0.13	0.38	158.33	0.82	-0.07	0.47
NH3_N	AQ_DE1_HTAP	9	0.44	0.57	0.76	0.62	0.77	0.79	0.99	0.91	0.15	-0.85	0.08
NH3_N	AQ_DK1_HTAP	9	0.67	0.64	0.88	0.74	0.74	0.94	0.94	1.02	0.80	-0.77	0.11
NH3_N	AQ_FI1_MACC	9	0.44	0.94	2.98	1.38	1.38	1.77	1.77	2.24	0.73	-2.31	-0.40
NH3_N	AQ_FI1_HTAP	9	0.56	0.93	2.88	1.37	1.37	1.75	1.75	2.19	0.74	-2.28	-0.39
NH3_N	AQ_FRES1_HTAP	9	0.56	0.77	1.71	0.97	0.97	1.24	1.24	1.53	0.77	-1.33	-0.14
NH3_N	AQ_UK1_MACC	9	0.78	0.59	1.32	0.66	0.67	0.85	0.86	1.22	0.74	-0.61	0.19
NH3_N	AQ_UK2_HTAP	9	0.44	1.02	2.68	1.62	1.63	2.08	2.09	2.24	0.83	-2.92	-0.49
NH3_N	ED_CHIM	9	0.78	0.37	0.26	0.35	0.40	0.45	0.51	0.48	0.91	0.05	0.52
NH3_N	ED_CMAQ	9	0.67	0.52	1.21	0.55	0.60	0.71	0.77	1.12	0.46	-0.44	0.28
NH3_N	ED_EMEP	9	0.89	0.19	0.14	0.16	0.25	0.21	0.32	0.32	0.89	0.40	0.70
NH3_N	ED_LOTO	9	0.44	0.84	1.86	1.12	1.12	1.44	1.44	1.66	0.89	-1.69	-0.26
NH3_N	ED_MATCH	9	0.67	0.29	0.36	0.26	0.37	0.34	0.48	0.54	0.73	0.10	0.55
NH3_N	ED_MINNI	9	0.56	0.49	0.41	0.51	0.53	0.65	0.67	0.64	0.85	-0.26	0.37
NH3_N	ENSEMBLE	9	0.67	0.66	1.06	0.77	0.77	0.98	0.98	1.13	0.82	-0.85	0.08
PM_NH4_N	AQ_DE1_HTAP	9	1.00	-0.17	0.15	-0.13	0.24	-0.16	0.29	0.30	0.74	0.27	0.63
PM_NH4_N	AQ_DK1_HTAP	9	0.67	0.11	0.12	0.09	0.28	0.11	0.33	0.31	0.67	0.16	0.58
PM_NH4_N	AQ_FI1_MACC	9	0.78	-0.27	0.27	-0.20	0.27	-0.24	0.32	0.38	0.58	0.20	0.60
PM_NH4_N	AQ_FI1_HTAP	9	1.00	-0.14	0.14	-0.11	0.24	-0.13	0.28	0.29	0.74	0.29	0.64
PM_NH4_N	AQ_FRES1_HTAP	9	0.67	0.27	0.16	0.26	0.32	0.32	0.39	0.39	0.70	0.03	0.52
PM_NH4_N	AQ_UK1_MACC	9	0.78	0.17	0.15	0.15	0.28	0.19	0.34	0.35	0.67	0.16	0.58
PM_NH4_N	AQ_UK2_HTAP	9	0.67	0.24	0.27	0.23	0.42	0.28	0.51	0.49	0.50	-0.27	0.36
PM_NH4_N	ED_CHIM	9	0.67	0.40	0.27	0.42	0.43	0.51	0.52	0.53	0.54	-0.30	0.35
PM_NH4_N	ED_CMAQ	9	0.56	0.55	0.40	0.64	0.64	0.77	0.77	0.70	0.80	-0.92	0.04

Variable	Model	n	FAC2	FB	NMSE	MB	MGE	NMB	NMGE	RMSE	r	COE	IOA
PM_NH4_N	ED_EMEP	9	0.78	0.07	0.15	0.06	0.29	0.07	0.35	0.34	0.55	0.12	0.56
PM_NH4_N	ED_LOTO	9	0.67	0.18	0.14	0.17	0.29	0.20	0.35	0.34	0.70	0.14	0.57
PM_NH4_N	ED_MATCH	9	0.67	-0.16	0.24	-0.12	0.34	-0.15	0.41	0.38	0.40	-0.03	0.49
PM_NH4_N	ED_MINNI	9	0.67	0.29	0.20	0.28	0.35	0.34	0.42	0.43	0.59	-0.06	0.47
PM_NH4_N	ENSEMBLE	9	0.67	0.05	0.11	0.05	0.25	0.05	0.30	0.29	0.72	0.24	0.62
WSO4_S	AQ_DE1_HTAP	12	0.75	-0.23	0.24	-38.52	70.62	-0.21	0.38	81.29	0.80	0.22	0.61
WSO4_S	AQ_DK1_HTAP	11	0.55	-0.52	0.37	-78.17	78.17	-0.41	0.41	89.10	0.93	0.19	0.60
WSO4_S	AQ_FI1_MACC	12	0.75	-0.24	0.24	-39.46	71.22	-0.21	0.38	80.97	0.91	0.21	0.61
WSO4_S	AQ_FI1_HTAP	12	0.75	-0.30	0.23	-48.80	62.89	-0.26	0.34	76.74	0.91	0.31	0.65
WSO4_S	AQ_FRES1_HTAP	12	0.33	-0.69	0.67	-96.36	96.36	-0.51	0.51	106.94	0.95	-0.06	0.47
WSO4_S	AQ_UK1_MACC	12	0.75	0.11	0.23	22.26	67.19	0.12	0.36	94.49	0.70	0.26	0.63
WSO4_S	AQ_UK2_HTAP	12	0.75	0.42	0.35	99.31	112.18	0.53	0.60	137.54	0.86	-0.24	0.38
WSO4_S	ED_CHIM	11	0.27	-0.92	1.66	-120.24	120.24	-0.63	0.63	148.52	0.77	-0.24	0.38
WSO4_S	ED_CMAQ	11	0.91	0.15	0.14	30.22	63.91	0.16	0.34	75.67	0.83	0.34	0.67
WSO4_S	ED_EMEP	11	0.91	0.25	0.30	54.41	83.57	0.29	0.44	118.38	0.87	0.14	0.57
WSO4_S	ED_LOTO	11	0.82	-0.08	0.13	-15.37	53.06	-0.08	0.28	64.85	0.96	0.45	0.73
WSO4_S	ED_MATCH	11	0.55	0.62	1.06	172.30	176.94	0.91	0.93	270.30	0.95	-0.83	0.09
WSO4_S	ED_MINNI	11	0.27	-0.79	1.00	-107.94	109.69	-0.57	0.58	125.14	0.85	-0.13	0.43
WSO4_S	ENSEMBLE	11	0.91	0.13	0.22	25.68	67.68	0.13	0.36	94.15	0.94	0.30	0.65
SO2_S	AQ_DE1_HTAP	12	0.42	0.75	2.04	0.40	0.45	1.19	1.33	0.71	0.71	-1.48	-0.19
SO2_S	AQ_DK1_HTAP	11	0.91	0.23	0.26	0.09	0.14	0.26	0.40	0.21	0.87	0.20	0.60
SO2_S	AQ_FI1_MACC	12	0.58	0.62	1.04	0.30	0.33	0.91	0.99	0.47	0.56	-0.86	0.07
SO2_S	AQ_FI1_HTAP	12	0.83	0.43	0.58	0.18	0.21	0.55	0.63	0.32	0.64	-0.18	0.41
SO2_S	AQ_FRES1_HTAP	12	0.58	0.02	0.74	0.01	0.18	0.02	0.54	0.29	0.43	-0.01	0.49
SO2_S	AQ_UK1_MACC	12	0.75	0.59	1.12	0.28	0.31	0.85	0.92	0.48	0.52	-0.72	0.14
SO2_S	AQ_UK2_HTAP	12	0.33	0.70	1.37	0.36	0.40	1.08	1.18	0.57	0.72	-1.21	-0.10
SO2_S	ED_CHIM	11	0.55	0.35	1.30	0.15	0.29	0.43	0.83	0.49	0.69	-0.64	0.18

Variable	Model	n	FAC2	FB	NMSE	MB	MGE	NMB	NMGE	RMSE	r	COE	IOA
SO2_S	ED_CMAQ	11	0.27	0.76	2.14	0.44	0.50	1.24	1.40	0.78	0.73	-1.79	-0.28
SO2_S	ED_EMEP	11	0.36	-0.21	0.67	-0.07	0.21	-0.19	0.58	0.26	0.73	-0.14	0.43
SO2_S	ED_LOTO	11	0.64	0.21	0.77	0.08	0.20	0.24	0.55	0.35	0.75	-0.10	0.45
SO2_S	ED_MATCH	11	0.64	0.20	0.56	0.08	0.21	0.22	0.58	0.29	0.74	-0.16	0.42
SO2_S	ED_MINNI	11	0.36	0.57	1.17	0.28	0.37	0.80	1.03	0.52	0.73	-1.05	-0.02
SO2_S	ENSEMBLE	11	0.73	0.22	0.37	0.09	0.18	0.25	0.50	0.24	0.82	0.02	0.51
PM_SO4_S	AQ_DE1_HTAP	12	0.92	0.06	0.05	0.03	0.08	0.07	0.20	0.10	0.92	0.60	0.80
PM_SO4_S	AQ_DK1_HTAP	11	0.73	0.45	0.44	0.26	0.27	0.59	0.61	0.37	0.90	-0.42	0.29
PM_SO4_S	AQ_FI1_MACC	12	0.50	-0.32	0.23	-0.11	0.14	-0.28	0.34	0.17	0.86	0.31	0.66
PM_SO4_S	AQ_FI1_HTAP	12	0.50	-0.40	0.30	-0.14	0.16	-0.33	0.39	0.19	0.85	0.20	0.60
PM_SO4_S	AQ_FRES1_HTAP	12	0.92	0.17	0.09	0.08	0.11	0.18	0.26	0.13	0.92	0.46	0.73
PM_SO4_S	AQ_UK1_MACC	12	0.75	-0.01	0.07	0.00	0.08	-0.01	0.19	0.11	0.89	0.61	0.80
PM_SO4_S	AQ_UK2_HTAP	12	0.83	-0.05	0.08	-0.02	0.09	-0.05	0.22	0.11	0.89	0.55	0.77
PM_SO4_S	ED_CHIM	11	0.91	-0.29	0.17	-0.11	0.13	-0.25	0.30	0.16	0.89	0.30	0.65
PM_SO4_S	ED_CMAQ	11	0.73	0.24	0.12	0.12	0.14	0.27	0.32	0.18	0.83	0.26	0.63
PM_SO4_S	ED_EMEP	11	1.00	-0.05	0.08	-0.02	0.09	-0.05	0.19	0.12	0.91	0.55	0.78
PM_SO4_S	ED_LOTO	11	0.82	-0.30	0.22	-0.11	0.14	-0.26	0.31	0.18	0.80	0.27	0.64
PM_SO4_S	ED_MATCH	11	0.64	-0.44	0.30	-0.16	0.17	-0.36	0.38	0.19	0.89	0.12	0.56
PM_SO4_S	ED_MINNI	11	0.91	-0.17	0.08	-0.07	0.08	-0.16	0.19	0.11	0.93	0.56	0.78
PM_SO4_S	ENSEMBLE	11	0.91	-0.31	0.14	-0.12	0.12	-0.27	0.28	0.14	0.94	0.35	0.68

S3.9 Monthly values of WNO3_N, WNH4_N, WSO4_S and precipitation

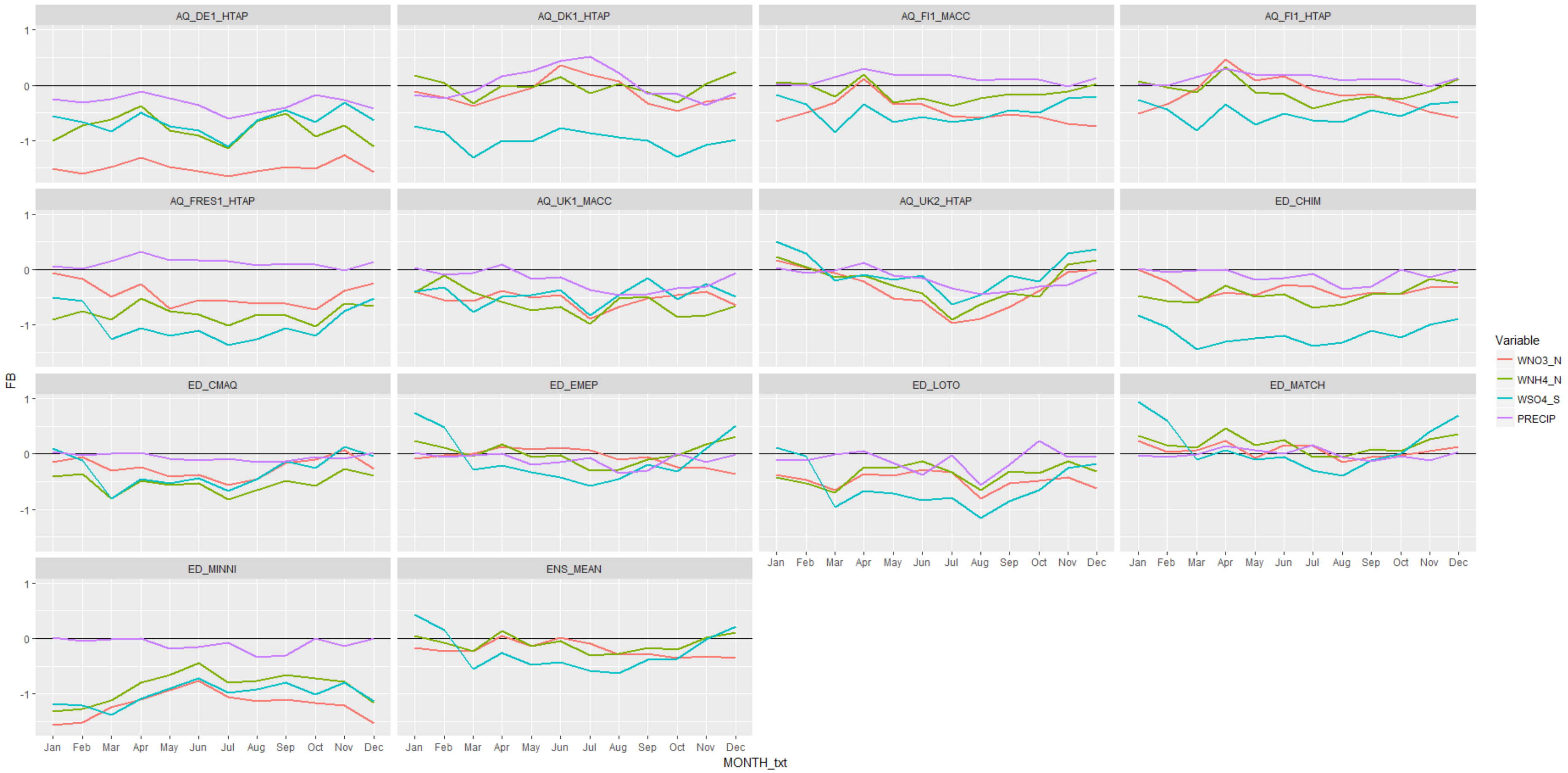

S3.10 Definition of statistical metrics in S3.6, 3.7 and 3.8

Mean Gross Error, MGE

$$\text{MGE} = \frac{1}{n}\sum_{1}^{n} |M - O|$$

Root Mean Square Error, RMSE

$$\text{RMSE} = \sqrt{\frac{\sum_{1}^{n} (M - O)^2}{n}}$$

Normalized Mean Bias, NMB

$$\text{NMB} = \frac{\sum_{1}^{n} (M - O)}{\sum_{1}^{n} (O)}$$

Normalized Mean Gross Error, NMGE

$$\text{NMGE} = \frac{\sum_{1}^{n} |M - O|}{\sum_{1}^{n} (O)}$$

Correlation Coefficient, r

$$\text{r} = \frac{1}{(n-1)} \sum_{1}^{n} \left(\left(\frac{O - \overline{O}}{\sigma_o} \right) * \left(\frac{M - \overline{M}}{\sigma_m} \right) \right)$$

Index of Agreement, IOA

$$\text{IOA} = 1 - \left[\frac{\sum_{1}^{n} (O - M)^2}{\sum_{1}^{n} \left(|M - \overline{O}| + |O - \overline{O}| \right)^2} \right]$$

S4. Gas/particle contribution

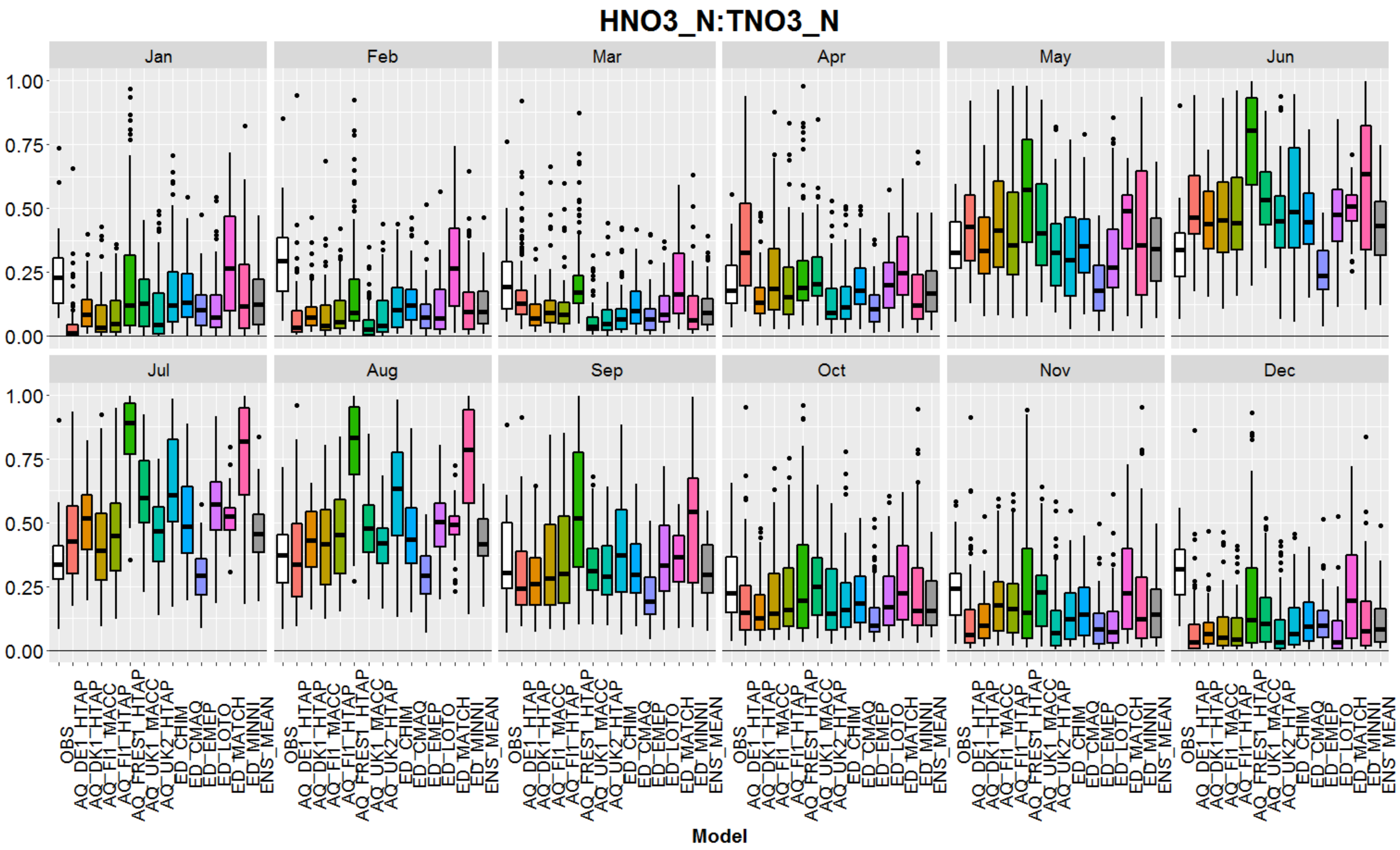

HNO3_N:TNO3_N
Jan
Feb
Mar
Apr
May
Jun
Jul
Aug
Sep
Oct
Nov
Dec
1.00
0.75
0.50
0.25
0.00
OBS
AQ_DE1_HTAP
AQ_DK1_HTAP
AQ_FI1_MACC
AQ_FI1_HTAP
AQ_FRES1_HTAP
AQ_UK1_MACC
AQ_UK2_HTAP
ED_CHIM
ED_CMAQ
ED_EMEP
ED_LOTO
ED_MATCH
ED_MINNI
ENS_MEAN
Model

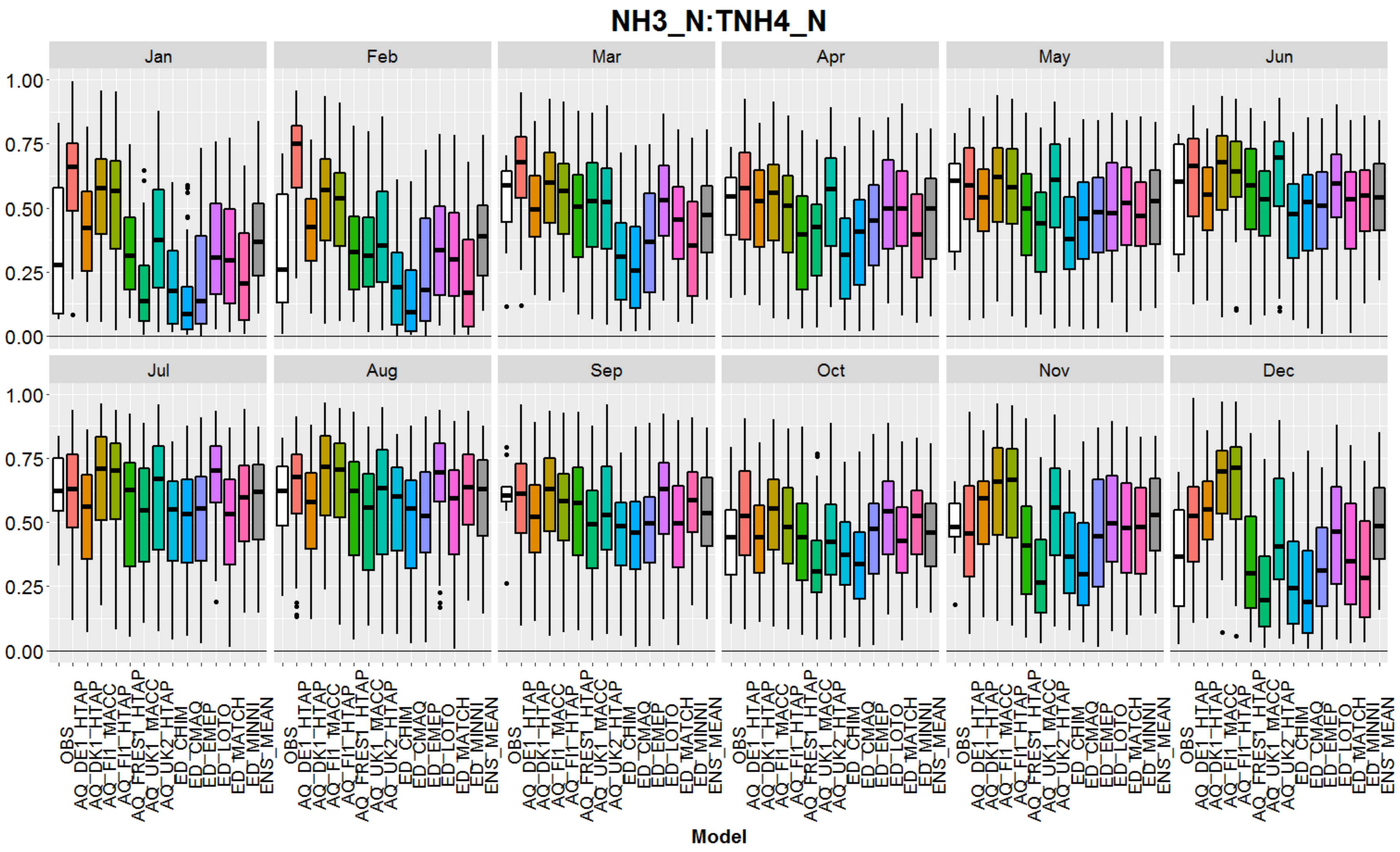

NH3_N:TNH4_N
Jan
Feb
Mar
Apr
May
Jun
Jul
Aug
Sep
Oct
Nov
Dec
1.00
0.75
0.50
0.25
0.00
OBS
AQ_DE1_HTAP
AQ_DK1_HTAP
AQ_FI1_MACC
AQ_FI1_HTAP
AQ_FRES1_HTAP
AQ_UK1_MACC
AQ_UK2_HTAP
ED_CHIM
ED_CMAQ
ED_EMEP
ED_LOTO
ED_MATCH
ED_MINNI
ENS_MEAN
Model

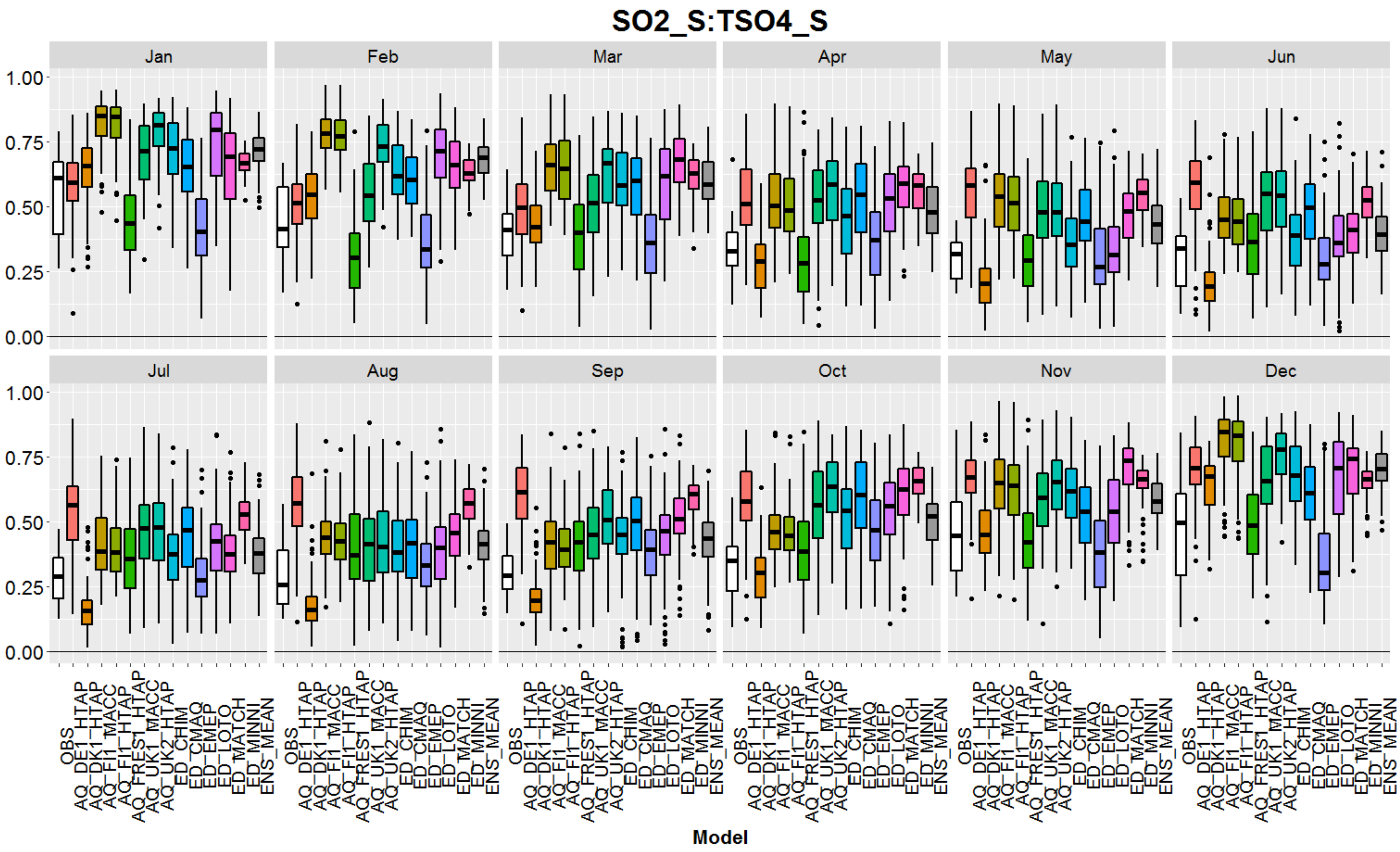

SO2_S:TSO4_S
Jan
Feb
Mar
Apr
May
Jun
Jul
Aug
Sep
Oct
Nov
Dec
1.00
0.75
0.50
0.25
0.00
OBS
AQ_DE1_HTAP
AQ_DK1_HTAP
AQ_FI1_MACC
AQ_FI1_HTAP
AQ_FREST_HTAP
AQ_UK1_MACC
AQ_UK2_HTAP
ED_CHIM
ED_CMAQ
ED_EMEP
ED_LOTO
ED_MATCH
ED_MINNI
ENS_MEAN
Model

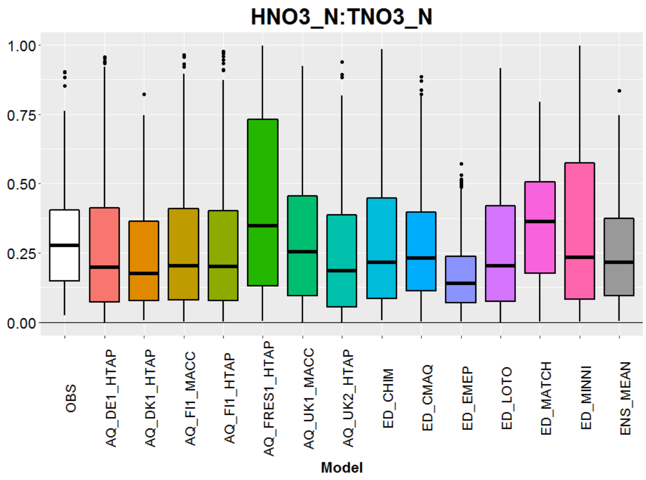
HNO3_N:TNO3_N
1.00
0.75
0.50
0.25
0.00
OBS
AQ_DE1_HTAP
AQ_DK1_HTAP
AQ_FI1_MACC
AQ_FI1_HTAP
AQ_FRES1_HTAP
AQ_UK1_MACC
AQ_UK2_HTAP
ED_CHIM
ED_CMAQ
ED_EMEP
ED_LOTO
ED_MATCH
ED_MINNI
ENS_MEAN
Model

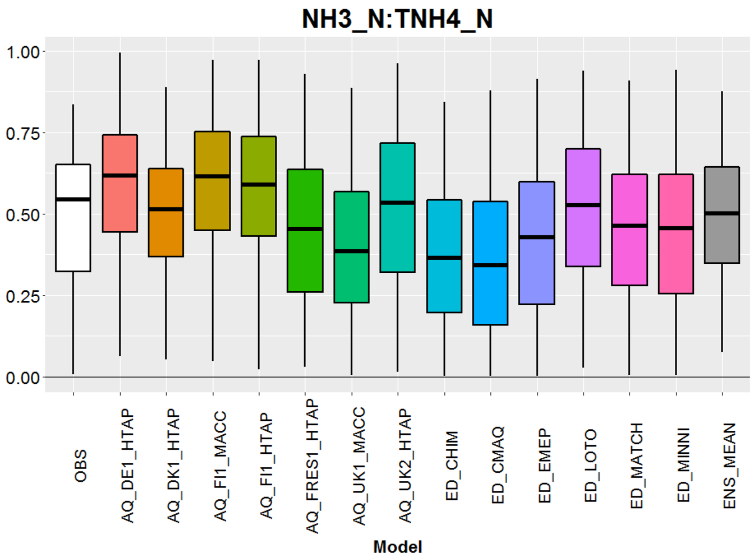
NH3_N:TNH4_N
1.00
0.75
0.50
0.25
0.00
OBS
AQ_DE1_HTAP
AQ_DK1_HTAP
AQ_FI1_MACC
AQ_FI1_HTAP
AQ_FRES1_HTAP
AQ_UK1_MACC
AQ_UK2_HTAP
ED_CHIM
ED_CMAQ
ED_EMEP
ED_LOTO
ED_MATCH
ED_MINNI
ENS_MEAN
Model

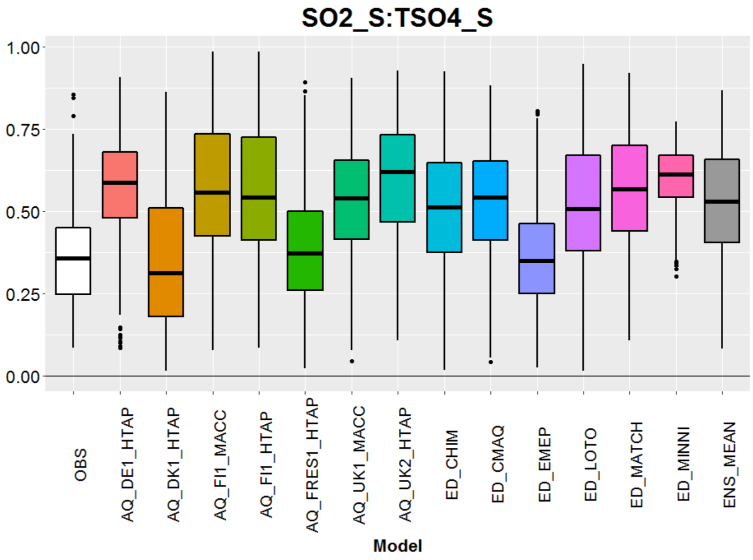
SO2_S:TSO4_S
1.00
0.75
0.50
0.25
0.00
OBS
AQ_DE1_HTAP
AQ_DK1_HTAP
AQ_FI1_MACC
AQ_FI1_HTAP
AQ_FRES1_HTAP
AQ_UK1_MACC
AQ_UK2_HTAP
ED_CHIM
ED_CMAQ
ED_EMEP
ED_LOTO
ED_MATCH
ED_MINNI
ENS_MEAN
Model

S5. EFFECT ON WET DEPOSTION IN EUROPE OF A REDUCTION OF A 20% OF EMISSIONS 1) AT A GLOBAL SCALE, (GLO), 2) IN EUROPE (EUR) AND 3) IN NORTH AMERICA (NAM)

S5.1 EFFECT ON WET DEPOSTION IN EUROPE OF A REDUCTION OF A 20% OF GLOBAL EMISSIONS (GLO) FOR WNH4_N (left), WNO3_N (middle) and WSO4_S (right), ACCORDING TO AQ_FI1_MACC

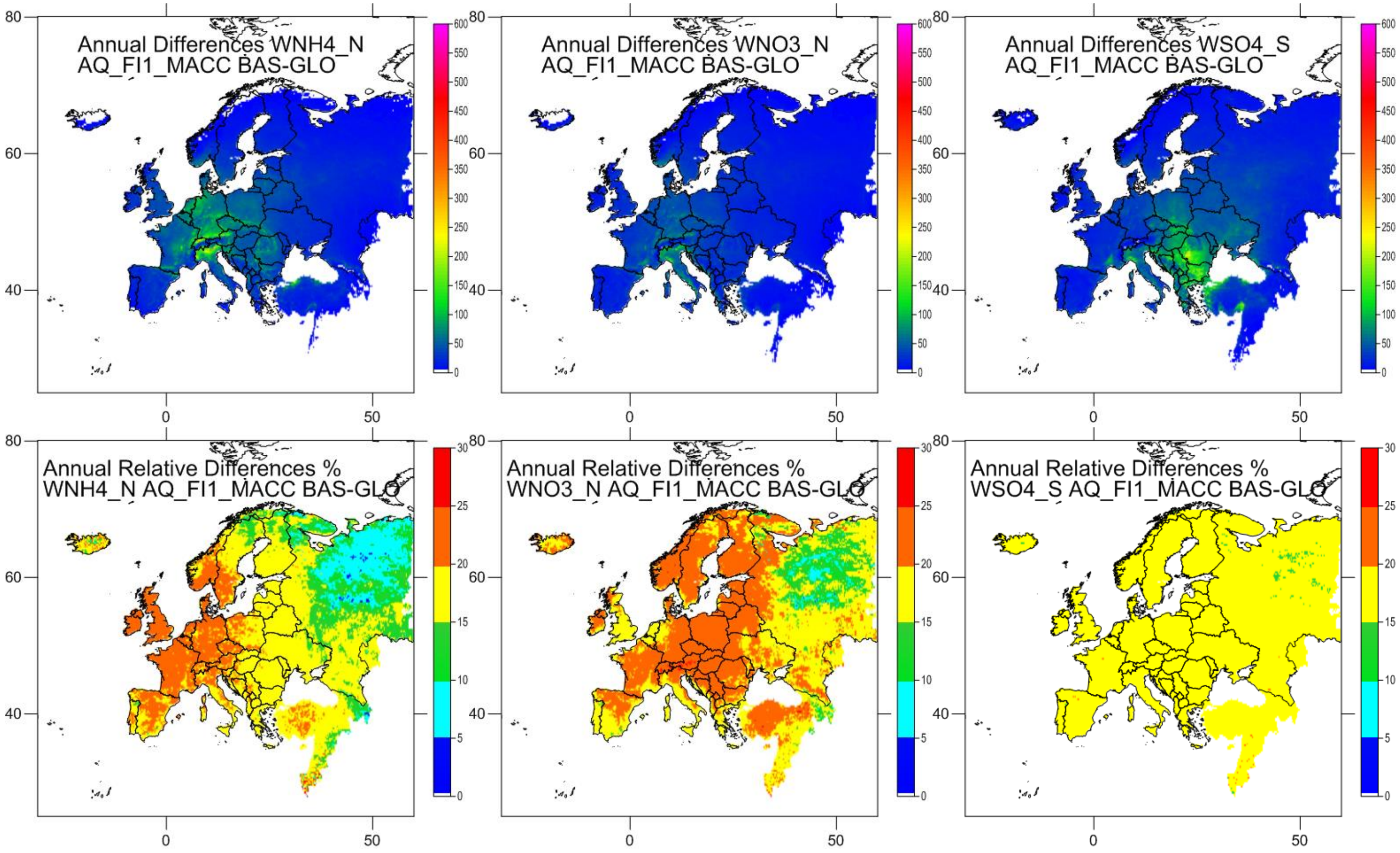

S5.2 EFFECT ON WET DEPOSTION IN EUROPE OF A REDUCTION OF A 20% OF EUROPEAN EMISSIONS (EUR) FOR WNH4_N (left), WNO3_N (middle) and WSO4_S (right), ACCORDING TO AQ_FI1_MACC

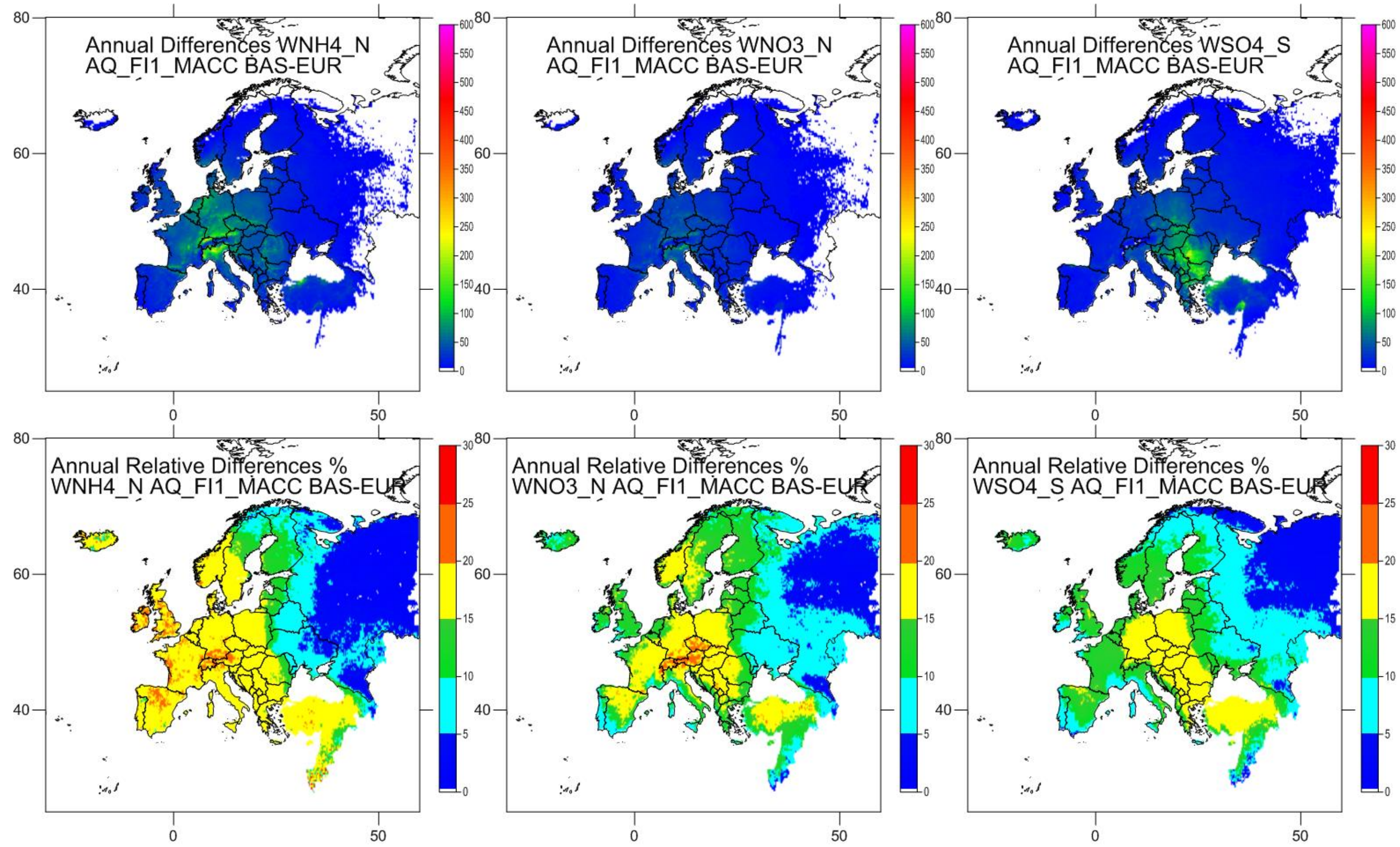

S5.3. EFFECT ON WET DEPOSTION IN EUROPE OF A REDUCTION OF A 20% OF NORTH-AMERICAN EMISSIONS (NAM) FOR WNH4_N (left), WNO3_N (middle) and WSO4_S (right), ACCORDING TO AQ_FI1_MACC

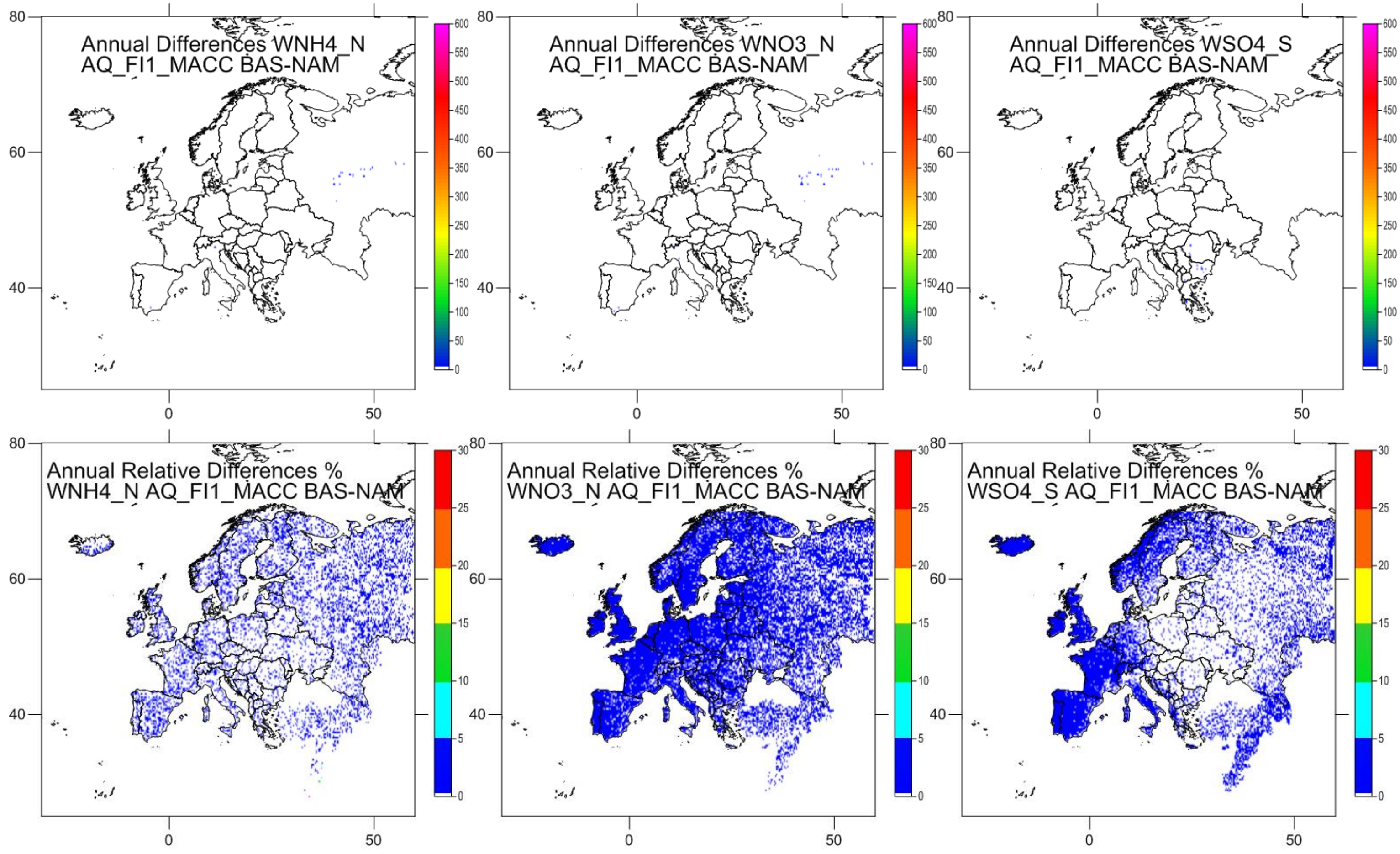

S5.4 . Effect on Europe of the reduction of 20% of emissions at a global scale (left maps), in Europe (middle) and in North America (right) for WNO3_N, according to HT_FRES1_HTAP in terms of wet deposition

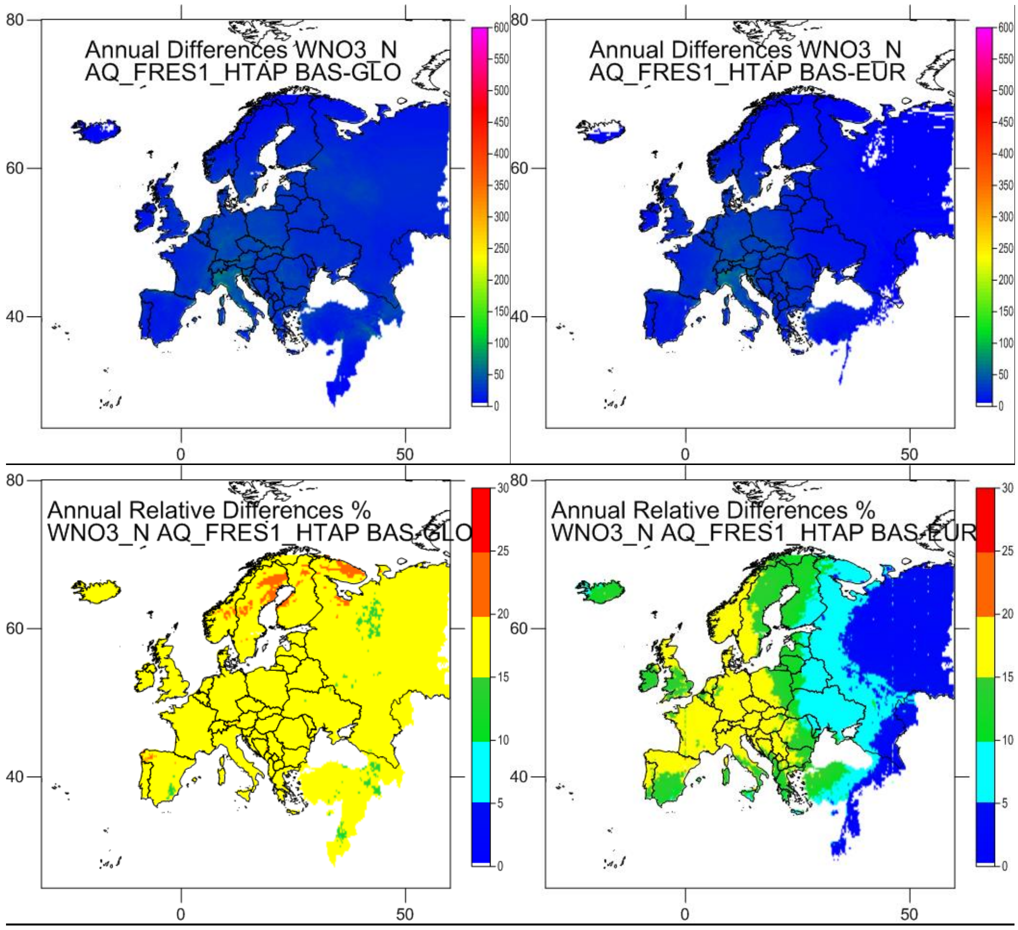

S6. EFFECT ON DRY DEPOSTION IN EUROPE OF A REDUCTION OF A 20% OF EMISSIONS 1) AT A GLOBAL SCALE, (GLO), 2) IN EUROPE (EUR) AND 3) IN NORTH AMERICA (NAM)

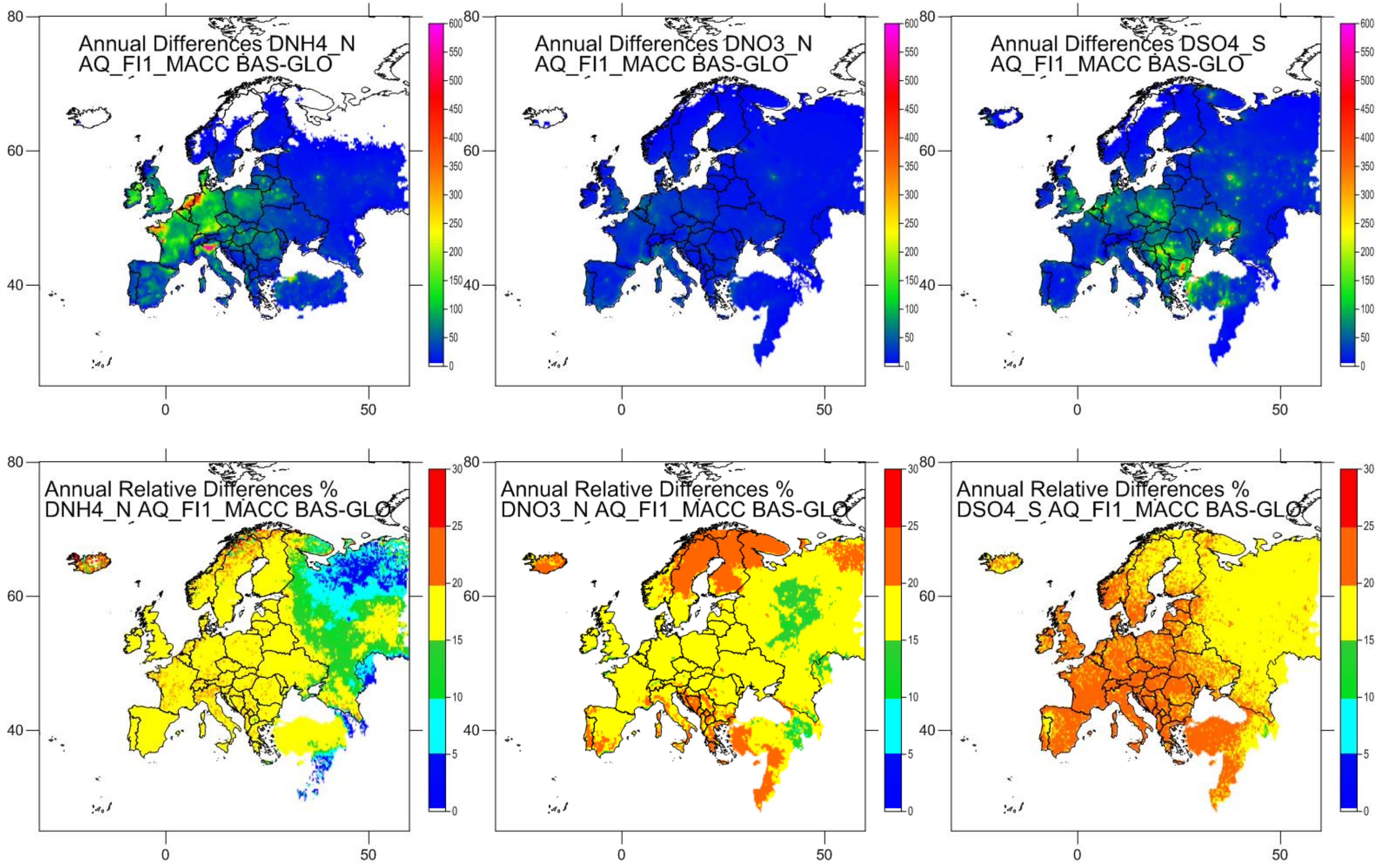
Annual Differences DNH4_N
AQ_FI1_MACC BAS-GLO
Annual Differences DNO3_N
AQ_FI1_MACC BAS-GLO
Annual Differences DSO4_S
AQ_FI1_MACC BAS-GLO
Annual Relative Differences %
DNH4_N AQ_FI1_MACC BAS-GLO
Annual Relative Differences %
DNO3_N AQ_FI1_MACC BAS-GLO
Annual Relative Differences %
DSO4_S AQ_FI1_MACC BAS-GLO
80
60
40
0
50
600
550
500
450
400
350
300
250
200
150
100
50
0
30
25
20
15
10
5

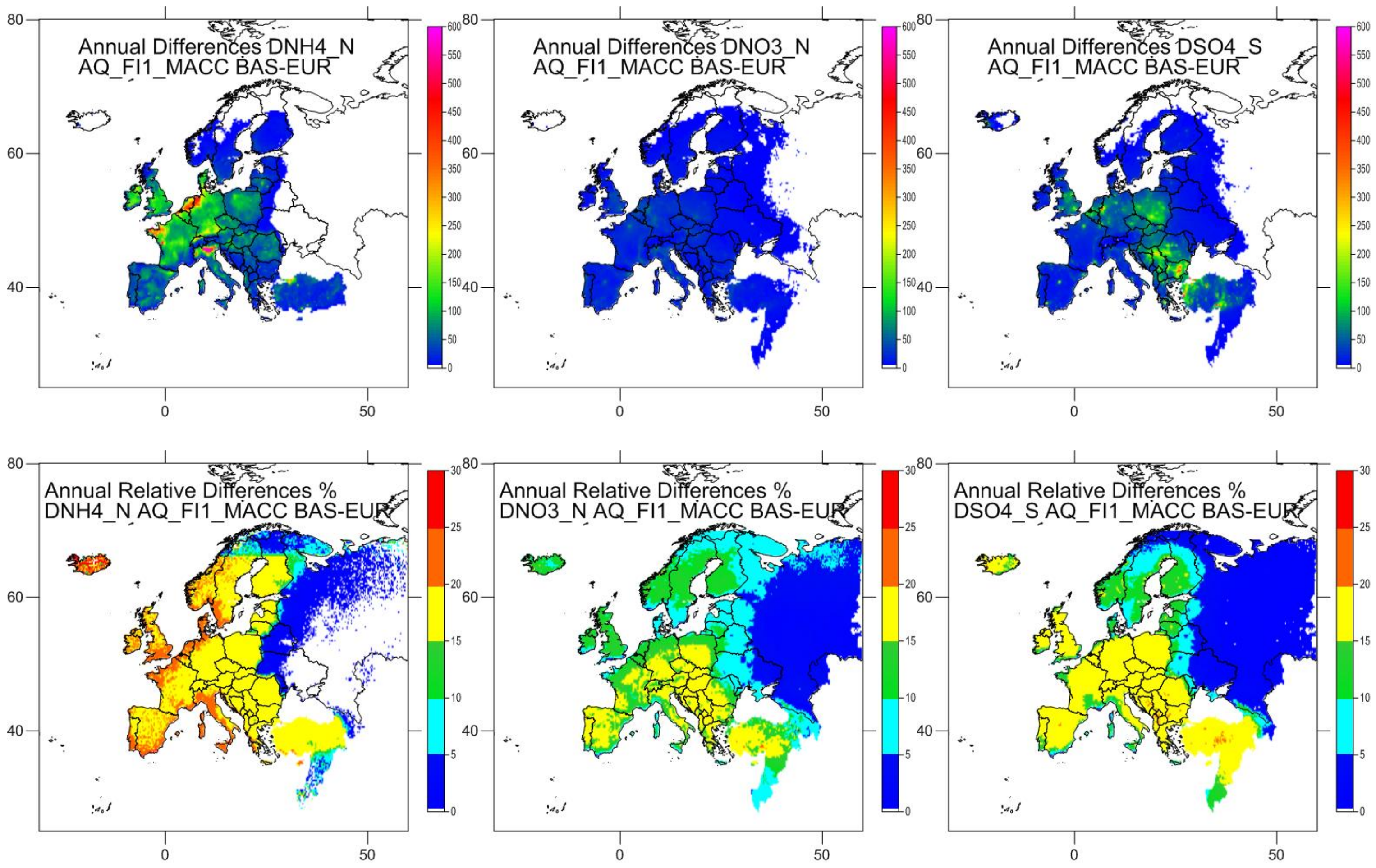
Annual Differences DNH4_N
AQ_FI1_MACC BAS-EUR
Annual Differences DNO3_N
AQ_FI1_MACC BAS-EUR
Annual Differences DSO4_S
AQ_FI1_MACC BAS-EUR
Annual Relative Differences %
DNH4_N AQ_FI1_MACC BAS-EUR
Annual Relative Differences %
DNO3_N AQ_FI1_MACC BAS-EUR
Annual Relative Differences %
DSO4_S AQ_FI1_MACC BAS-EUR
80
60
40
0
50
0 50 100 150 200 250 300 350 400 450 500 550 600
0 5 10 15 20 25 30

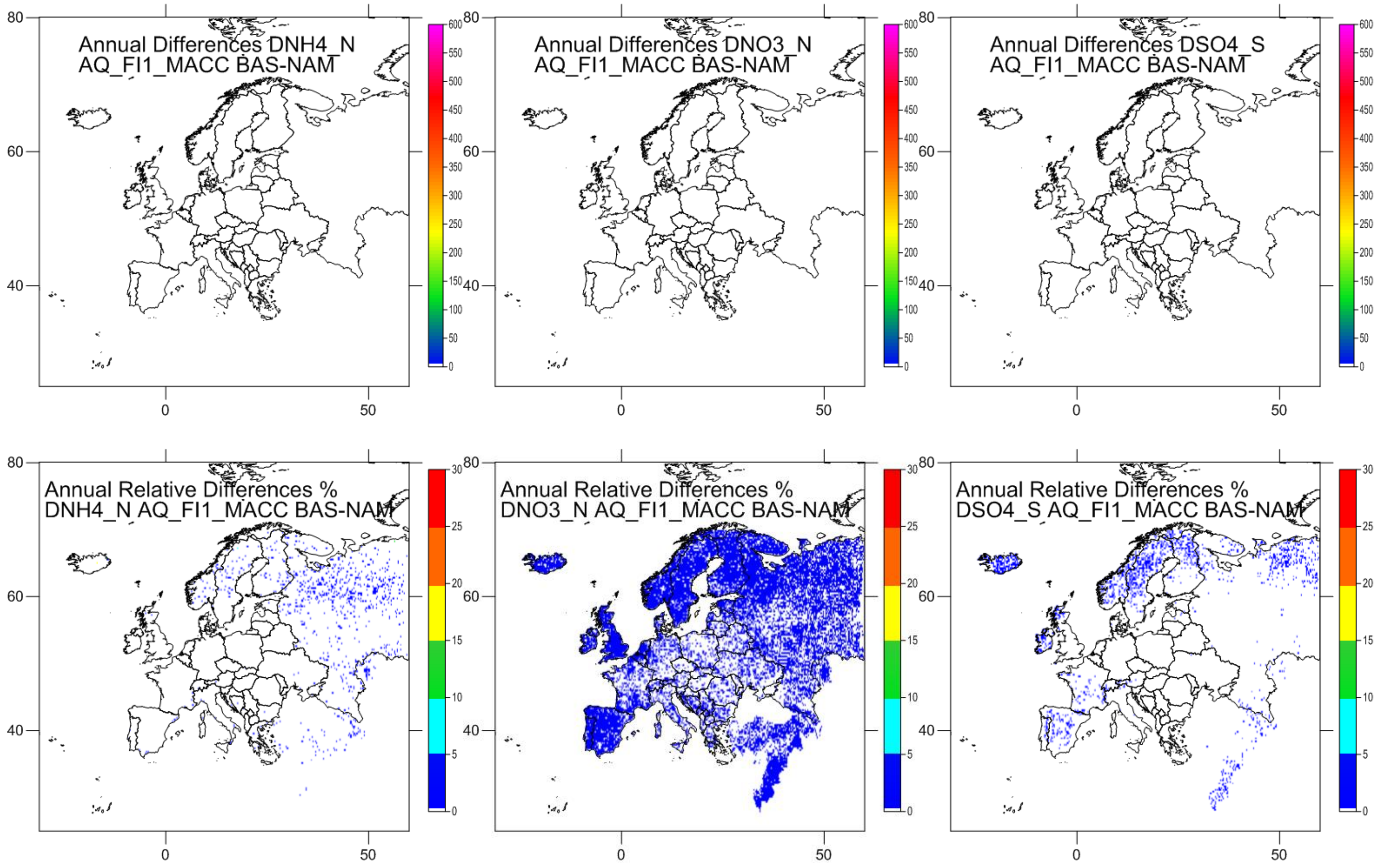
Annual Differences DNH4_N
AQ_FI1_MACC BAS-NAM
Annual Differences DNO3_N
AQ_FI1_MACC BAS-NAM
Annual Differences DSO4_S
AQ_FI1_MACC BAS-NAM
Annual Relative Differences %
DNH4_N AQ_FI1_MACC BAS-NAM
Annual Relative Differences %
DNO3_N AQ_FI1_MACC BAS-NAM
Annual Relative Differences %
DSO4_S AQ_FI1_MACC BAS-NAM
80
60
40
0
50
600
550
500
450
400
350
300
250
200
150
100
50
0
30
25
20
15
10
5
0

S7A. Emissions of NOx, NH3 and SO2 for the AQMEII3/HTAP and ED/TFMM communities

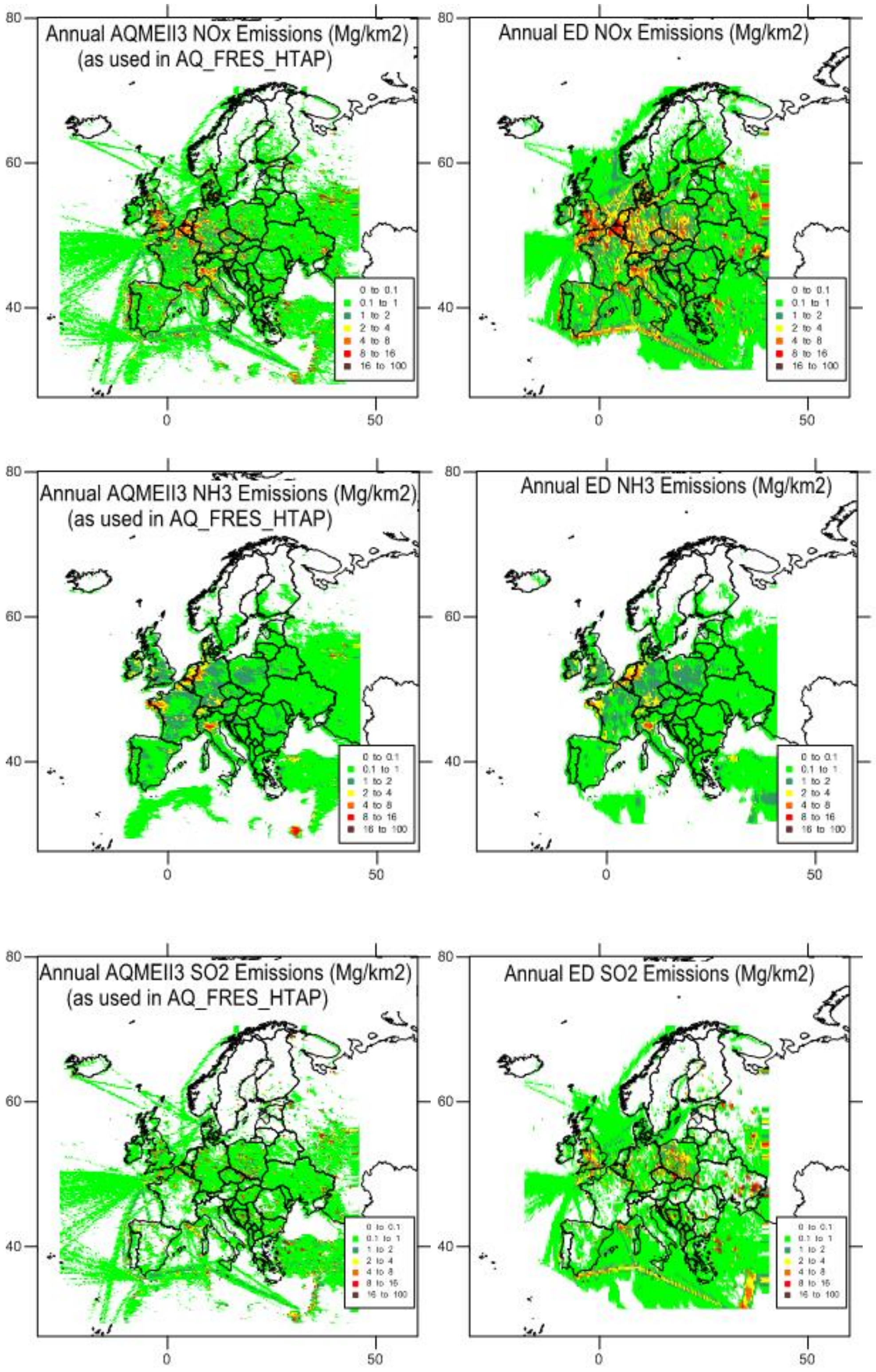

S7B. Differences between the emissions of NOX, NH3 and SO2 of the AQMEII3/HTAP and ED/TFMM communities:

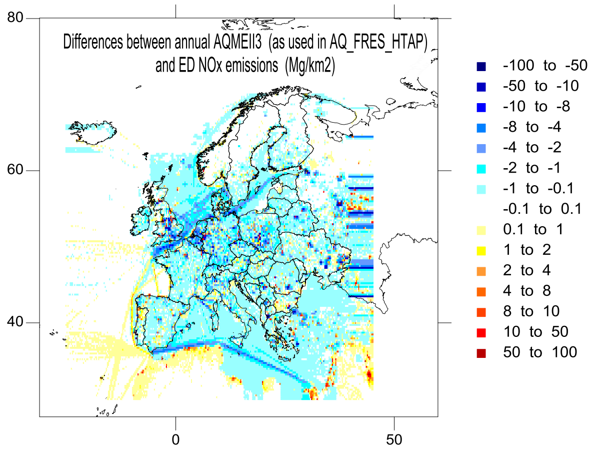

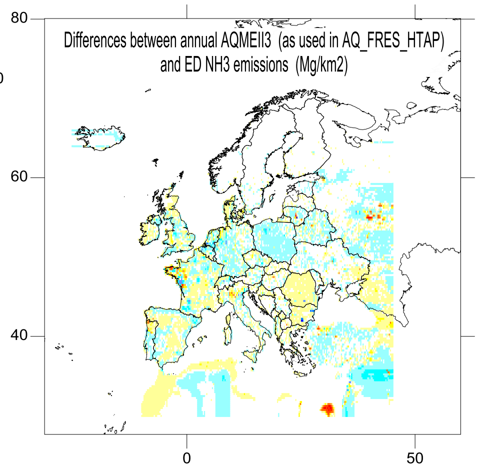

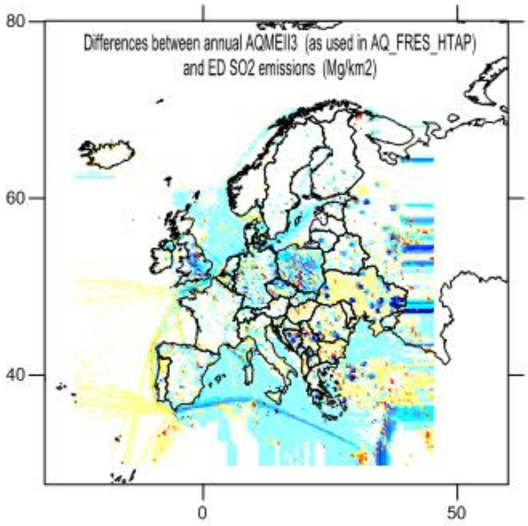

Supplement: Supp [file NIHMS982194-supplement-Supp.pdf]
